# Enhanced semantic classification of microbiome sample origins using large language models (LLMs)

**DOI:** 10.1093/gigascience/giag015

**Published:** 2026-02-12

**Authors:** Daniela Gaio, Janko Tackmann, Eugenio Perez-Molphe-Montoya, Nicolas Näpflin, David Patsch, Lukas Malfertheiner, Matteo Eustachio Peluso, Christian von Mering

**Affiliations:** Department of Molecular Life Sciences and Swiss Institute of Bioinformatics, Winterthurerstrasse 190, University of Zürich, 8057 Zürich, Switzerland; Department of Molecular Life Sciences and Swiss Institute of Bioinformatics, Winterthurerstrasse 190, University of Zürich, 8057 Zürich, Switzerland; Department of Molecular Life Sciences and Swiss Institute of Bioinformatics, Winterthurerstrasse 190, University of Zürich, 8057 Zürich, Switzerland; Department of Molecular Life Sciences and Swiss Institute of Bioinformatics, Winterthurerstrasse 190, University of Zürich, 8057 Zürich, Switzerland; Department of Molecular Life Sciences and Swiss Institute of Bioinformatics, Winterthurerstrasse 190, University of Zürich, 8057 Zürich, Switzerland; Department of Molecular Life Sciences and Swiss Institute of Bioinformatics, Winterthurerstrasse 190, University of Zürich, 8057 Zürich, Switzerland; Department of Molecular Life Sciences and Swiss Institute of Bioinformatics, Winterthurerstrasse 190, University of Zürich, 8057 Zürich, Switzerland; Department of Molecular Life Sciences and Swiss Institute of Bioinformatics, Winterthurerstrasse 190, University of Zürich, 8057 Zürich, Switzerland

**Keywords:** large language model, LLM, GPT, metadata, classification, annotation, FAIR

## Abstract

**Background:**

Over the past decade, central sequence repositories have expanded significantly in size. This vast accumulation of data holds value and enables further studies, provided that the data entries are well annotated. However, the submitter-provided metadata of sequencing records can be of heterogeneous quality, presenting significant challenges for re-use. Here, we test to what extent large language models (LLMs) can be used to cost-effectively automate the re-annotation of sequencing records against a simplified classification scheme of broad ecological environments with relevance to microbiome studies, without fine-tuning. This effort directly contributes to improving the FAIRness—findability, accessibility, interoperability, and reusability—of microbiome sequencing metadata, thereby enhancing their “AI readiness” for downstream computational analyses.

**Results:**

We focused on sequencing samples taken from the environment, for which metadata is important. We employed OpenAI Generative Pre-trained Transformer models, and assessed scalability, time- and cost-effectiveness, as well as performance against a diverse, hand-curated benchmark with 1,000 examples that span a wide range of complexity in metadata interpretation. Annotation performance markedly outperformed that of a baseline, manually curated, non-ML keyword-based approach. Changing models (or model parameters) has only minor effects on performance, but prompts need to be carefully designed to match the task. Furthermore, when we compared proprietary OpenAI models with open-weight alternatives (e.g., Qwen, meta-Llama, and Microsoft-Phi-4), we found comparable accuracy for both biome and sub-biome classification, indicating that open-weight architectures can match the performance of proprietary models for large-scale ecological metadata re-annotation. We validated the pipeline with 1,000 hand-curated samples, and we applied the optimized pipeline to 2 million sequencing records from the environment, providing coarse-grained yet standardized sample origin annotations covering the globe.

**Conclusions:**

Our work demonstrates the effective use of LLMs to simplify and standardize annotation from complex biological metadata.

## Introduction

Streamlining and standardizing metadata is crucial for ensuring reproducibility in scientific research. Over the past decade, the size of the GenBank database has expanded by more than 30-fold, the whole-genome sequencing database grew nearly 40-fold, and the European Nucleotide Archive reported a 10-fold increase in a window from 2012 to 2022 [1]. This vast increase underscores the necessity of managing, standardizing, and utilizing such large datasets effectively. Metadata accompanies all scientific data types, and primary data repositories provide submitters with guidelines and facilities for providing structured metadata at the time of submission. However, the submission step—critical as it is—often does not receive as much attention as earlier steps, such as sample collection and processing. Samples with well-organized metadata are more likely to be reused [2–4], indicating that thoughtful metadata submission enhances broader research utilization. In the case of raw DNA sequence data, the National Center for Biotechnology Information (NCBI) Sequence Read Archive (SRA) submission system suggests a number of metadata fields to be filled, along with controlled vocabularies. However, as is common in many databases, only a minimal number of fields are mandatory, leaving submitters with considerable discretion in how information is provided and formatted. This flexibility, while beneficial for submitters, frequently results in metadata that is challenging to reuse. Similar issues have been reported in public repositories. A recent study documented BioSample records containing incomplete or nonsensical attribute entries such as placeholder tags (“TODO: TAG NAME”), random strings (“ACAGACAGCGT”), or symbols, highlighting how metadata irregularities can render otherwise valuable datasets difficult to interpret and reuse [5]. We observed comparable problems where metadata entries were often verbose, inconsistent, or redundantly encoded across fields (Supplementary Fig. 1). These examples illustrate how even syntactically valid metadata can remain semantically inconsistent, complicating automated parsing and large-scale re-annotation. These challenges directly relate to the FAIR guiding principles, which emphasize that data should be findable, accessible, interoperable, and reusable. While these principles have guided data stewardship for a decade, achieving them in practice remains difficult, particularly for legacy datasets with inconsistent or unstructured metadata. Over time, NCBI has introduced measures to mitigate this issue, such as pre-populated drop-down menus for fields like *organism_type*, and since June 2023, they provide a tutorial to guide submission. While these changes help standardize entries, they are unlikely to completely solve the issue [5]. Moreover, prior submissions that allowed free-form data entry remain difficult to standardize retroactively. Addressing these shortcomings is essential to make large-scale sequencing repositories both, FAIR-compliant and “AI ready,” ensuring that they can support automated discovery and integrative analyses [6].

The rise of artificial intelligence (AI) in metadata parsing marks a significant evolution from earlier efforts using traditional natural language processing (NLP) methods [5, 7–9] or techniques that use term frequency (e.g., term frequency-inverse document frequency, i.e., TF-IDF) [10] to identify key terms within text-based metadata. The complexity of such metadata, which can range from full sentences to acronyms, uses niche terminology and frequently includes spelling variants or typos, posing substantial challenges to traditional NLP and TF methods. Relying solely on TF, even when metadata is articulated clearly, can be inadequate for good classification, as these methodologies lack contextual understanding [11]. For example, if both “Komodo dragon” and “mice” are equally mentioned within different, user-defined fields of a metadata text sample, traditional TF methods may fail to discern that the former refers to the origin of the sample and the latter to the host’s diet. This limitation highlights the need for more sophisticated approaches. Recent advancements in AI have led to the development of robust pre-trained models, particularly large language models (LLMs), which excel at extracting information from diverse and complex metadata across many domains [6, 12–14], including the SRA, where LLMs have been shown to robustly extract experimental attributes like cell lines and target genes [15]. LLMs enhance text mining capabilities by effectively understanding context, making them suited for sophisticated tasks like metadata parsing and mining.

We use *MicrobeAtlas* as a testbed for metadata parsing using LLMs. *MicrobeAtlas* is a large, diverse resource, containing millions of metagenomic SRA samples retrieved from NCBI [16]. MicrobeAtlas uses metadata-extracted keywords to assign samples to defined environmental categories based on hard-coded rules. This non-semantic approach can however fail to assign the correct meaning to terms, particularly in the presence of diverse, user-defined metadata fields, leading to ambiguous or even wrong assignments. Our objective here is to instead leverage general-purpose LLMs for (re-)classification of samples into defined environmental categories, while, simultaneously, retrieving other useful information from the metadata. We classify samples by operational biomes, defined here as broad sample-origin classes used in MicrobeAtlas: “animal,” “plant,” “water,” “soil,” and “other.” This use of the term biome is tailored for metadata reuse and differs from classical ecological biome concepts or ENVO’s biome class; it is a controlled label for source context, not a climato-vegetation unit. We further assign sub-biomes as concise, human-readable refinements of sample origin (e.g., “human gut,” “rhizosphere,” “river water”). The tasks given to the LLMs are (1) classification of samples into biomes; (2) further classification of samples into sub-biomes; (3) extraction of the geographic location of a given sampling site, and (4) extraction of up to 8 key terms describing the sample.

We aim to produce high-quality outputs in a cost- and time-efficient manner by systematically evaluating Generative Pre-trained Transformer (GPT) performance across model versions, conditions, and configurations, and by comparing proprietary (OpenAI GPT) and open-weight alternatives. In addition to benchmarking the pipeline with 1,000 hand-curated sample metadata, we release GPT-parsed metadata for over 2 million samples, providing a valuable resource for the community. More broadly, this work fills a critical gap in metadata reuse by offering an approach that bridges the manual curation of small datasets and the unfiltered use of millions of raw, inconsistently annotated records.

## Methods

To enhance reproducibility and portability, our entire pipeline—including metadata download and processing, benchmark creation, GPT requests, and output validation—is fully containerized using Docker. Users can deploy via a single Docker image [17] and follow detailed usage steps in the GitHub README.md [18].

### Metadata download and processing

**Figure 1:**
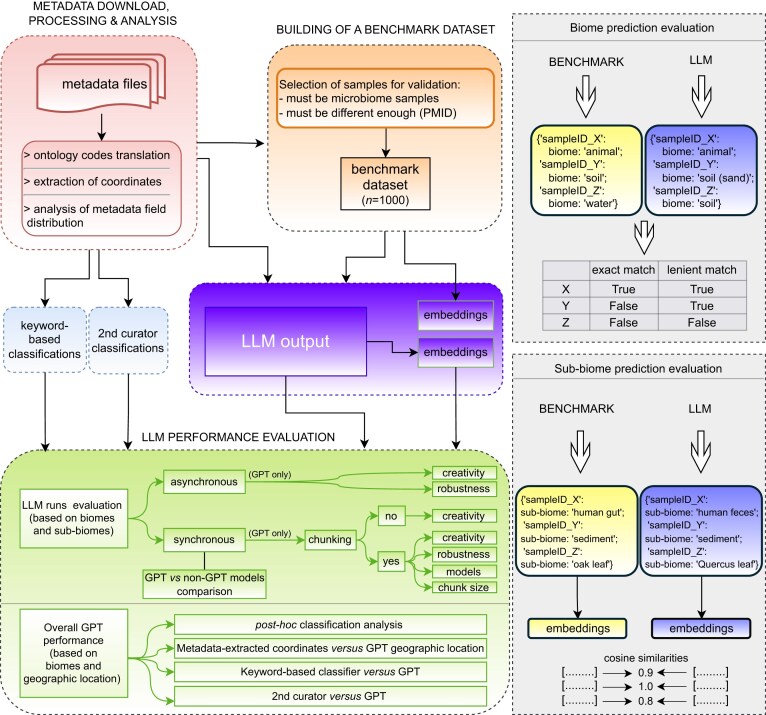
Summarized pipeline. For a detailed pipeline, see Supplementary Fig. 1.

We used MicrobeAtlas as a testbed for LLMs’ metadata extraction. For MicrobeAtlas, the NCBI-SRA had been searched for DNA sequencing runs with metadata keywords matching “metagenomic,” “microb*,” “bacteria,” or “archaea,” and all metadata files for these runs were downloaded in March 2020 [16]. For the present study, all metadata were merged into a single comprehensive metadata file sample.info.gz [19]. This file was then organized on a per-sample basis and sorted into directories named after the last 3 digits of each sample’s name (script: *01_dirs.py*). Environmental, food, plant, species, and cross-species anatomy ontologies (ENVO, FOODON, NCBITaxon, PO, and UBERON) were retrieved, parsed, and subsequently consolidated into a dictionary (script: *fetch_and_join_ontologies.py)*. Metadata files underwent a cleaning process, and ontology terms were converted from their numeric representation to their corresponding textual description, utilizing the ontology dictionary we assembled (script: *clean_and_envo_translate.py*). During cleaning, we removed empty lines and fields containing placeholder texts (e.g., “missing,” “NaN,” “not applicable”), as well as lines that began with “experiment,” which typically include information about wet lab procedures (e.g., sample preparation, sequencing, library preparation, adapter sequences). Log files were maintained in order to track which lines were removed and which ontology codes were converted to their textual counterparts. Coordinates (latitude and longitude) were extracted from the metadata, based on a combination of field names and parseable numeric formats (script: *parse_lat_lon_from_metadata.py*). Occurrence frequencies of major biome-indicating terms within NCBI submission fields were computed (script: *field_distrib_analysis.py*), and the size of metadata files was monitored to ensure they remained manageable for further processing (script: *check_metadata_sizes.py*) (Fig. 1; Supplementary Fig. 2).

### Building a benchmark dataset

To assess the output from the LLMs, we established a benchmark dataset consisting of hand-curated metadata records. This process began with a sub-selection of metadata records from the initial pool of 3.8 million SRA BioSamples and ultimately selected 1 million samples based on the availability of sufficient metadata. From these selected samples, we extracted identifiers such as DOI, PMID, PMCID, and BioProject accession numbers. Utilizing NCBI’s *Entrez* system, we retrieved referenced publications for each sample record, retaining all samples for which at least 1 valid PubMed abstract could be retrieved. In instances where multiple publications were associated with a single sample, we chose the oldest one, assuming it is the most likely primary data reference. However, if the title of the publication contained keywords suggesting it described a protocol, we selected the second-oldest publication. The primary objective of this publication assignment strategy was to ensure that each sample selected for the benchmark was linked to a unique abstract, thereby maximizing the likelihood that the samples within the benchmark dataset were sufficiently distinct from one another. This approach helps avoid potential biases that might arise if multiple samples from the same study are selected, which would result in redundant metadata. The resulting selected samples are provided (training_data_pmid_based.csv; *n* = 669,108) (Fig. 1; Supplementary Fig. 2).

The specific task of the hand curation step was to classify samples into 5 biomes: “animal,” “soil,” “plant,” “water,” and “other,” based on all preprocessed metadata. The term biome here denotes the operational sample-origin class. It differs from ENVO’s biome concept and is intended for robust, automatable text classification. The choice of these biomes was based on *MicrobeAtlas’* pre-existing 2-level ontology, which is what we refer to with “biome” and “sub-biome.” Following *MicrobeAtlas’* choice, rhizosphere samples were curated as *plant* and sediment samples as *water*. Samples with an identifiable origin that did not match any of the 5 biomes were discarded from the benchmark dataset. Sub-biomes were determined based on more detailed manual examinations of all metadata fields. For animal and plant biomes, sub-biomes correspond to the host taxon and the specific part from which the sample is taken. For example, samples from the animal biome may be classified into the sub-biome “human gut,” while those from the plant biome can be categorized as “olive tree leaf.” Water biome samples were described by their water body source (e.g., river, sea, wastewater, ocean, lake). Soil samples were differentiated by soil type (e.g., agricultural, forest, tundra, desert, peatland). Samples with biome category “other” were sorted into the following most frequently encountered sub-biomes: urban, bioreactor, laboratory, feed/food, fungus, air, or other. The sub-biome was assigned by the curator as it was reported in the metadata. For example, if the sample host was described using its scientific or its common name, it was assigned accordingly (Fig. 1; Supplementary Fig. 2).

We describe briefly the “keyword-based classifier” that MicrobeAtlas is currently based on. Keywords are extracted from various fields and processed (e.g., lowercasing, removal of special characters, tokenization). Keywords are matched against environment-specific term sets. For example, keywords “leaf, banana, tree, crop” match with “plant.” When keywords of a sample matched with more than 1 environment-specific term set (e.g., “leaf, banana, tree, insect”), the sample was labeled as “unknown.”

To facilitate a balanced selection of samples across biomes when building the benchmark dataset, we leveraged MicrobeAtlas biome classifications based on the “keyword-based classifier” described above (script: *make_gold_dict.py*). This script retrieves sample metadata, enabling the curator to dynamically assign the most appropriate biome and sub-biome. To allow overrides of biomes or sub-biomes, the script *edit_gold_dict.py* was employed. Additionally, sub-biomes were used in *field_distrib_analysis.py* where they were matched (exact and lenient matches) against the metadata, in order to establish which fields within the metadata were informative of the sample origin (Fig. 1; Supplementary Fig. 2). Finally, the manually curated benchmark dataset consisted of 1,000 samples (200 per biome).

### Requests to LLMs

For synchronous requests to LLMs, a pipeline was built (script: *openai_main.py*), structured into 5 components: (1) setup of a logging system; (2) fetching metadata texts and segmenting them into manageable chunks; (3) executing requests to the OpenAI API (in the case of GPT models) or to the DeepInfra API (for all other models); and (4) an initial parsing of the LLM output, during which sample IDs between input and output are matched. Step (4) allows sample IDs missing from the output or that failed parsing to be requested again (Supplementary Fig. 3A).

For asynchronous interactions with OpenAI, the pipeline involves retrieving the metadata texts, sending batch requests through the API (script: *gpt_async_batch.py*), and retrieving the output up to 24 h later (script: *gpt_async_fetch_and_save.py*) (Supplementary Fig. 3B).

Both synchronous and asynchronous interactions generate a consolidated output file. The name of the output file reports various parameters, such as the number of metadata samples per biome (–*nspb*) randomly selected from the benchmark dataset, and reproducibility via a fixed random seed (–*rs*). Synchronous runs allow for chunking (–*chunking*), where a single request contains the system prompt followed by multiple metadata samples (if chunking is enabled) or a single sample (if disabled). The maximum number of samples fitting into a single chunk depends on chunk size (–*chunksize*), which reflects the total number of tokens that can fit into a single request, including the system prompt. We employed a first-fit decreasing binning mechanism to optimize chunk utilization. This method sorts samples by token count in descending order and packs them into the fewest number of chunks possible, ensuring that each chunk is filled close to its token capacity without exceeding it (script: *openai_02_metadata_processing.py*). Asynchronous requests are never chunked, meaning that 1 sample of metadata per request is sent. Furthermore, users can specify the model (–*model*), the maximum token count (–*maxtokens*), and parameters that determine creativity of chat completion, e.g., temperature (–*temp*), nucleus sampling (–*topp*), frequency penalty (–*freqp*), and presence penalty (–*presp*). The output filename also includes optional text (–*opt_text*) where the user optionally details specifics of the run, the total number of API requests (for synchronous requests), and the timestamp of the output file creation. The designation “batch” within the filename indicates the OpenAI-generated unique ID of an asynchronous request.

To ensure uniformity across all interactions with the LLM, a standardized system prompt was used. This prompt was designed to systematically collect specific information for each sample from its metadata in a structured manner, so as to facilitate a streamlined parsing and analysis of the output. The system prompt was structured as follows:

*We kindly request your expertise in analyzing the following microbial metagenomic samples from their metadata texts*:

- *Please deduce the source category for the sample, choosing from “animal” (including humans), “plant,” “water,” “soil,” or “other.” Your choices are “animal” (incl. human), “plant,” “water,” “soil,” and “other.” Give strictly a concise, single-word label for the sample ID*.
- *Please infer geographical location where the sample was collected, including the country (NOT the coordinates)*.
- *Extract strictly 5–8 keywords descriptive of the sample origin, separated by commas. Put them within curly brackets*.
- *We seek a brief, up to 3-word description of the sample’s specific origin. For “animal” or “plant” sources, please specify the host and part thereof. For “water” samples, the type of water body is sought (e.g., lake, brine, sea, wastewater, etc.). If from “soil,” specify (e.g., agricultural, desert, forest, etc.). If from “other” specify which (e.g., urban, laboratory, feed/food, fungus, air, etc.)*.

*If information is missing, kindly indicate “NA.” Please separate all values with 3 underscores (“___”)*.

*An example response: SRS123456___animal___Los Angeles, USA___{medical, bone fracture, infection, collagen, hospital, intensive care, cast, Staphylococcus epidermidis}___human elbow*.

The prompt above was used in all cases except for asynchronous requests and synchronous requests that are used for a direct comparison with asynchronous requests. For these cases, the LLM is prompted to generate the output in a JSON format (“openai_system_prompt_json.txt”).

To improve the accuracy of LLM classifications for challenging cases (e.g., rhizosphere and sediment samples), we implemented a tailored prompt (“openai_system_better_prompt.txt”). This prompt instructs the LLM to categorize rhizosphere samples as “plant” and sediment samples as “water.” In addition, a human curator was engaged to assess sample classifications using the standard prompt initially and the better prompt in a subsequent round. This approach allowed us to directly compare the performance of the LLM against human curation, particularly for these challenging sample types, and to verify whether targeted prompting improves sample classification. For the comparison of various LLMs (OpenAI vs. open-weight), the tailored prompt was used.

We conducted a series of tests to compare synchronous and asynchronous request methodologies. For synchronous requests, we examined the impact of enabling chunking vs. disabling it, and we assessed how varying the chunk sizes affects the output. Additionally, we evaluate different models, robustness, alongside adjustments in creativity parameters of chat completion, which can influence, among others, term repetition. In scenarios where chunking is disabled, we focus on the effects of tweaking creativity parameters. Our rationale is that, while concatenating multiple metadata texts into a single request might affect output due to term repetition penalties, this effect might differ when each metadata text is sent as a separate request. Similarly, for asynchronous requests, tests to assess performance of different models, as well as their robustness and the effect of tweaking creativity parameters, are performed (Fig. 1).

### Validation statistics

To validate biome classifications, we parsed the output files to extract the “biome” column and compared each LLM-assigned label with the corresponding curator-assigned biome. We distinguished exact matches, where the 2 labels are identical, from lenient matches, where 1 of the 5 allowed biome labels (“animal,” “plant,” “water,” “soil,” “other”) appears as a complete word but is followed by extra clarifying text (e.g., curator: *animal*; GPT output: *animal (human)*). Lenient matches were counted as correct only for benchmarking purposes to capture the model’s occasional tendency to append detail under certain parameter settings, and do not represent fuzzy or approximate matching. For comparisons involving repeated sample IDs across runs, we use McNemar’s test, which is appropriate for paired binary outcomes (True/False). For comparisons across different sample sets, we employ the *t*-test for independent samples. In both scenarios, a Bonferroni correction is applied to adjust for multiple comparisons (script: *validate_biomes_subbiomes.py*) (Supplementary Fig. 2).

Sub-biome validation is less straightforward, given the free-form data entries in the benchmark dataset and the flexible assignments by the LLM. The “sub-biome” column is extracted from the LLM output, and an embedding model is used to generate embeddings for each sample ID from both the LLM sub-biomes and the benchmark dataset sub-biomes (script: *embeddings_from_sb.py*). Unless noted otherwise, sub-biome embeddings were computed with OpenAI *text-embedding-3-small* (1536-D). For model comparisons, we also used *Qwen/Qwen3-Embedding-0.6B* (1024-D), *Qwen/Qwen3-Embedding-4B* (2560-D), and *Qwen/Qwen3-Embedding-8B* (4096-D). All embedding files are compared to calculate cosine similarity (between the LLM- and the curator-assigned sub-biome embedding), providing a quantitative measure of the accuracy of LLM’s sub-biome predictions (script: *validate_biomes_subbiomes.py*). Similarly to biome prediction validation, for runs involving different sample IDs, a *t*-test for independent samples is used. For runs involving the same sample IDs, comparisons are performed using the paired *t*-test, which is suitable for comparing cosine similarity scores (Supplementary Fig. 2).

In order to assess the performance of the LLM in accurately determining the location of sample collection, we use latitudes and longitudes extracted from the metadata (script: *parse_lat_lon_from_metadata.py*). For this analysis, we include only GPT outputs. The latitudes and longitudes are geo-coded to textual descriptions using the geopy library (script: *coord_to_text.py*). String matches between LLM-predicted locations and the geo-coded locations are recorded. For mismatches, latitude and longitude coordinates for the LLM-predicted locations are obtained using the Google Maps API. This allows us to measure the distance between the LLM-predicted coordinates and those extracted from the metadata. Mismatches are categorized based on the distance between these points into the following categories: less than 100, 100–500, 500–1000, 1,000–4,000, and over 4,000 km. Additionally, to determine whether mismatches are due to incorrect LLM predictions or errors in the metadata-derived coordinates, 100 randomly selected samples are manually validated (script: *geo_check.py*) (Supplementary Fig. 2).

We conduct a comprehensive analysis of LLM performance by aggregating outputs across various experimental parameters (e.g., chunking, (a)synchronicity of requests, and creativity parameters) (script: *overall_analysis.py*). For this analysis, we include only GPT outputs. The script compiles all GPT output files, merging them into a dataset for comparative analysis against curator-assigned biomes (benchmark dataset). The lenient match accuracy is calculated to assess the accuracy of biome predictions both overall and within specific biome categories. The script also compares the GPT-predicted classifications with MicrobeAtlas’s previous biome predictions (based on the “keyword-based classifier”) to determine whether GPT has improved our sample classification (Supplementary Fig. 2).

To contextualize the accuracy of LLM-based classification, we conducted a comparative assessment between LLM and human performance. For this test, we only included GPT outputs as representative of LLMs. We randomly selected 250 samples from the benchmark dataset and provided the corresponding metadata to a trained molecular biologist with no prior exposure to this project. The human annotator was asked to assign biomes and sub-biomes to the samples using the same system prompt initially used for GPT (see the “Requests to LLMs” section). In a second round, both the human annotator and GPT were presented with an improved version of the prompt. The revised prompt included a clarifying instruction: “Please note that rhizosphere samples should be categorized as ‘plant’ and sediment samples as ‘water’.” This addition was made to assess whether explicit guidance improves classification accuracy for ambiguous cases. We evaluated and compared the performance of both classifiers—GPT and human—under each prompt condition (standard vs. “better”). This experiment allowed us to understand how well GPT performs relative to an uninitiated but scientifically literate human, and whether targeted prompting can effectively steer, not only human, but also machine classification.

## Results

To provide an overview of the analyses presented below, we first benchmarked GPT-based classification of microbiome samples against a manually curated dataset and a keyword-based reference system. GPT achieved markedly higher biome-level accuracy, precision, and *F*1-scores than the keyword-based approach, with performance approaching that of a trained human annotator. We then systematically evaluated factors influencing model performance, including prompt design, model version, output format, creativity parameters, and computational strategies such as chunking and asynchronous querying. These analyses revealed that simple prompt refinements improved accuracy, while large chunk sizes (i.e., multiple sample metadata per request) reduced it. Across GPT model versions, GPT-4 and later models provided slightly higher sub-biome annotations quality, particularly when using structured JSON output. We also assessed GPT’s ability to infer sample geographic origins, finding high agreement (∼96%) between GPT-extracted and metadata-derived locations. Finally, we extended our analysis beyond OpenAI models, showing that recent open-weight models (e.g., *Qwen3/Qwen-80B-A3B-Instruct*) achieve comparable accuracy at substantially lower cost, underscoring that both proprietary and open-weight LLMs can support large-scale, FAIR-aligned metadata re-annotation. The following sections provide detailed results and methodological context for each of these analyses, including, at last, an examination of which metadata fields most frequently capture sample origin information. All accuracies reported below refer to the operational biome labels and sub-biome labels defined in the “Introduction” and “Methods” sections, not classical ecological biomes.

### GPT overall performance

**Figure 2:**
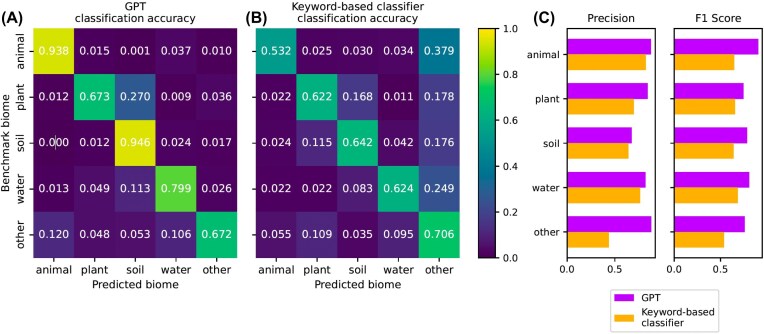
Heatmaps of biome classification accuracy (an average over all GPT runs; *n* = 105). Accuracy rates between benchmark and GPT-predicted biome classifications and between benchmark and the keyword-based biome classifications are shown in the respective heatmaps. Precision and *F*1-score bar plots are shown on the right. Notably, the “unknown” category from the previous classification is aligned with “other” from GPT predictions, for direct comparison. The heatmaps are normalized by row. The benchmark dataset consists of *n* = 1,000 samples. Overall biome accuracy: 80.6% (GPT); 62.5% (keyword-based classifier). Cohen’s kappa: 0.760 (GPT); 0.530 (keyword-based classifier).

When testing the classification performance of our LLM-based classifier (GPT) on our manually curated benchmark dataset (*n* = 1,000 samples, 200 per biome), using a carefully crafted prompt (see the “Methods” section), we found that the model reached an average biome classification accuracy of 80.6% (precision: 82.0%, *F*1-score: 80.34). This performance was robust to variations in GPT run modes and parameters (*n* = 105; accuracy: 76.0–84.1%; precision: 77.1–85.8%; *F*1-score: 76.4–84.0%) and was achieved without any example-driven fine-tuning of the model. When examined separately by biome, the accuracy shows variability. “Soil” and “animal” biome samples show the highest accuracy with accuracy rates of 94.6 and 93.8%, respectively. In contrast, “plant,” “water,” and “other” biome samples exhibit lower accuracy rates (67.3, 79.9, and 67.2%, respectively) (Fig. 2).

Importantly, GPT markedly outperformed MicrobeAtlas’s current, keyword-based classification system (“keyword-based classifier”) in terms of biome prediction accuracy (80.6 vs. 62.5%), precision (81.9 vs. 67.3%), and *F*1-scores (80.3 vs. 63.5%). We observed the largest performance gain for animal samples (accuracy: 93.8 vs. 53.2%, precision: 87.8 vs. 82.4%; *F*1-score: 90.7 vs. 64.7%), which the keyword-based classifier frequently bins into “unknown.” Both methods struggle with plant samples, which they often misclassified as soil (Fig. 2A, B). Whereas GPT most often misclassified them as “soil” (27.0%), the keyword-based classifier repartitioned the misclassified samples into either “soil” (16.8%) or “other” (17.8%). Overall, GPT predictions resulted in a more balanced distribution across biomes and a better alignment with expected frequencies, as indicated by Cohen’s kappa values (GPT: 0.760; keyword-based classifier: 0.530) (Fig. 2).

### Analysis of GPT misclassified samples

The overall 19.4% of incorrect predictions led us to investigate whether specific samples are consistently misclassified in predictable ways. Detailed analysis of all GPT misclassifications confirms a subset of samples with significant biases among misclassified biomes (chi-squared: 7482; *P*-value < 0.001). A large fraction of misclassified “animal” samples (53.0%) are erroneously predicted as “water.” In contrast, the majority of misclassifications within “plant” (83.9%) and “water” samples (59.0%) are “soil” (Supplementary Fig. 4).

The distribution of misclassifications per sample is right-skewed (skewness: 1.34; kurtosis: 0.27), indicating that most samples have relatively few misclassifications compared to a small subset with disproportionally high values (Supplementary Fig. 5). Notably, 21 samples (5.3% of the total) fall at or above the 95th percentile, with 90 or more misclassifications, which is consistent with a right-skewed distribution. Across 105 independent classification runs using different parameter settings, the average number of misclassifications per run is 21.7 (SD: 29.9). Half of the samples are misclassified 4 times or fewer, and 3-quarters have no more than 34 misclassifications. The maximum observed was 97, indicating that some samples were misclassified in almost every run, highlighting extreme cases where GPT predictions consistently disagreed with the curator-assigned biome (Supplementary Fig. 5). A detailed examination of such cases includes a bioreactor digester wastewater sample (SRS994677), a mock community sample (SRS2217033), and 2 rhizosphere samples (SRS5304049 and SRS942824). The bioreactor digester wastewater sample, categorized as “other” by the curator, was incorrectly assigned to “water” by GPT in 89 out of 97 instances. The other 8 were correctly assigned as “other.” The mock community from a gut microbiome study should have been categorized as “other” due to its laboratory nature, while it was erroneously assigned to “animal” by GPT in 90 out of 93 instances. The other 3 were correctly assigned. The 2 rhizosphere samples were misclassified as “soil” by GPT in all but 1 case.

### Human vs. GPT classification accuracy

To evaluate how close GPT’s performance comes to the upper limit of achievable accuracy, we compared it to that of a human annotator. A trained molecular biologist, with no prior exposure to the project, was given the same prompt instructions as GPT and was asked to classify sample biomes and sub-biomes. While against the benchmark dataset, GPT achieved an accuracy of 79.76% (*n* = 499; SD = 40.0), the human annotator reached 78.0% (*n* = 250; SD = 42.0).

In a second round, both GPT and the human were given the same set of samples to classify, this time using an improved prompt (here referred to as “better prompt”). The only change was an added instruction: “Please note that rhizosphere samples should be categorized as ‘plant’, and sediment samples as ‘water’.” With the better prompt, both classifiers showed improved performance. GPT’s accuracy increased to 83.17% (SD = 37.0), while the human annotator reached 88.0% (SD = 33.0).

There was an improvement in accuracy between the human’s first attempt (with the initial prompt) and the human’s second attempt (better prompt) (adj *P*-value ≤ 0.001). No significantly different performances were detected for the sub-biome classification, neither between GPT and the human, nor between prompt versions (adj *P*-value = 1).

### Resource-saving pipeline settings and their impact on GPT output quality

One way to reduce GPT costs is by minimizing the token count submitted to the API, which we achieved by eliminating fields that are empty or have non-informative placeholders such as “NaN” and “unknown.” While the input had to be lengthened in some areas (e.g., environment ontology, i.e., ENVO codes, needed to be converted from numeric IDs to their English text counterparts), we overall achieved a 35% decrease in the size of the input data. Specifically, for the GPT-3.5-turbo-0125 model, which as of October 2025 charged $0.5 per million input tokens, a 35% reduction in tokens results in the cost for processing 2 million samples dropping from ~$522 to $340.

**Figure 3:**
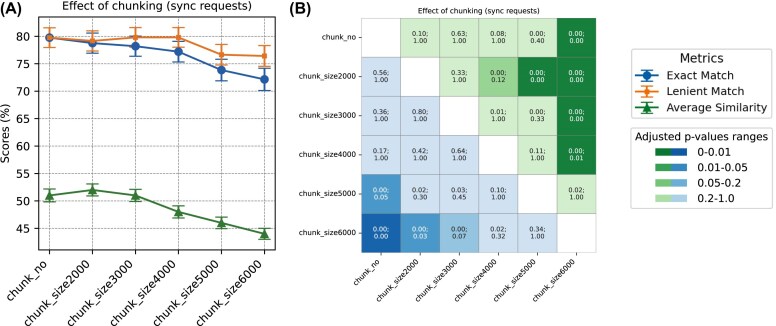
Performance comparison of GPT requests with different chunking sizes. (A) Accuracy percentages for biome prediction are shown, reflecting both exact matches and lenient matches between the GPT-generated output and the curator-assigned biomes. The similarity for sub-biome prediction is represented through average cosine similarity. Chunk size (2,000 up to 6,000) refers to the number of tokens within a single request, i.e., chunk. The label “chunk_no” refers to no chunking, where metadata from a single sample is sent in a single request. (B) *P*-values (top of each cell) and adjusted *P*-values (bottom of each cell) of the performance comparisons are displayed. Cells shaded in green represent the statistical significance of biome accuracy comparisons, while those in blue denote the significance of sub-biome similarity comparisons. The color intensity varies according to the *P*-value significance. McNemar’s and paired *t*-tests were performed for biome and sub-biome prediction comparisons, respectively. Bonferroni correction was applied on *P*-values.

Instead of sending metadata for individual samples in separate requests, we explored grouping multiple samples’ metadata into single requests (chunking) to reduce the token overhead of repeatedly specifying the input/system prompt. We compared the cost-effectiveness of chunking vs. no chunking, and evaluated whether chunking affected performance. We evaluated both biome as well as sub-biome prediction. Our initial findings show no significant performance difference between mild chunking (i.e., chunk size 3,000: median of 5, mean of 6, maximum of 17 samples per request) and no chunking (1 sample per request). This was consistent across both biome prediction (adj *P*-value = 0.56) and sub-biome prediction (adj *P*-value = 1) (Fig. 3B).

Biome prediction starts to slightly worsen when using larger chunk sizes: biome accuracies for chunk sizes of 2,000, 3,000, 4,000, 5,000, and 6,000 tokens per chunk (median sample counts of 3, 5, 7, 9, and 15, respectively) were 78.76, 78.2, 77.2, 73.85, and 72.15% (Fig. 3A). The noticeable drop in accuracy starting with chunk size 6,000 was significant compared to chunk size 2,000 (adj *P*-value = 0.03) (Fig. 3B). Additionally, cosine similarity between benchmark dataset sub-biomes and predicted sub-biomes starts to lower significantly with chunk size 5,000 (average cosine similarity: 0.46) compared to chunk size 2,000 (average cosine similarity: 0.52) (adj *P*-value = 0.00115), indicating that GPT is worse at predicting sub-biomes at higher chunk sizes (Fig. 3B).

### GPT models and output formats

**Figure 4:**
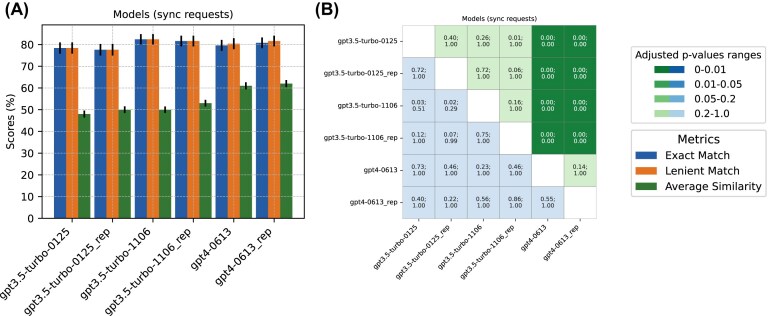
Performance comparison of GPT models. (A) Accuracy scores for biome prediction are shown, reflecting both exact matches and lenient matches between the GPT-generated output and the curator-assigned biomes. The similarity for sub-biome prediction is represented through average cosine similarity. (B) *P*-values (top of each cell) and adjusted *P*-values (bottom of each cell) of the performance comparisons are displayed. Cells shaded in green represent the statistical significance of biome accuracy comparisons, while those in blue denote the significance of sub-biome similarity comparisons. The color intensity varies according to significance. Each run had a replicate (suffix “rep”). McNemar’s and paired *t*-tests were performed for biome and sub-biome prediction comparisons, respectively. Bonferroni correction was applied on *P*-values.

We compared the performance of 3 OpenAI models: *GPT-3.5-turbo-0125, GPT-3.5-turbo-1106*, and *GPT-4-0613* by sending synchronous requests to each model. The accuracy of biome predictions is found consistent across all models, with no statistically significant differences (adj *P*-value ≥ 0.05) (Fig. 4). However, we observed differences in the cosine similarity between benchmark dataset sub-biomes and predicted sub-biomes. Specifically, *GPT-4-0613* demonstrated a slightly yet significantly higher cosine similarity compared to both *GPT-3.5-turbo-0125* (adj *P*-value ≤ 0.001) and *GPT-3.5-turbo-1106* (adj *P*-value ≤ 0.001) (Fig. 4B).

**Figure 5:**
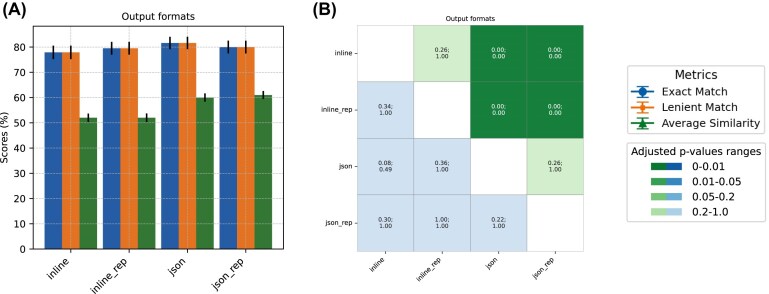
Performance comparison of *GPT-3.5-turbo-1106* using either inline or JSON output format. (A) Accuracy scores for biome prediction are shown, reflecting both exact matches and lenient matches between the GPT-generated output and the curator-assigned biomes. The similarity for sub-biome prediction is represented through average cosine similarity. (B) *P*-values (top of each cell) and adjusted *P*-values (bottom of each cell) of the performance comparisons are displayed. Cells shaded in green represent the statistical significance of biome accuracy comparisons, while those in blue denote the significance of sub-biome similarity comparisons. The color intensity varies according to the *P*-value significance. McNemar’s and paired *t*-tests were performed for biome and sub-biome prediction comparisons, respectively. Bonferroni correction was applied on *P*-values.

To assess the impact of different output formatting on GPT’s performance, we designed 2 system prompts that instruct GPT to format its output either inline (using double underscores to separate answers for each sample), or in JSON format (by stating it in the prompt and by specifying the “format” parameter in the API request). While biome prediction accuracy remained constant across both formats, we observed an improvement in sub-biome cosine similarity when responses were formatted in JSON (adj *P*-value < 0.001) (Fig. 5).

### The effect of tweaking GPT creativity parameters

**Figure 6:**
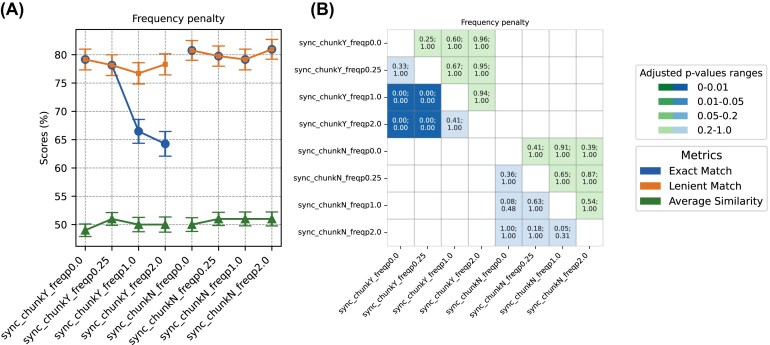
Performance comparison of *GPT-3.5-turbo-1106* when tweaking frequency penalty. (A) Accuracy scores for biome prediction are shown, reflecting both exact matches and lenient matches between the GPT-generated output and the curator-assigned biomes. The similarity for sub-biome prediction is represented through average cosine similarity. (B) *P*-values (top of each cell) and adjusted *P*-values (bottom of each cell) of the performance comparisons are displayed. Cells shaded in green represent the statistical significance of biome accuracy comparisons, while those in blue denote the significance of sub-biome similarity comparisons. The color intensity varies according to the *P*-value significance. For an assessment of all other creativity parameters, see Supplementary Fig. 3. McNemar’s and paired *t*-tests were performed for biome and sub-biome prediction comparisons, respectively. Bonferroni correction was applied on *P*-values.

Parameters such as temperature, nucleus sampling, and penalty settings influence how deterministic or diverse the outputs of LLMs are, and thus could potentially affect prediction. Adjustments to temperature, nucleus sampling, or presence-penalty settings did not impact the prediction accuracy for biome or sub-biome categories (adj *P*-value ≥ 0.05) (Supplementary Fig. 6). Accuracy consistently dropped with increasing frequency penalty, from an initial 79.2% (freqp 0.0) to a minimum of 64.3% (freqp 2.0). This decline was significant (adj *P*-value < 0.001) (Fig. 6). However, when the more lenient criterion for string matching of the GPT output with the benchmark dataset biome was applied (i.e., “lenient match”), the impact of increased frequency penalty on biome accuracy was mitigated (freqp 0.0: 79.16%; freqp 2.0: 78.31%) (Fig. 6A). By examining GPT answers, it was clear that a higher frequency penalty led GPT to deviate from strictly adhering to the prompt instructions “Answer with exactly one word from the following….” Instead, the model tended to provide unsolicited information, e.g., instead of providing “animal” for an answer, it provided “animal (incl. human).” In fact, biome accuracy under the lenient matching condition remained unaltered with differing frequency penalties.

Remarkably, significant differences across creativity parameters were detected only in the case of chunked requests. Without chunking, neither synchronous requests nor asynchronous requests showed significant differences (Supplementary Fig. 6). This indicates that the tweaking of creativity parameters, specifically frequency penalty, affects the output only when multiple samples’ metadata are submitted in a single request.

### GPT synchronous and asynchronous requests

To assess the reliability of different querying strategies in large-scale automated analyses, we compared the robustness of synchronous (without chunking) and asynchronous requests. We conducted 10 replicate tests for each: 500 randomly selected metadata samples were sent individually to 10 synchronous runs or 10 asynchronous runs (replicates 1–10). For synchronous requests, the biome accuracy (lenient match) did not vary (range = 78.2–80.8), and neither did the sub-biome cosine similarity (range = 0.49–0.51) (Supplementary Fig. 7A, D). Asynchronous requests demonstrated similar consistency for biomes accuracy (lenient match) (range = 81.8–83.2), and no significant variations across different runs for sub-biome predictions (range = 0.56–0.58) (adj *P*-value = 1) (Supplementary Fig. 7B, E). Note that the higher accuracies for asynchronous requests here are due to the different output format used for each.

We evaluated the performance differences between synchronous and asynchronous requests (*n* = 6 replicates each) in terms of both biome and sub-biome prediction accuracies, keeping all parameters equal (including the output format). Our analysis revealed that whether requests are sent synchronously or asynchronously does not correlate with a better prediction of biomes or sub-biomes (adj *P*-value = 1) (Supplementary Fig. 7C, F).

### Geographic location prediction performance

**Figure 7:**
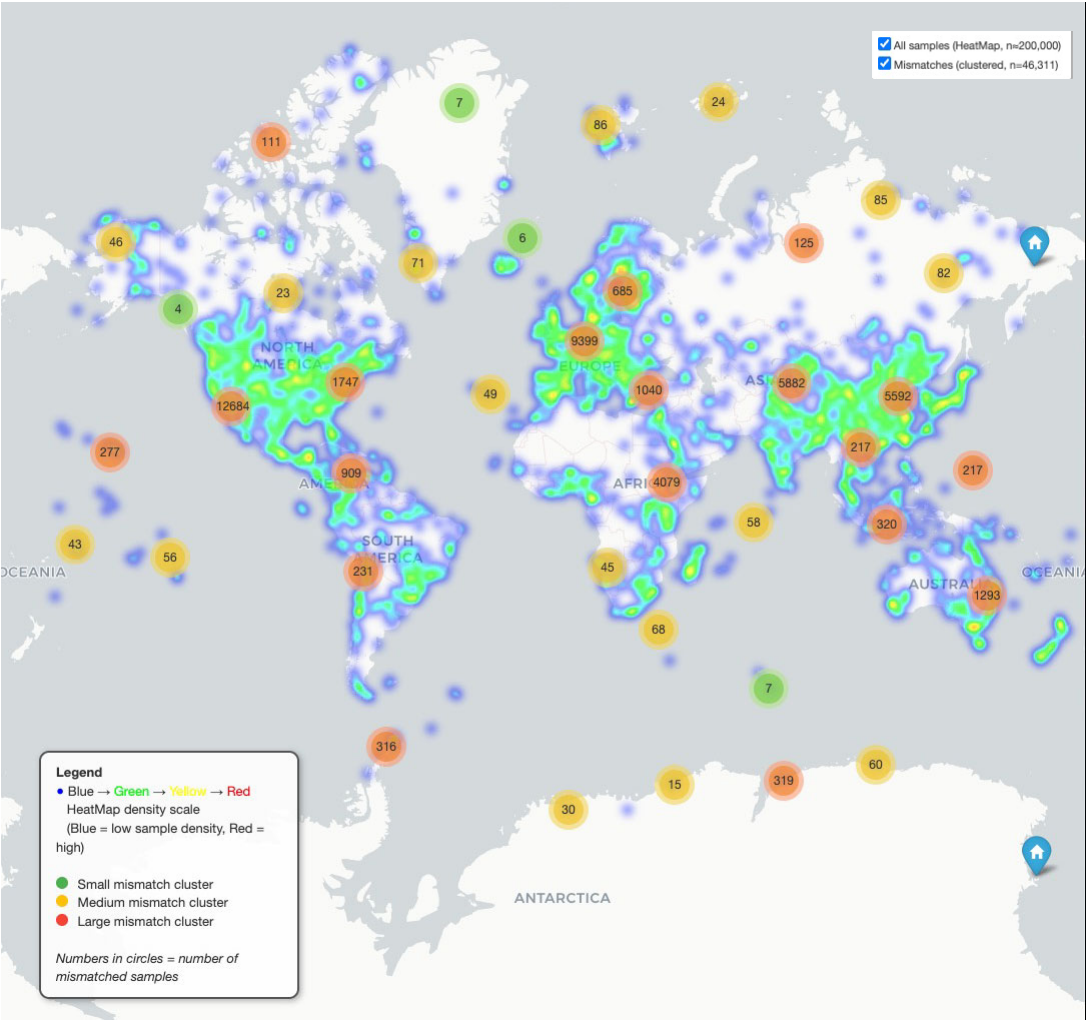
Global distribution of microbiome samples and geographic annotation consistency. A heatmap depicts the global density of a subset of 200,000 microbiome samples (from a pool of 990,172), with warmer colors indicating regions of higher sampling intensity. Overlaid circular clusters mark samples where GPT-inferred geographic locations did not match coordinates extracted from the metadata. Cluster color indicates the number of mismatched samples. Together, these layers highlight both the uneven geographic distribution of microbiome sampling and the spatial patterns of metadata inconsistencies. The interactive figure can be downloaded from here.

During the production run, we instructed *GPT-3.5-turbo-0125* to extract the geographical location where each sample was collected, including the country (in textual format, i.e., not in latitude/longitude coordinates). We measure the performance by comparing the output with textual descriptions derived directly from lat/lon coordinates parsed from the metadata. Among 990,172 samples analyzed (of over 2 M), 95.32% matched the geo-coded location. The distribution of the matching and mismatching samples can be visualized on a global map (Fig. 7).

To further analyze the 4.68% mismatches (*n* = 46,311), we obtained the actual coordinates for the GPT-predicted locations using the Google Maps API and assessed the distance between these and the metadata-derived coordinates. The distances ranged from 0.6 to 19,856 km (first quartile: 244.7 km, third quartile: 10,294.4 km). The distribution of the mismatching samples, color-coded by distance categories, can be visualized on a global map (Supplementary Fig. 8). For a focused investigation, a subset of 100 misclassified samples, randomly picked and with a discrepancy above 1,000 km between the GPT-predicted locations and the metadata-derived coordinates, was examined manually. This analysis revealed that

- In half of the cases, GPT correctly predicted the location, while the lat/lon coordinates parsed from the metadata were incorrect. This happened for various reasons: the coordinates in the metadata were either completely wrong (30%), lat/lon were swapped (7%), had wrong longitudinal signs (negative [34%] or positive [5%]), had wrong latitudinal signs (negative, 9%), a wrong latitude (5%), or the coordinates pointed at the academic institution of the authors rather than to the location where the sample was collected (7%).
- In 27% of the cases, both GPT and coordinates pointed at the right geographic location, but the name given by GPT did not textually match with the geographic location from the coordinates (e.g., McMurdo Station vs. Antarctica). This often happened with coastal samples of the United States, where GPT returns “United States.”
- In 16% of the cases, the coordinates pointed to the right location, but GPT did not extract it correctly. This happened because GPT mistook the institute location for the sampling location (41%), the coordinates pointed to a water body or an island (e.g., “Hawaii”), while GPT predicted the location after the country of belonging of that water body (e.g., “United States”) (18%), or the metadata did not clearly mention the location in text format (41%).
- In 7% of the cases, metadata-extracted coordinates and GPT geolocation did not match because neither information was retrievable from the metadata as it was ambiguous or absent.

### How does GPT compare with non-OpenAI models?

To evaluate whether OpenAI’s GPT models perform comparably to other state-of-the-art LLMs, we benchmarked *GPT-3.5-turbo-1106, GPT-4.1*, and *GPT-5-mini* against a representative set of open-weight models, including *meta-llama/Meta-Llama-3.1-8B-Instruct, Qwen/Qwen3-Next-80B-A3B-Instruct, Microsoft-Phi-4*, and *Mistral-Nemo-Instruct-2407*. All models were prompted identically using the optimized “better prompt” (see the “Methods” section), ensuring a fair comparison across models.

**Figure 8:**
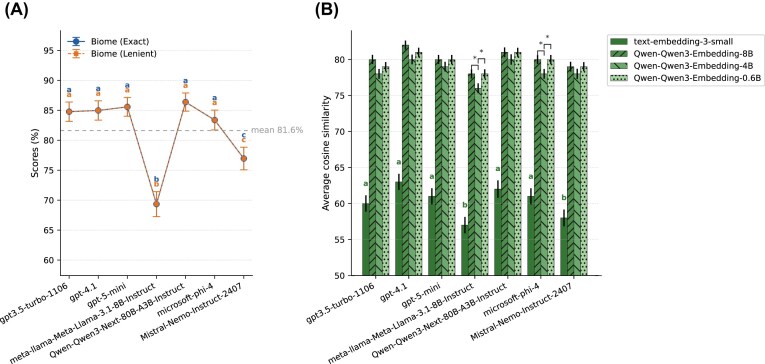
Comparison of LLMs and embedding models for biome and sub-biome classification performance. (A) Accuracy of biome predictions across models, shown as both exact and lenient matches between LLM-generated outputs and curator-assigned biome annotations. Lowercase letters (a–c) above points indicate results of *post hoc* groupings: models that share the same letter (e.g., all marked “a”) do not differ significantly, whereas models with different letters (e.g., “a” vs. “b” or “c”) show statistically significant differences in accuracy (McNemar’s test, Bonferroni-adjusted *P* < 0.05). (B) Average cosine similarity between sub-biome annotations generated by the LLMs and converted into embeddings using 4 different embedding models. Statistical significance for biome predictions was assessed using McNemar’s test, and for sub-biome similarities using paired *t*-tests. Bonferroni correction was applied to adjust *P*-values for multiple comparisons.

Biome accuracy was similar across models, with several models forming a single *post hoc* group (Fig. 8A). *Qwen3-Next-80B-A3B-Instruc*t achieved the highest observed accuracy for exact/lenient biome agreement (86.37%), very slightly above GPT-5-mini (85.57%), *GPT-4.1* (84.97%), *GPT-3.5-turbo-1106* (84.77%), and *Microsoft-Phi-4* (83.37%); however, these differences did not reach statistical significance after *post hoc* correction (letters “a” in Fig. 8A). In contrast, *Mistral-Nemo-Instruct-2407* (76.95%) and especially *Meta-Llama-3.1-8B-Instruct* (69.34%) lagged behind the other models (Fig. 8A).

For sub-biome similarity, results were broadly consistent across embedding models—the LLM ranking was preserved and differences within a given LLM were modest (Fig. 8B). Using *text-embedding-3-small* as the embedding model, *GPT-4.1* and *Qwen3-Next-80B-A3B-Instruct* achieved the top scores (0.63 and 0.62, respectively), followed by *GPT-5-mini* and *Microsoft-Phi-4* (both 0.61) and *GPT-3.5-turbo-1106* (0.60). *Mistral-Nemo-Instruct-2407* (0.58) and *Meta-Llama-3.1-8B-Instruct* (0.57) had the lowest mean scores. Pairwise testing reflected these gaps: *Mistral-Nemo-Instruct-2407* was significantly below *GPT-4.1* (adj *P*-value = 0.00128), and *Meta-Llama-3.1-8B-Instruct* was significantly below *Qwen3-Next-80B-A3B-Instruct* (adj *P*-value < 0.001) and *Microsoft-Phi-4* (adj *P*-value = 0.03816).

With respect to embedding model choice, pooling across LLMs, *Qwen* embedding models outperformed *text-embedding-3-small* (adj *P*-value < 0.001). *Qwen3-Embedding-8B* resulted in the highest average similarity and was not statistically different from *Qwen3-Embedding-4B* across models (adj *P*-value > 0.05), while *Qwen3-Embedding-0.6B* was significantly lower than both *Qwen3-Embedding-8B* and *Qwen3-Embedding-4B* in several model comparisons (e.g., *Meta-Llama-3.1-8B-Instruct* and *Microsoft-Phi-4*; adj *P*-value < 0.05). Notably, the embedding models used in these analyses differed in dimensionality: *text-embedding-3-small* produces 1,536-dimensional vectors, whereas *Qwen3-Embedding-0.6B, Qwen3-Embedding-4B*, and *Qwen3-Embedding-8B* produce 1,024-, 2,560-, and 4,096-dimensional vectors, respectively. Together, these results indicate that embedding choice substantially raises or lowers the absolute cosine values (*Qwen* embeddings score notably higher than *text-embedding-3-small* [adj *P*-value < 0.001]), but it does not alter the relative LLM ranking: *GPT-4.1* and *Qwen3-Next-80B-A3B-Instruct* remain at the top, with *Meta-Llama-3.1-8B-Instruct* and *Mistral-Nemo-Instruct-2407* at the bottom. At the biome level, OpenAI’s GPT models perform strongly, but several non-OpenAI models, particularly *Qwen3-Next-80B-A3B-Instruct* and, for embeddings, *Qwen3-Embedding-8B* or *Qwen3-Embedding-4B*, achieve comparable or better accuracy, respectively, indicating that high-quality biome and sub-biome annotation can be achieved with both proprietary and open-weight architectures, expanding options for large-scale ecological metadata re-annotation.

In addition to comparable performance, open-weight models offer a substantial cost advantage. Based on current pricing (input/output tokens per million), their costs are typically 10–50 times lower than those of proprietary GPT models. For example, *Qwen3-Next-80B-A3B-Instruct, Meta-Llama-3.1-8B-Instruct*, and *Microsoft-Phi-4* are priced at ~$0.03–0.14 per million input tokens and $0.05–1.10 per million output tokens, compared with $1.00–2.00 for input and $2.00–8.00 for output tokens for OpenAI’s *GPT-3.5-turbo-1106* and *GPT-4.1-mini*. Although the open-weight models themselves are free of cost, the costs mentioned here reflect fees from third-party hosting platforms (e.g., DeepInfra) used to access the models, since running large open-weight models locally requires dedicated GPU hardware.

### Informative metadata fields

Not all metadata fields contain information about sample origins, but as field names are not always standardized, it can be difficult to know *a priori*, which fields to parse and focus on. By programmatically comparing the metadata of 1,000 samples to their curator-assigned sub-biomes, we determined which fields typically report the sample origin. We found that the sample origin was documented in 67 distinct fields when considering full matches (e.g., “cow rumen”) and in 127 distinct fields when considering lenient matches (e.g., “cow” or “rumen”). On average, the full origin of a sample is found in 1.36 fields per sample (SD: 1.67), and at least part of the sample origin is reported in 3.0 fields (SD: 2.2) per sample, indicating a modest variability in how sample origins are documented across different metadata fields.

Some fields are more commonly used than others to report the origin of the sample. The fields most frequently containing sample origin information are “study_STUDY_ABSTRACT” (267 instances), “sample_SCIENTIFIC_NAME” (225 instances), “study_STUDY_TITLE” (212 instances), “sample_isolation_source” (113 instances), and “study_STUDY_DESCRIPTION” (53 instances). Despite their relevance for mentioning sample origins, the fields “study_STUDY_ABSTRACT” and “study_STUDY_DESCRIPTION” are more challenging to parse given their average word count (72.8 and 144.8, respectively) compared to “sample_SCIENTIFIC_NAME” (2.12), “sample_isolation_source” (2.9), and “study_STUDY_TITLE” (8.9).

The variability in field usage does not seem to be arbitrary, but appears influenced by the biome associated with the sample. For example, sample origin is more likely to be found under the field “sample_host” in the case of animal and plant samples, while the fields “sample_env_biome” and “sample_env_feature” are more likely to contain useful information in the case of soil, water, and “other” samples. This demonstrates that certain fields are preferentially selected to report sample origin, depending on the sample biome (Supplementary Fig. 9).

## Discussion

A central goal of data repositories is to enable the effective secondary use of research data, potentially leading to new discoveries beyond the original scope of the study. In practice, however, most datasets are rarely re-analyzed by researchers outside the submitting laboratory. A major barrier to reusability is poor metadata, often incomplete, inconsistent, or non-standardized, which limits automated parsing and interpretation. As a result, the full potential of many datasets remains untapped. Recent systematic assessments confirm this trend: nearly half of microbiome studies fail to meet minimum metadata reporting standards, despite sharing sequence data, underscoring the persistent lack of standardization and interoperability in the field [20]. Two strategies come to mind to address this issue: (1) enforcing more rigorous reporting standards at the time of submission and (2) retroactively improving metadata quality using computational tools. While ongoing efforts have improved metadata submission guidelines, these changes are difficult to enforce consistently and do not address the vast amount of legacy data already in repositories. In contrast, LLMs offer a flexible and scalable means to retrospectively annotate and standardize metadata, even when it is noisy or poorly structured. In this study, we evaluated the use of LLMs for metadata re-annotation of environmental sequencing samples. To this scope, we used proprietary OpenAI models as well as several open-weight models, and we compared their performance.

When evaluating LLM performance in classifying environmental sequencing samples, we found that it consistently outperformed a traditional keyword-based classifier. Unlike the latter, which relies on static keyword matching and extensive hard-coded whitelists, LLMs leverage context and semantics to disambiguate terms based on their usage and surrounding information within the metadata. Importantly, our approach achieved this performance without any fine-tuning, demonstrating that general-purpose text generation models can perform complex metadata re-annotation tasks effectively. This contrasts with task-specific approaches such as *ChIP-GPT*, where a model was fine-tuned on curated examples to extract metadata from SRA records with comparable accuracy [15].

To contextualize model performance, we compared GPT’s classifications with those of a trained molecular biologist unfamiliar with the project. Both were presented with the same metadata and prompt, and their classifications were evaluated against the benchmark. Under standard conditions, GPT and the human annotator achieved similar accuracy. When given a clearer, more explicit prompt, both improved. This demonstrates not only GPT’s capacity to perform at a near-expert level for this classification task, but also highlights the importance of precise instructions—an aspect that benefits both human and machine annotators. These findings align with observations by Sundaram et al. (2025) [21], who showed that prompts incorporating explicit metadata schema information markedly improved LLM output consistency and completeness [21].

However, LLMs are not without their limitations. One challenge is the need to translate ontology accession codes—commonly found in metadata—into human-readable terms, as LLMs do not interpret these codes natively. This step of ontology translation directly supports the *Interoperability* component of FAIR by aligning textual metadata with standardized vocabularies such as ENVO and FOODON [21]. This approach parallels the template-augmented prompting approach of Sundaram et al. (2025), where domain-specific schema and structure guide LLMs toward outputs that are consistent with metadata standards and reduce semantic inconsistencies [21]. Secondly, LLMs do not always “read between the lines.” For instance, if a sample is part of an animal study but correctly described as a mock community, the LLM may still classify it as “animal” rather than “other.” Prompt adherence is also imperfect: if the metadata points frequently at the term “soil,” the LLM may default to assigning that biome, regardless of more nuanced cues. This was especially evident for rhizosphere and sediment samples, which were major contributors to biome misclassification. These cases illustrate a broader challenge: sample classification in environmental data is often inherently ambiguous. A sediment sample taken from a coastal area, e.g., could reasonably belong to either “soil” or “water” depending on phrasing. Similar issues affected the keyword-based classifier, which also showed bias in such edge cases. These examples underscore the difficulty of resolving ambiguity in free-text metadata, regardless of the method applied.

Taken together, the ~20% misclassification rate observed with LLMs likely reflects an upper limit of LLM performance, rather than a true failure rate. Given the unavoidable ambiguity and challenges in re-annotating diverse samples against a necessarily limited and simplified classification scheme, even a second human curator might not agree with the benchmark labels. This suggests that even LLMs operating at human level may not be sufficient for perfect categorization, especially in domains like environmental sampling where a certain amount of overlap is to be expected. This is consistent with findings from other domains, where LLMs can extract complex biological attributes with high accuracy yet still require human verification and post-processing to correct misapplied or ambiguous entries [22].

An alternative to direct classification is to prompt the model to generate a brief description of the sample’s origin and then derive embeddings from these summaries. These embeddings can be compared to those generated from curator-provided descriptions. This approach allows for more flexible sample grouping: even if a sample is misclassified at the biome level, its sub-biome description—e.g., “mock community”—may still position it correctly in embedding space alongside similar samples. In this study, we used such comparisons primarily to validate the LLM output via cosine similarity scores, but the same method could also support clustering or reclassification of ambiguous samples.

Tweaking GPT “creativity parameters” such as temperature, nucleus sampling, and presence penalty had no impact on biome or sub-biome prediction accuracy. However, increasing the frequency penalty led to reduced biome accuracy—but only when multiple samples were included in a single request (i.e., chunking). This effect disappeared when samples were submitted individually. A likely explanation is that repeated use of a term like “animal” within a chunk triggers the penalty, prompting GPT to vary its wording (e.g., returning “animal (human)” instead). Supporting this, the accuracy drop vanished when using lenient string matching. Interestingly, rather than avoiding penalized terms altogether, GPT tended to add detail to them. Aside from frequency penalty effects during chunking, no other creativity parameter significantly affected classification performance.

We also assessed the impact of request mode, model version, and output format on GPT’s performance. As expected, there was no difference between synchronous and asynchronous requests, since both use the same underlying model. Given this, asynchronous requests are currently preferable, as they are easier to manage at scale and are more cost-effective. Model choice, however, did influence output: *GPT-4-0613* outperformed both *GPT-3.5-turbo-1106* and *GPT-3.5-turbo-0125* in sub-biome prediction. Output format also had an effect. While biome predictions were unchanged, sub-biome accuracy improved significantly when using JSON rather than inline formatting. This likely reflects the benefit of structured, machine-readable output, which reduces ambiguity and promotes consistent generation. Although both formats yielded similar recall, the structured format may help the model better focus on the task, possibly by encouraging more consistent output or reducing ambiguity during generation. Ensuring consistent and machine-readable output is critical for scalable downstream analysis. Similar challenges have been documented in other LLM-based workflows, e.g., in morphological extraction tasks where unexpected HTML or LaTeX formatting required repeated re-queries [22], and in systems like ChIP-GPT where outputs sometimes include unsolicited content or deviate from strict formatting [15].

As the use of GPT’s API is invoiced based on the number of input and output tokens, we attempted to reduce costs by (1) cleaning away uninformative fields from the metadata and (2) sending multiple samples’ metadata under the same request (i.e., chunking). The preliminary cleaning of the metadata proved valuable, reducing input token size by 35%. However, we observed diminishing returns at larger chunk sizes. We hypothesized that sending too many samples at once might “confuse” the model and tested the limit of this strategy. In our case performance declined significantly at 5,000 tokens per request (roughly 9 samples) while remaining stable at 2,000 tokens (about 3 samples). A minor decline was visible at 4,000 tokens (7 samples), but not statistically significant. Although we did not test chunking with asynchronous requests, we have no reason to believe the results would differ. It is important to note that these thresholds may vary for different metadata types.

Our analysis of 1,000 samples demonstrated that the sample origin typically appears in 1–3 fields but spans across no fewer than 127 distinct field types, underscoring the immense variability in how metadata is reported. The best field for inferring the broad origin of a sample is the study_STUDY_ABSTRACT field, which is also the most verbose and thus challenging to process. The sample_SCIENTIFIC_NAME field often reflects the sample origin in host-associated metagenomic samples and is more concise in length. Overall, the usefulness of specific fields varies across biomes, suggesting that a one-size-fits-all selection of metadata fields would not perform equally well across all sample types. This variability is likely influenced not only by user practices but also by differences in the BioSample package system used for submission. Historical or package-specific requirements may have further contributed to the uneven distribution of field usage across biomes. This highlights the persistent challenges in standardizing metadata extraction, despite ongoing efforts to regulate SRA submissions.

We also used GPT to extract the geographic location of each sample’s collection site. This was useful in cases where latitude/longitude coordinates were missing, incorrect, or inconsistently formatted. Issues included swapped coordinates, decimal errors, or identical shifts across samples of the same study due to submitters using the drag-copy function in Excel. In many such cases, GPT successfully inferred the sampling country or locality based on free-text metadata, achieving 95% accuracy. Among mismatches, about half were due to incorrect metadata coordinates, while a quarter were semantic mismatches (e.g., “Antarctica” vs. “McMurdo Station”). In a fifth of the cases, GPT misidentified the location, either due to confusing institution names with sampling sites or due to vague metadata. A small fraction of samples had no retrievable geographic information at all.

To determine whether our findings were specific to proprietary GPT models or broadly applicable across architectures, we compared OpenAI models with several open-weight LLMs. This comparison is relevant for reproducibility, cost, and long-term accessibility, as open-weight models can be self-hosted and remain usable independent of commercial APIs. Performance was comparable across models: OpenAI’s GPTs remained strong, but the open-weight *Qwen3-Next-80B-A3B-Instruct* achieved similar accuracy for biome and sub-biome annotation. Importantly, these open-weight models achieved comparable performance at a fraction of the cost—typically 10–50 times lower than proprietary GPT models—further supporting their scalability for large-scale annotation. Embedding choice was also relevant, with 3 Qwen embedding models (0.6B, 4B, and 8B) outperforming OpenAI’s *text-embedding-3-small*. This is unlikely due to higher dimensionality alone, as the 1,024-dimensional Qwen3-Embedding-0.6B outperformed the 1,536-dimensional OpenAI’s *text-embedding-3-small* model. Importantly, within the Qwen family, performance did not scale monotonically with size: the 0.6B model consistently exceeded the 4B, whereas the 8B was best overall, suggesting that training objectives, corpus alignment, and potentially lower overfitting in smaller models, rather than embedding dimensionality, are the main drivers of performance. Overall, these results suggest that open-weight LLMs have reached a maturity level that enables reliable, cost-effective, large-scale metadata re-annotation while improving reproducibility and sustainability [5, 22].

While our analyses demonstrate the utility of LLMs for metadata re-annotation, the present study focuses on a limited subset of metadata fields—specifically those related to sample origin, including *biome, sub-biome*, and geographic location. These fields were selected because they are among the most informative yet inconsistently reported attributes in microbiome studies, and because they serve as strong indicators of sample context, a key determinant of ecological interpretation. Other metadata categories, such as sequencing methods, host characteristics, or experimental conditions, were not systematically evaluated here. Their structure, controlled vocabularies, and interdependencies may introduce different types of ambiguity or standardization challenges. Accordingly, the current findings should be viewed as a proof of concept demonstrating that LLMs can successfully handle complex, text-rich metadata fields. Extending these approaches to other metadata domains will require additional benchmarking and potentially more specialized prompt designs or schema-aware models.

In conclusion, our results indicate that LLMs already operate at near-human accuracy for structured re-annotation tasks—at least in relatively structured tasks like microbiome sample origin classification. Errors still occur, partly due to model limitations and the inherent ambiguity of real-world samples, and occasionally due to parsing issues, an observation consistent with reports that complex metadata descriptions, schema limitations, and computational constraints continue to challenge full automation [5]. More straightforward technical constraints include rate limits imposed by commercial APIs and token limits per request. However, open-weight architectures alleviate many of these constraints and, as demonstrated in this study, now demonstrate performance equivalent to proprietary models for ecological metadata annotation. They also offer greater scalability and flexibility, since the models themselves are freely available and can be run locally or on institutional hardware. Additionally, open-weight models can be run repeatedly to build consensus across predictions, increasing confidence in the annotations. In practice, most users currently rely on third-party platforms such as DeepInfra or Hugging Face to access sufficient GPU resources, which may still incur computational costs, but these are substantially lower than those associated with proprietary model providers. This balance between openness, affordability, and performance positions open-weight models as a sustainable option for large-scale metadata re-annotation. Recent work applying LLMs to BioSample metadata has shown similar benefits, with model-assisted extraction markedly improving the findability and reusability of experimental records [5]. Together, these findings demonstrate that LLMs—including open-weight architecture—provide a practical and scalable foundation for FAIR-compliant metadata re-annotation in microbiome research, improving data interoperability and enabling future discoveries.

## Availability of source code and requirements

Project name: metadata_mining.

Project homepage: https://github.com/GaioTransposon/metadata_mining.

Operating system: Linux, macOS, and Windows (via Docker).

Programming language: Python.

Other requirements: Docker (gaiotransposon/metadmin) (all scripts and dependencies are packaged in a Docker image; see repository README for execution instructions).

## Additional files

**Supplementary Figure S1**. Examples of heterogeneous and semantically inconsistent metadata records illustrating challenges for automated parsing: metadata fields containing embedded taxonomic tables and verbose narrative text within descriptive fields (top), redundant or conflicting information distributed across multiple metadata fields (middle), inconsistent formatting, mixed delimiters, and variable completeness across metadata attributes (bottom).

**Supplementary Figure S2**. Pipeline in detail. C1–C4 denote the containers. Detailed instructions on how to reproduce the workflow using the containers can be found here.

**Supplementary Figure S3**. Pipeline of synchronous (A) and asynchronous (B) requests. Synchronous and asynchronous pipelines have been used for all OpenAI models, whereas requests to all other LLMs were done synchronously.

**Supplementary Figure S4**. Misclassifications by GPT.

**Supplementary Figure S5**. Distribution of sample misclassifications by GPT.

**Supplementary Figure S6**. Performance comparison of GPT when tweaking creativity parameters: temperature (temp), nucleus sampling (topp), frequency penalty (freqp), and presence penalty (presp). (A–D) Accuracy scores for biome prediction are shown, reflecting both exact matches (blue line) and lenient (orange line) matches between the GPT-generated output and the curator-assigned biomes. The similarity for sub-biome prediction is represented through average (cosine) similarity (green line). (E–H) *P*-values (top of each cell) and adjusted *P*-values (bottom of each cell) of the performance comparisons are displayed. Cells shaded in green represent the statistical significance of biome accuracy comparisons, while those in blue denote the significance of sub-biome similarity comparisons. The color intensity varies according to the *P*-value significance. McNemar’s and paired *t*-tests were performed for biome and sub-biome prediction comparisons, respectively. Bonferroni correction was applied on *P*-values.

**Supplementary Figure S7**. Performance reproducibility. (A–C) Accuracy scores for biome prediction are shown, reflecting both exact matches and lenient matches between the GPT-generated output and the curator-assigned biomes. The similarity for sub-biome prediction is represented through average (cosine) similarity. (D–F) *P*-values (top of each cell) and adjusted *P*-values (bottom of each cell) of the performance comparisons are displayed. Cells shaded in green represent the statistical significance of biome accuracy comparisons, while those in blue denote the significance of sub-biome similarity comparisons. The color intensity varies according to the *P*-value significance. McNemar’s and paired *t*-tests were performed for biome and sub-biome prediction comparisons, respectively, when the sample groups being compared were the same (same “rs,” i.e., random seed). When comparing different sample groups, independent *t*-tests were applied. Bonferroni correction was applied on *P*-values.

**Supplementary Figure S8**. Spatial distribution of samples showing GPT–metadata location mismatches. Each point marks a sample for which GPT-inferred location and metadata-derived coordinates differed. Colors denote increasing distance between the 2 locations (blue ≤100 km; green 100–500 km; yellow 500–1,000 km; orange 1,000–4,000 km; red >4,000 km). Circle size reflects the number of overlapping samples. In the interactive figure, clicking on any point displays detailed information, including the original metadata-derived location, the GPT-inferred name, the geographic distance between them, and the number of samples represented. The map illustrates the geographic extent of annotation inconsistencies and identifies regions with frequent long-distance mismatches. The map displays all mismatches (*n* = 46,311) from a pool of 990,172 samples.

**Supplementary Figure S9**. Metadata field distribution. Frequency of metadata fields matching benchmark sub-biomes by biome category. Fields with less than 20 partial matches are filtered out.

## Supplementary Material

giag015_Supplemental_File

giag015_Authors_Response_To_Reviewer_Comments_original_submission

giag015_GIGA-D-25-00316_original_submission

giag015_GIGA-D-25-00316_revision_1

giag015_Reviewer_1_Report_original_submission Reviewer 1 -- 9/5/2025

giag015_Reviewer_2_Report_original_submission Reviewer 2 -- 9/10/2025

giag015_Reviewer_2_Report_revision_1 Reviewer 2 -- 11/10/2025

giag015_Reviewer_3_Report_original_submission Reviewer 3 -- 9/14/2025

giag015_Reviewer_3_Report_revision_1 Reviewer 3 -- 11/28/2025

## Data Availability

All data necessary to run the containers can be downloaded from Zenodo [19]. All outputs and results of biome and sub-biome validation and all results from the comparisons performed (biome_subbiome_results*.csv, and biome_subbiome_stats*.csv, respectively) are available under the same Zenodo link. All output from the production run (>2 M samples) is available under the “production” directory, also available under the same Zenodo link. File descriptions are provided. Interactive map figures are downloadable from Zenodo [23, 24].
