# Supplementary material for: Enhanced semantic classification of microbiome sample origins using large language models (LLMs)
Source: Gigascience. 2026 Feb 12;15:giag015. doi: 10.1093/gigascience/giag015 (PMC13042274; doi:10.1093/gigascience/giag015)

Figure 1. Summarized pipeline. For a detailed pipeline see Supplementary Figure 1.

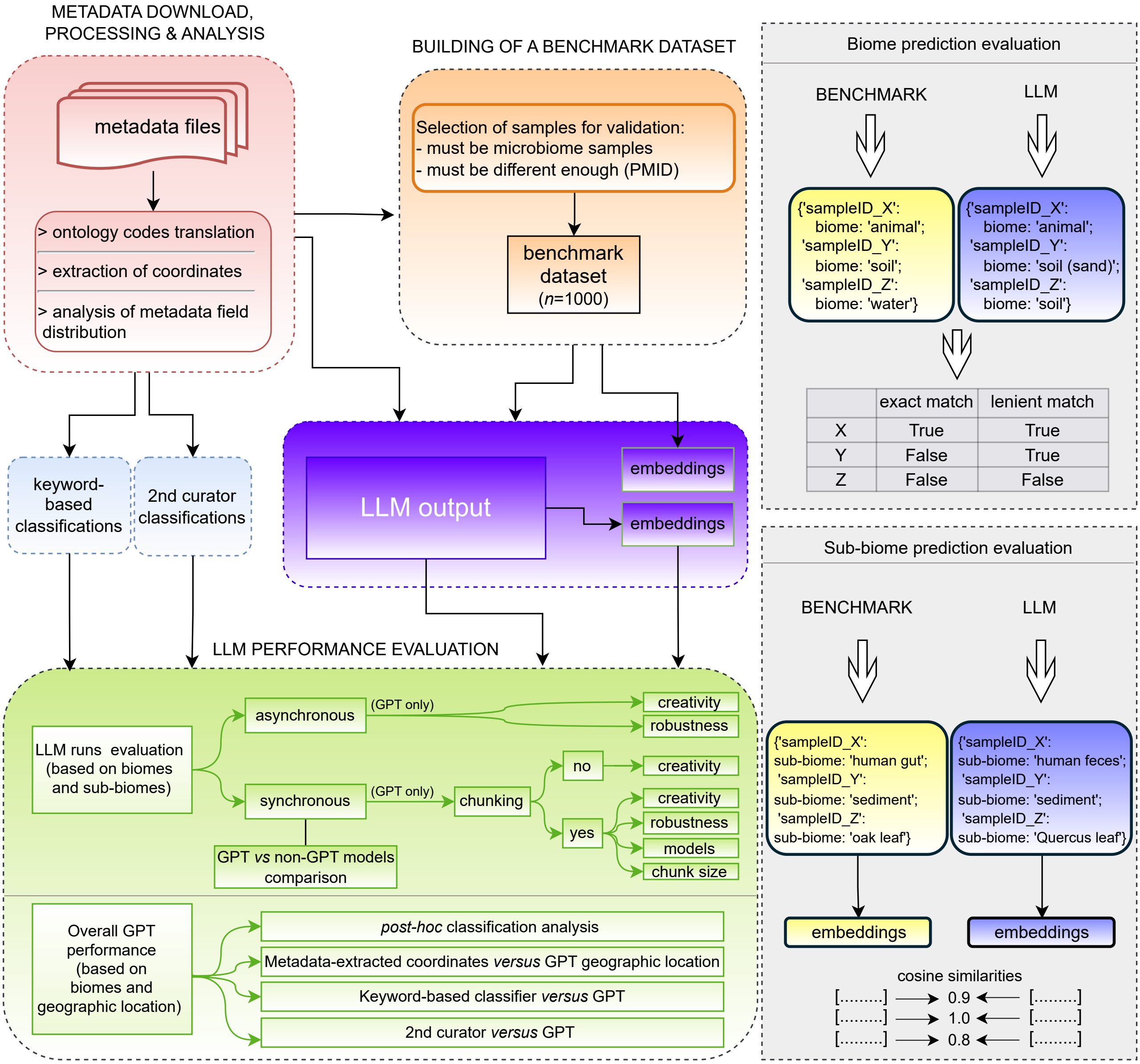

Figure 2. Heatmaps of biome classification accuracy (an average over all GPT runs; n=105). Accuracy rates between benchmark and GPT predicted biome classifications and between benchmark and the keyword-based biome classifications are shown in the respective heatmaps. Precision and F1-score bar plots are shown on the right. Notably, the 'unknown' category from the previous classification is aligned with 'other' from GPT predictions, for direct comparison. The heatmaps are normalized by row. The benchmark dataset consists of n=1,000 samples. Overall biome accuracy: 80.6% (GPT); 62.5% (Keyword-based classifier). Cohen's Kappa: 0.760 (GPT); 0.530 (Keyword-based classifier).

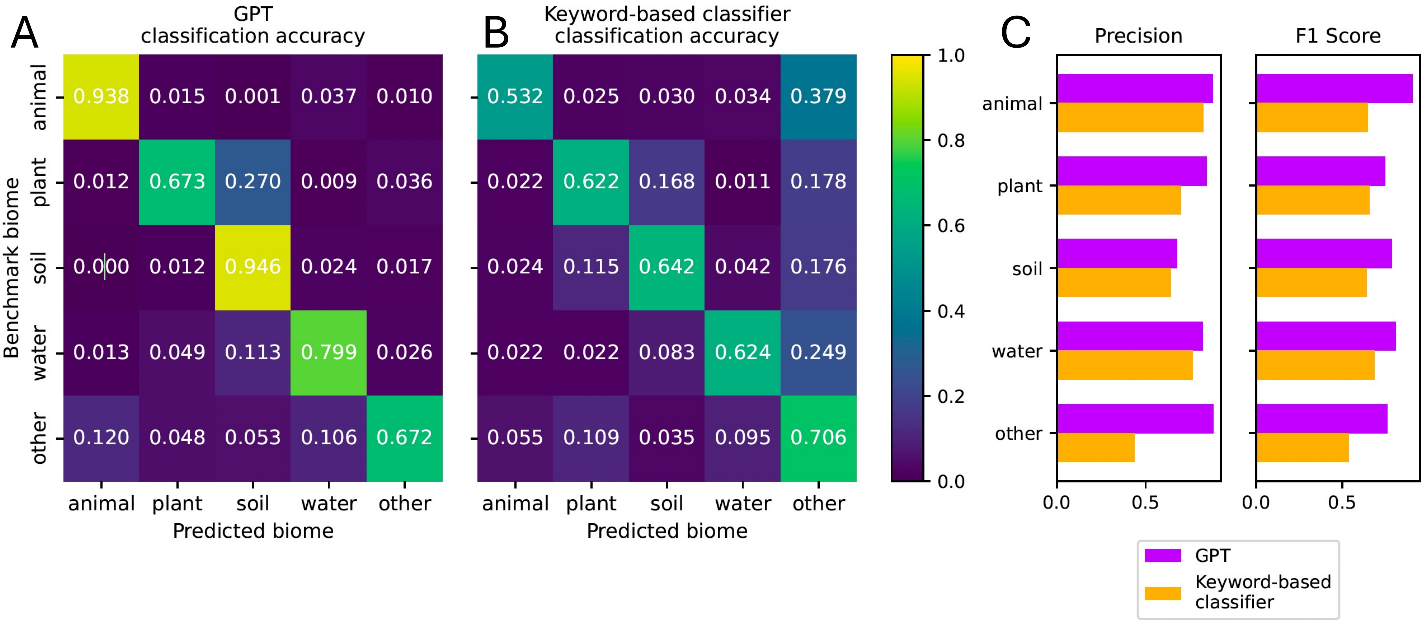

Figure 3. Performance comparison of GPT requests with different chunking sizes. A) Accuracy percentages for biome prediction are shown, reflecting both exact matches and lenient matches between the GPT-generated output and the curator-assigned biomes. The similarity for sub-biome prediction is represented through average cosine similarity. Chunk size (2000 up to 6000) refers to the number of tokens within a single request *i.e.*, chunk. The label "chunk_no" refers to no chunking, where metadata from a single sample is sent in a single request. B) P-values (top of each cell) and adjusted p-values (bottom of each cell) of the performance comparisons are displayed. Cells shaded in green represent the statistical significance of biome accuracy comparisons, while those in blue denote the significance of sub-biome similarity comparisons. The color intensity varies according to the p-value significance. McNemar's and paired t-tests were performed for biome and sub-biome prediction comparisons, respectively. Bonferroni correction was applied on p-values.

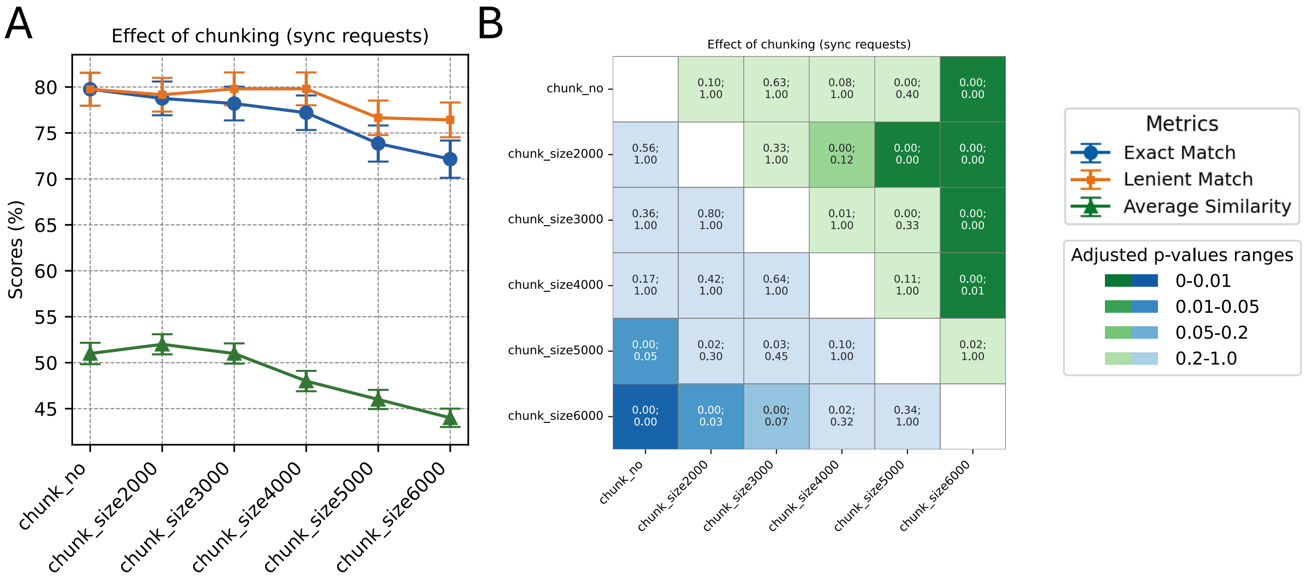

Figure 4. Performance comparison of GPT models. A) Accuracy scores for biome prediction are shown, reflecting both exact matches and lenient matches between the GPT-generated output and the curator-assigned biomes. The similarity for sub-biome prediction is represented through average cosine similarity. B) P-values (top of each cell) and adjusted p-values (bottom of each cell) of the performance comparisons are displayed. Cells shaded in green represent the statistical significance of biome accuracy comparisons, while those in blue denote the significance of sub-biome similarity comparisons. The color intensity varies according to significance. Each run had a replicate (suffix "rep"). McNemar's and paired t-tests were performed for biome and sub-biome prediction comparisons, respectively. Bonferroni correction was applied on p-values.

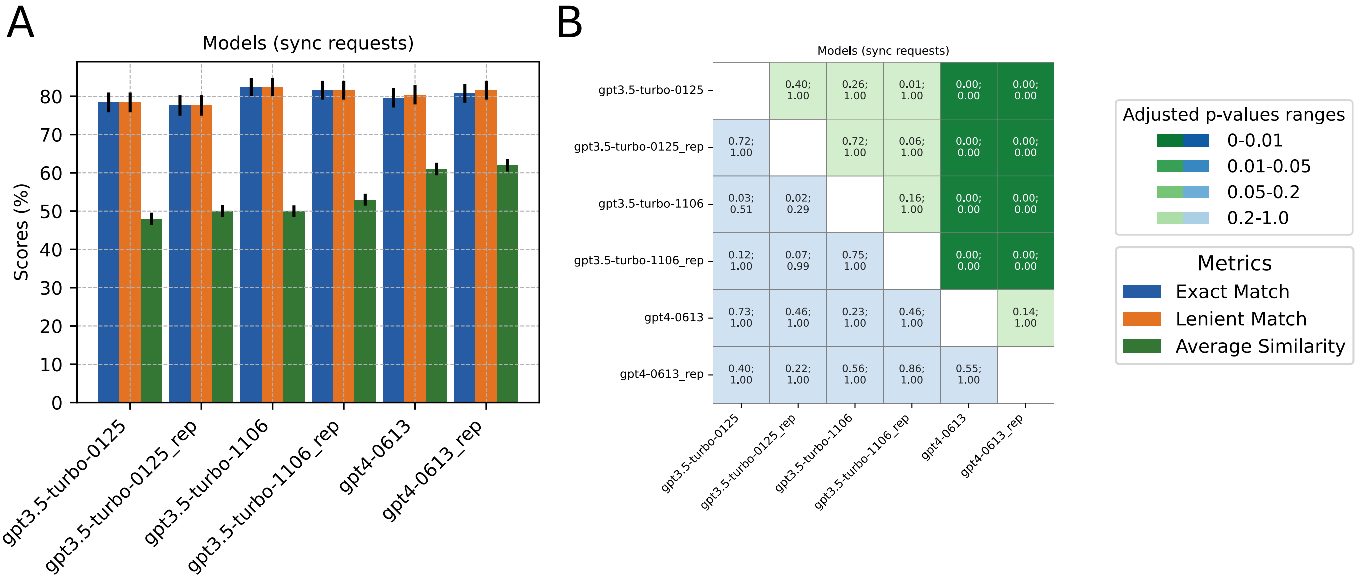

Figure 5. Performance comparison of *GPT-3.5-turbo-1106* using either in-line or json output format. A) Accuracy scores for biome prediction are shown, reflecting both exact matches and lenient matches between the GPT-generated output and the curator-assigned biomes. The similarity for sub-biome prediction is represented through average cosine similarity. B) P-values (top of each cell) and adjusted p-values (bottom of each cell) of the performance comparisons are displayed. Cells shaded in green represent the statistical significance of biome accuracy comparisons, while those in blue denote the significance of sub-biome similarity comparisons. The color intensity varies according to the p-value significance. McNemar's and paired t-tests were performed for biome and sub-biome prediction comparisons, respectively. Bonferroni correction was applied on p-values.

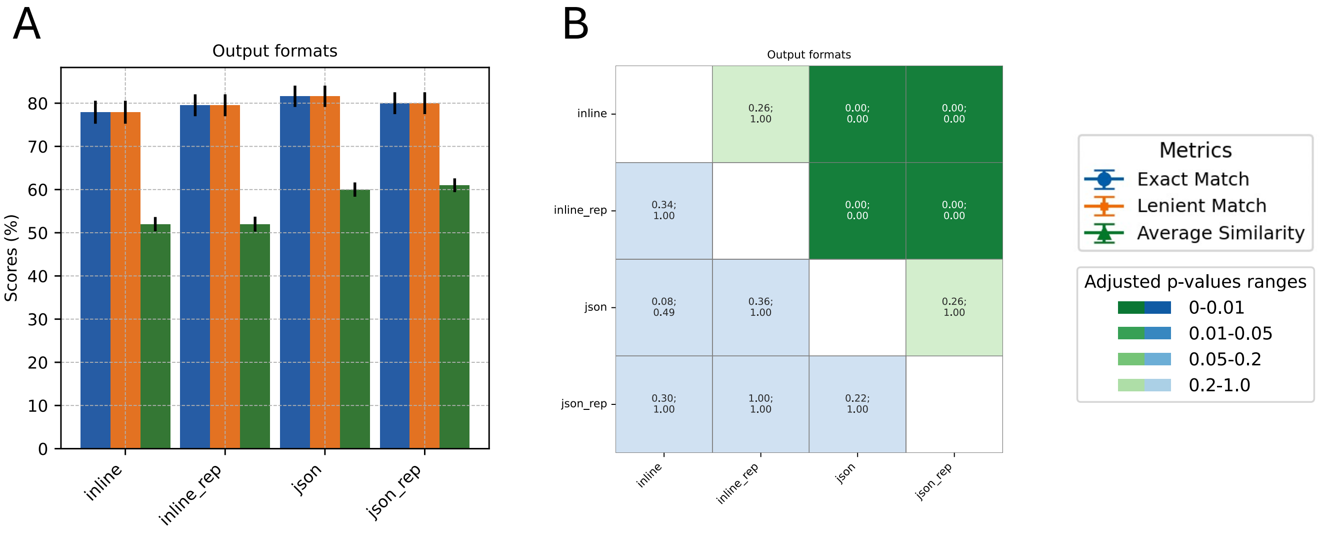

Figure 6. Performance comparison of *GPT-3.5-turbo-1106* when tweaking frequency penalty. A) Accuracy scores for biome prediction are shown, reflecting both exact matches and lenient matches between the GPT-generated output and the curator-assigned biomes. The similarity for sub-biome prediction is represented through average cosine similarity. B) P-values (top of each cell) and adjusted p-values (bottom of each cell) of the performance comparisons are displayed. Cells shaded in green represent the statistical significance of biome accuracy comparisons, while those in blue denote the significance of sub-biome similarity comparisons. The color intensity varies according to the p-value significance. For an assessment of all other creativity parameters see Supplementary Figure 3. McNemar's and paired t-tests were performed for biome and sub-biome prediction comparisons, respectively. Bonferroni correction was applied on p-values.

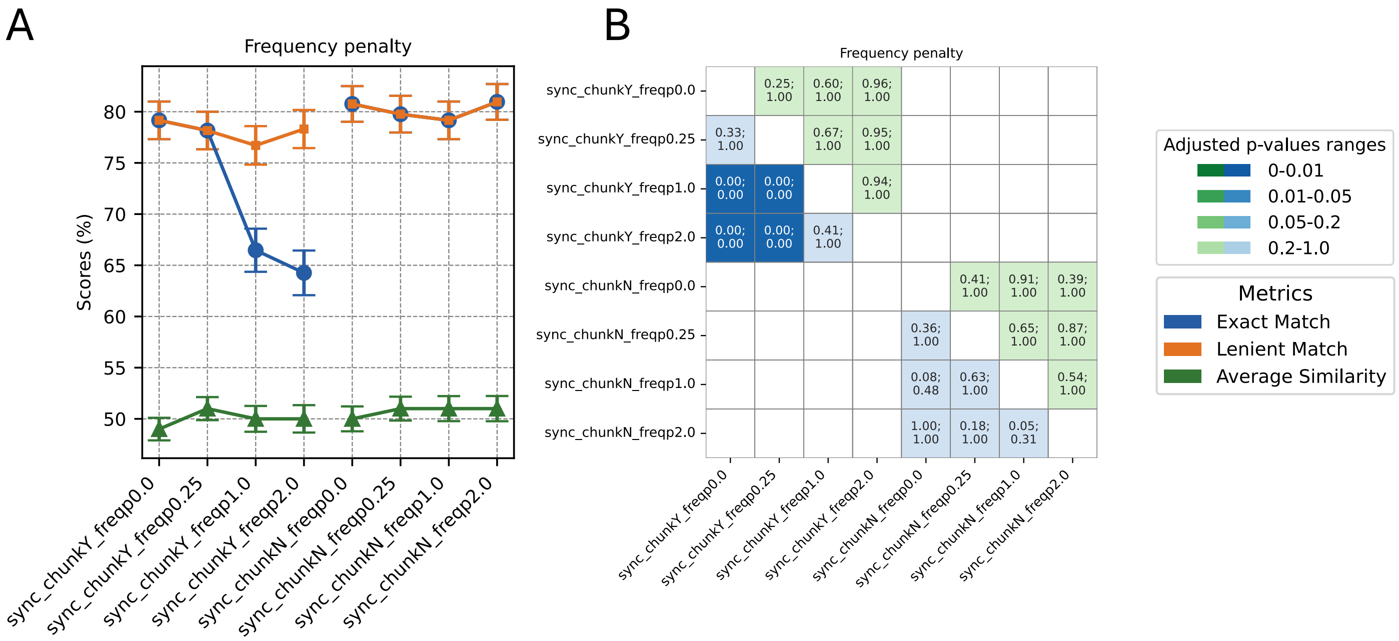

Figure 7. Global distribution of microbiome samples and geographic annotation consistency. A heatmap (blue–green–yellow–red gradient) depicts the global density of a subset of 200,000 microbiome samples (from a pool of 990,172), with warmer colors indicating regions of higher sampling intensity. Overlaid circular clusters mark samples where GPT-inferred geographic locations did not match coordinates extracted from the metadata. Cluster color indicates the number of mismatched samples (green = few, yellow = moderate, red = many). Together, these layers highlight both the uneven geographic distribution of microbiome sampling and the spatial patterns of metadata inconsistencies. The interactive figure can be downloaded from here.

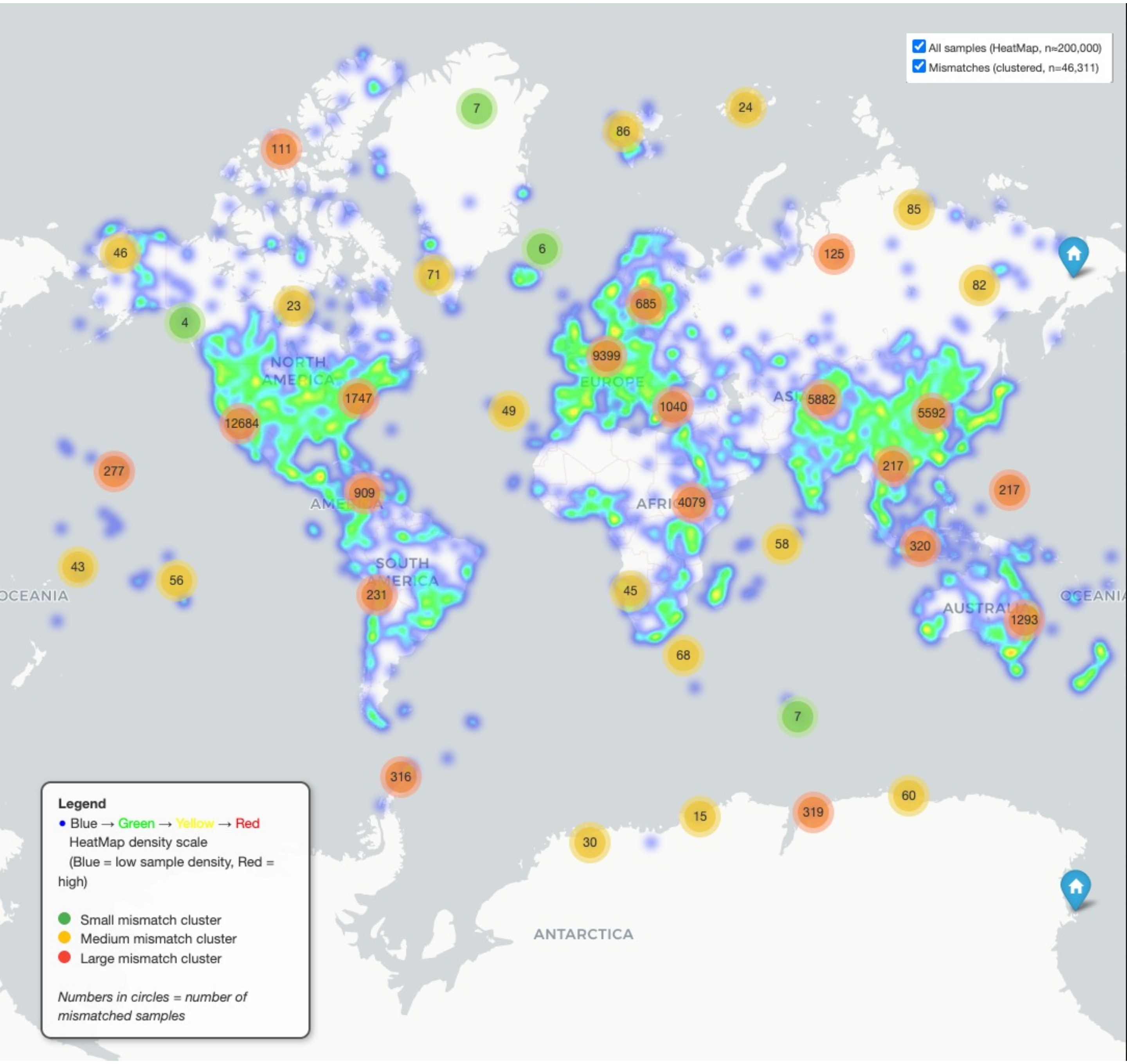

Figure 8. Comparison of Large Language Models (LLMs) and embedding models for biome and sub-biome classification performance. **A**) Accuracy of biome predictions across models, shown as both exact and lenient matches between LLM-generated outputs and curator-assigned biome annotations. Lowercase letters (*a*–*c*) above points indicate results of *post hoc* groupings: models that share the same letter (*e.g.*, all marked "a") do not differ significantly, whereas models with different letters (*e.g.*, "a" *vs*. "b" or "c") show statistically significant differences in accuracy (McNemar's test, Bonferroni-adjusted $p < 0.05$). B) Average cosine similarity between sub-biome annotations generated by the LLMs and converted into embeddings using four different embedding models. Statistical significance for biome predictions was assessed using McNemar's test, and for sub-biome similarities using paired t-tests. Bonferroni correction was applied to adjust p-values for multiple comparisons.

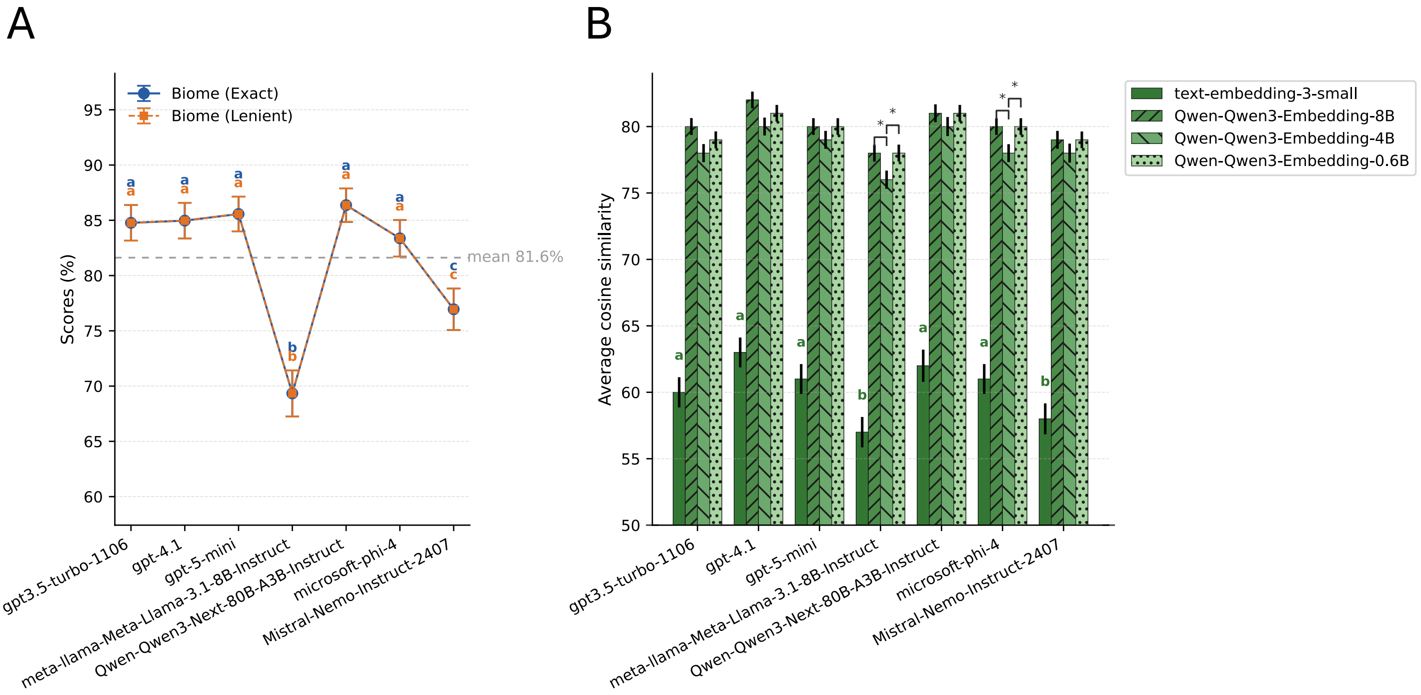

Supplementary Figure 1. Examples of heterogeneous and semantically inconsistent metadata records illustrating challenges for automated parsing: metadata fields containing embedded taxonomic tables and verbose narrative text within descriptive fields (top), redundant or conflicting information distributed across multiple metadata fields (middle), inconsistent formatting, mixed delimiters, and variable completeness across metadata attributes (bottom).

```
>ERS2912775
sample_alias=SAMEA5128390
sample_TITLE=CopperEvo1
sample_TAXON_ID=702656
sample_SCIENTIFIC_NAME=compost metagenome
sample_Alias=CopperEvo1
sample_Description=Sequencing isolates from compost community evolved in presence and absence of copper
sample_ENA checklist=ERC000011
sample_INSDC center alias=UNIVERSITY OF EXETER
sample_INSDC center name=University of Exeter
sample_INSDC first public=2018-12-10T17:02:58Z
sample_INSDC last update=2018-11-23T15:21:40Z
sample_INSDC status=public
sample_SRA accession=ERS2912775
sample_Sample Name=ERS2912775
sample_Title=CopperEvo
study=ERP112283
study_STUDY_TITLE=Compost community evolved with and without copper present
study_STUDY_ABSTRACT=6 compost communities each were evolved in the presence and absence of copper and presence and absence of Pseudomonas fluorescence. Isolates from
both treatments were sequenced and tested for siderophores and plasmid uptake.   Identifiers for which sequences belong to which community and treatment can be found b
elow:  Community Cu Social ID Code Family Genus 1 0 0 1 NMC1_1 Pseudomonadaceae Pseudomonas 1 0 0 2 NMC1_2 Xanthomonadaceae Stenotrophomonas 1 0 0 3 NMC1_3 Xanthomonad
aceae Stenotrophomonas 1 0 0 4 NMC1_4 Bacillaceae_1 Bacillus 1 0 0 5 NMC1_5 Alcaligenaceae Pusillimonas 1 0 0 6 NMC1_6 Microbacteriaceae Microbacterium 1 0 0 7 NMC1_7
Micrococcaceae Arthrobacter 1 0 0 8 NMC1_8   1 0 0 9 NMC1_9 Pseudomonadaceae Pseudomonas 1 0 0 10 NMC1_10 Burkholderiaceae Cupriavidus 1 0 0 11 NMC1_11 Sphingobacteria
ceae unknown Sphingobacteriaceae 1 0 0 12 NMC1_12 Pseudomonadaceae Pseudomonas 1 0 0 13 NMC1_13 Pseudomonadaceae Pseudomonas 1 0 0 14 NMC1_14 Xanthomonadaceae Stenotro
phomonas 1 0 0 15 NMC1_15 Rhizobiaceae unknown Rhizobiaceae 1 0 0 16 NMC1_16 Microbacteriaceae Microbacterium 1 0 0 17 NMC1_17 Pseudomonadaceae Pseudomonas 1 0 0 18 NM
C1_18 Comamonadaceae Acidovorax 1 0 0 19 NMC1_19 Cellulomonadaceae Oerskovia 1 0 0 20 NMC1_20 Alcaligenaceae Achromobacter 1 0 0 21 NMC1_21 Comamonadaceae Variovorax 1
 0 0 22 NMC1_22 Micrococcaceae Arthrobacter 1 0 0 23 NMC1_23 Microbacteriaceae Microbacterium 1 0 0 24 NMC1_24 Burkholderiaceae Cupriavidus 1 0 1 1 NMC1B_1 Nocardiacea
e Rhodococcus 1 0 1 2 NMC1B_2 Alcaligenaceae Achromobacter 1 0 1 3 NMC1B_3 Micrococcaceae Arthrobacter 1 0 1 4 NMC1B_4 Burkholderiaceae Cupriavidus 1 0 1 5 NMC1B_5 Bac
illaceae_1 Bacillus 1 0 1 6 NMC1B_6   1 0 1 7 NMC1B_7   1 0 1 8 NMC1B_8 Comamonadaceae Variovorax 1 0 1 9 NMC1B_9 Pseudomonadaceae Pseudomonas 1 0 1 10 NMC1B_10 Comamo
nadaceae Variovorax 1 0 1 11 NMC1B_11 Microbacteriaceae Microbacterium 1 0 1 12 NMC1B_12 Nocardiaceae Rhodococcus 1 0 1 13 NMC1B_13 Microbacteriaceae Microbacterium 1
0 1 14 NMC1B_14 Hyphomicrobiaceae Devosia 1 0 1 15 NMC1B_15 Comamonadaceae Variovorax 1 0 1 16 NMC1B_16 Hyphomicrobiaceae Devosia 1 0 1 17 NMC1B_17 Nocardiaceae Rhodoc
occus 1 0 1 18 NMC1B_18 Brucellaceae Ochrobactrum 1 0 1 19 NMC1B_19 Alcaligenaceae Candidimonas 1 0 1 20 NMC1B_20 Microbacteriaceae Microbacterium 1 0 1 21 NMC1B_21
1 0 1 22 NMC1B_22 Alcaligenaceae Pusillimonas 1 0 1 23 NMC1B_23 Micrococcaceae Arthrobacter 1 0 1 24 NMC1B_24 Alcaligenaceae Bordetella 1 1 0 1 NMC1x_1 Burkholderiacea
e Cupriavidus 1 1 0 2 NMC1x_2 Caulobacteraceae Brevundimonas 1 1 0 3 NMC1x_3 Comamonadaceae Variovorax 1 1 0 4 NMC1x_4 Burkholderiaceae Cupriavidus 1 1 0 5 NMC1x_5 Mic
rococcaceae Arthrobacter 1 1 0 6 NMC1x_6 Microbacteriaceae Microbacterium 1 1 0 7 NMC1x_7   1 1 0 8 NMC1x_8 Burkholderiaceae Cupriavidus 1 1 0 9 NMC1x_9 Micrococcaceae
 Arthrobacter 1 1 0 10 NMC1x_10 Micrococcaceae Arthrobacter 1 1 0 11 NMC1x_11   1 1 0 12 NMC1x_12   1 1 0 13 NMC1x_13 Bacillaceae_1 Bacillus 1 1 0 14 NMC1x_14 Micrococ
caceae Arthrobacter 1 1 0 15 NMC1x_15 Pseudomonadaceae Pseudomonas 1 1 0 16 NMC1x_16 Burkholderiaceae Cupriavidus 1 1 0 17 NMC1x_17 Micrococcaceae Arthrobacter 1 1 0 1
8 NMC1x_18   1 1 0 19 NMC1x_19 Burkholderiaceae Cupriavidus 1 1 0 20 NMC1x_20 Burkholderiaceae Cupriavidus 1 1 0 21 NMC1x_21   1 1 0 22 NMC1x_22   1 1 0 23 NMC1x_23 Ba
cillaceae_1 Bacillus 1 1 0 24 NMC1x_24   1 1 1 1 NMC1xB_1 Burkholderiaceae Cupriavidus 1 1 1 2 NMC1xB_2   1 1 1 3 NMC1xB_3   1 1 1 4 NMC1xB_4 Burkholderiaceae Cupriavi
dus 1 1 1 5 NMC1xB_5 Burkholderiaceae Cupriavidus 1 1 1 6 NMC1xB_6 Burkholderiaceae Cupriavidus 1 1 1 7 NMC1xB_7 Nocardiaceae Rhodococcus 1 1 1 8 NMC1xB_8 Burkholderia
ceae Cupriavidus 1 1 1 9 NMC1xB_9 Micrococcaceae Arthrobacter 1 1 1 10 NMC1xB_10 Burkholderiaceae Cupriavidus 1 1 1 11 NMC1xB_11   1 1 1 12 NMC1xB_12   1 1 1 13 NMC1xB
_13   1 1 1 14 NMC1xB_14 Burkholderiaceae Cupriavidus 1 1 1 15 NMC1xB_15   1 1 1 16 NMC1xB_16   1 1 1 17 NMC1xB_17 Burkholderiaceae Cupriavidus 1 1 1 18 NMC1xB_18 Micr
ococcaceae Arthrobacter 1 1 1 19 NMC1xB_19   1 1 1 20 NMC1xB_20 Bacillaceae_1 Bacillus 1 1 1 21 NMC1xB_21 Burkholderiaceae Cupriavidus 1 1 1 22 NMC1xB_22 Burkholderiac
eae Cupriavidus 1 1 1 23 NMC1xB_23 Micrococcaceae Arthrobacter 1 1 1 24 NMC1xB_24   2 0 0 1 NMC2_1 Microbacteriaceae Microbacterium 2 0 0 2 NMC2_2 Rhizobiaceae unknown
 Rhizobiaceae 2 0 0 3 NMC2_3   2 0 0 4 NMC2_4 Micrococcaceae Arthrobacter 2 0 0 5 NMC2_5 Micrococcaceae Arthrobacter 2 0 0 6 NMC2_6 Pseudomonadaceae Pseudomonas 2 0 0
7 NMC2_7   2 0 0 8 NMC2_8 Bacillaceae_1 Bacillus 2 0 0 9 NMC2_9 Bacillaceae_1 Bacillus 2 0 0 10 NMC2_10 Micrococcaceae Arthrobacter 2 0 0 11 NMC2_11   2 0 0 12 NMC2_12
 Pseudomonadaceae Pseudomonas 2 0 0 13 NMC2_13   2 0 0 14 NMC2_14   2 0 0 15 NMC2_15   2 0 0 16 NMC2_16 Micrococcaceae Arthrobacter 2 0 0 17 NMC2_17 Planococcaceae Lys
inibacillus 2 0 0 18 NMC2_18 Pseudomonadaceae Pseudomonas 2 0 0 19 NMC2_19   2 0 0 20 NMC2_20 Micrococcaceae Arthrobacter 2 0 0 21 NMC2_21 Pseudomonadaceae Pseudomonas
 2 0 0 22 NMC2_22 Pseudomonadaceae Pseudomonas 2 0 0 23 NMC2_23   2 0 0 24 NMC2_24 Rhizobiaceae unknown Rhizobiaceae 2 0 1 1 NMC2B_1   2 0 1 2 NMC2B_2 Cellulomonadacea
e Oerskovia 2 0 1 3 NMC2B_3 Microbacteriaceae Microbacterium 2 0 1 4 NMC2B_4 Microbacteriaceae Microbacterium 2 0 1 5 NMC2B_5 Microbacteriaceae Microbacterium 2 0 1 6
NMC2B_6 Xanthomonadaceae Stenotrophomonas 2 0 1 7 NMC2B_7 Planococcaceae Lysinibacillus 2 0 1 8 NMC2B_8 Microbacteriaceae Microbacterium 2 0 1 9 NMC2B_9 Xanthomonadace
ae Stenotrophomonas 2 0 1 10 NMC2B_10 Microbacteriaceae Microbacterium 2 0 1 11 NMC2B_11 Microbacteriaceae Microbacterium 2 0 1 12 NMC2B_12 Planococcaceae Lysinibacill
us 2 0 1 13 NMC2B_13 Xanthomonadaceae Stenotrophomonas 2 0 1 14 NMC2B_14 Microbacteriaceae Microbacterium 2 0 1 15 NMC2B_15 Microbacteriaceae Microbacterium 2 0 1 16 N
MC2B_16   2 0 1 17 NMC2B_17 Xanthomonadaceae Stenotrophomonas 2 0 1 18 NMC2B_18 Microbacteriaceae Microbacterium 2 0 1 19 NMC2B_19 Microbacteriaceae Microbacterium 2 0
 1 20 NMC2B_20 Xanthomonadaceae Stenotrophomonas 2 0 1 21 NMC2B_21 Microbacteriaceae Microbacterium 2 0 1 22 NMC2B_22   2 0 1 23 NMC2B_23 Planococcaceae Lysinibacillus
```

```
>SRS4101975
sample_alias=QQYLD3
sample_TAXON_ID=1616788
sample_SCIENTIFIC_NAME=Paenibacillus bovis
sample_strain=BD3526
sample_collection_date=03-Nov-2018
sample_env_broad_scale=mangrove biome
sample_env_local_scale=shoreline
sample_env_medium=estuarine water
sample_geo_loc_name=China:Shanghai
sample_host=rat
sample_isol_growth_condt=The sample was isolated from GK rat of which treated with BD3526
sample_lat_lon=39.92 N 121.12 E
sample_num_replicons=7
sample_ref_biomaterial=Yes
sample_type-material=type strain of Paenibacillus bovis
sample_BioSampleModel=MIGS.ba
sample_BioSampleModel=MIGS/MIMS/MIMARKS.human-gut
study=SRP172190
study_STUDY_TITLE=Microbiota of which treated with BD3526 fermentations. Metagenome
study_STUDY_ABSTRACT=The sample was acquired from GK rat of which treated with BD3526. Metagenome
```

```
>SRS2002454
sample_alias=070.C.D3.10
sample_TITLE=Sample 070 (ID: 070_C_D3_10), Day3, Carbaryl treated, 16s V4 region
sample_TAXON_ID=1169740
sample_SCIENTIFIC_NAME=aquatic metagenome
sample_XREF_LINK=bioproject: 374734)
sample_XREF_LINK=bioproject: 374734)
sample_source_material_id=070
sample_collection_date=29-Oct-2015
sample_env_biome=microcosm
sample_env_feature=indoor
sample_env_material=water
sample_geo_loc_name=USA: 1902 Griffith Dr., Champaign, IL
sample_lat_lon=40.0926 N 88.2427 W
sample_chem_administration=Carbaryl
sample_isolation_source=northern red oak water infusion
sample_perturbation=pesticide administration
sample_TreatmentCode=C
sample_TreatDetail=Carbaryl
sample_TreatmentDuration=Day3
sample_BioSampleModel=MIMARKS.survey
sample_BioSampleModel=MIGS/MIMS/MIMARKS.miscellaneous
study=SRP100721
study_STUDY_TITLE=Effect of pesticides on microbiome in mosquito container habitat
study_STUDY_ABSTRACT=In order to examine the effects of two herbicides (atrazine, glyphosate) and three insecticides (malathion, carbaryl, permethrin) on microbial communities of a
typical mosquito aquatic habitat, MiSeq sequencing of the V4 region of the 16S rRNA gene was used to characterize the microbial communities of indoor microcosms that were either exp
osed to each pesticide alone, a mix of herbicides, a mix of insecticides, or a mix of all five insecticides.
```

Supplementary Figure 2: Pipeline in detail. C1 to C4 denote the containers. Detailed instructions on how to reproduce the workflow using the containers can be found here.

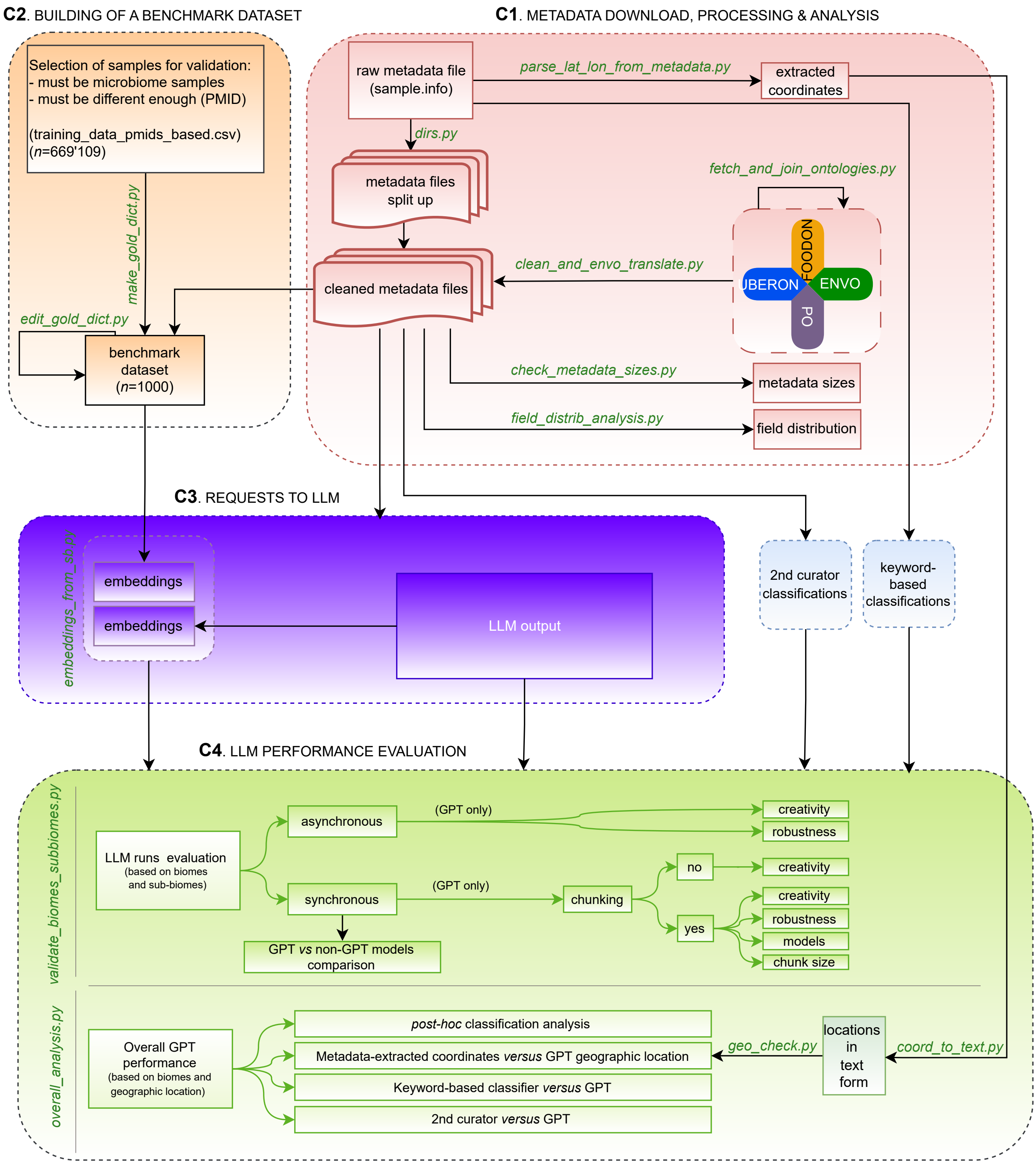

Supplementary Figure 3. Pipeline of synchronous (A) and asynchronous (B) requests. Synchronous and asynchronous pipelines have been used for all OpenAI models; whereas requests to all other LLMs were done synchronously.

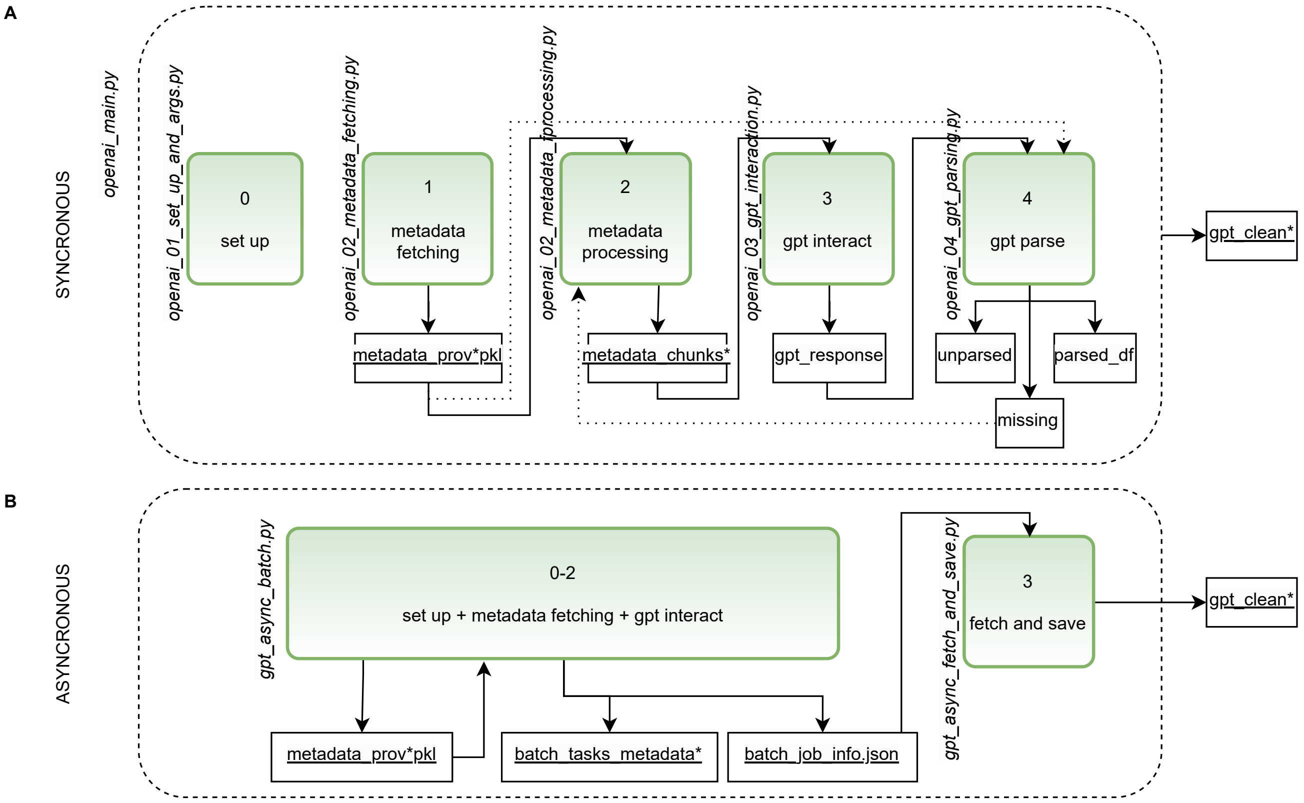

Supplementary Figure 4. Misclassifications by GPT.

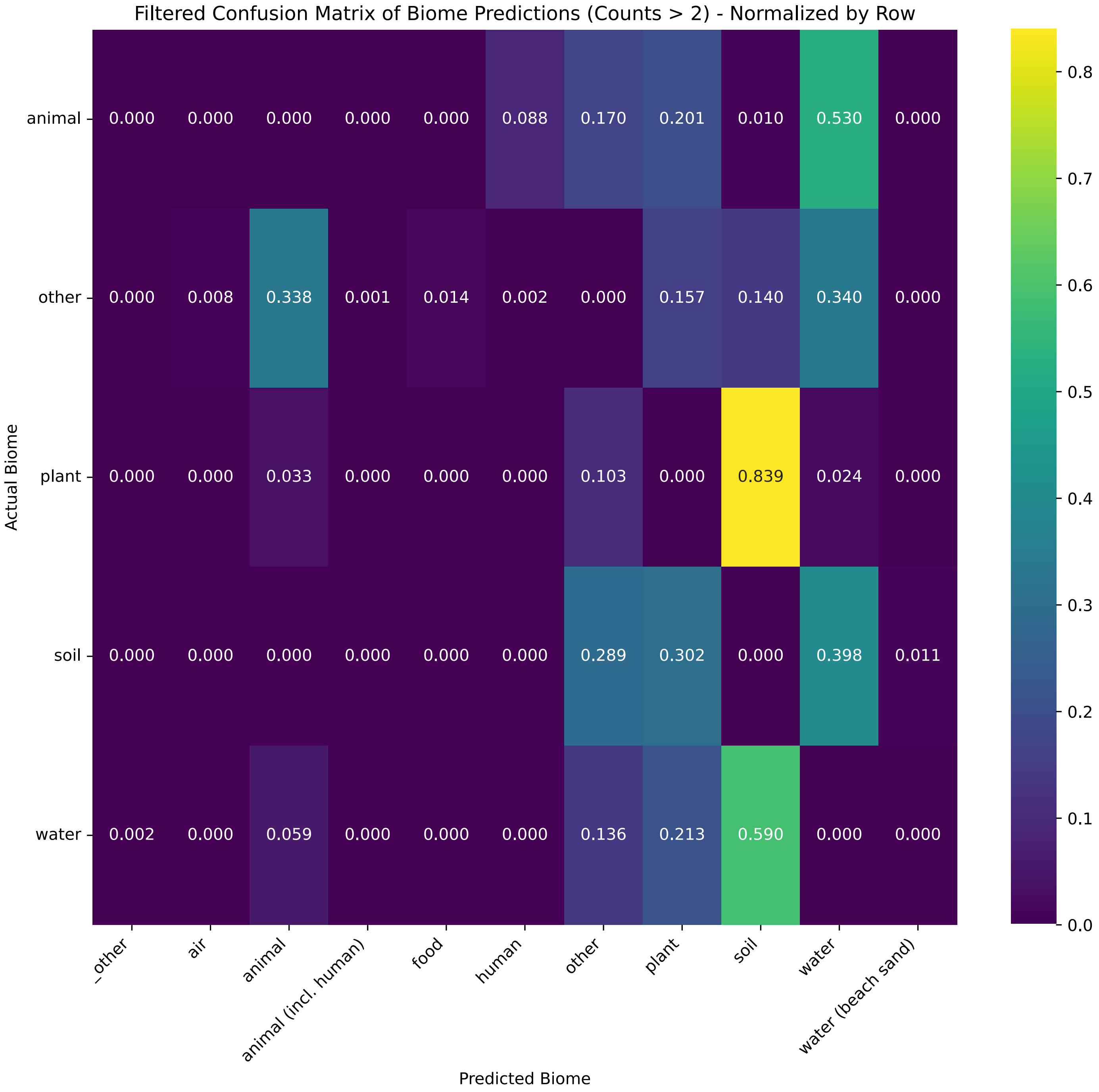

Supplementary Figure 5. Distribution of sample misclassifications by GPT.

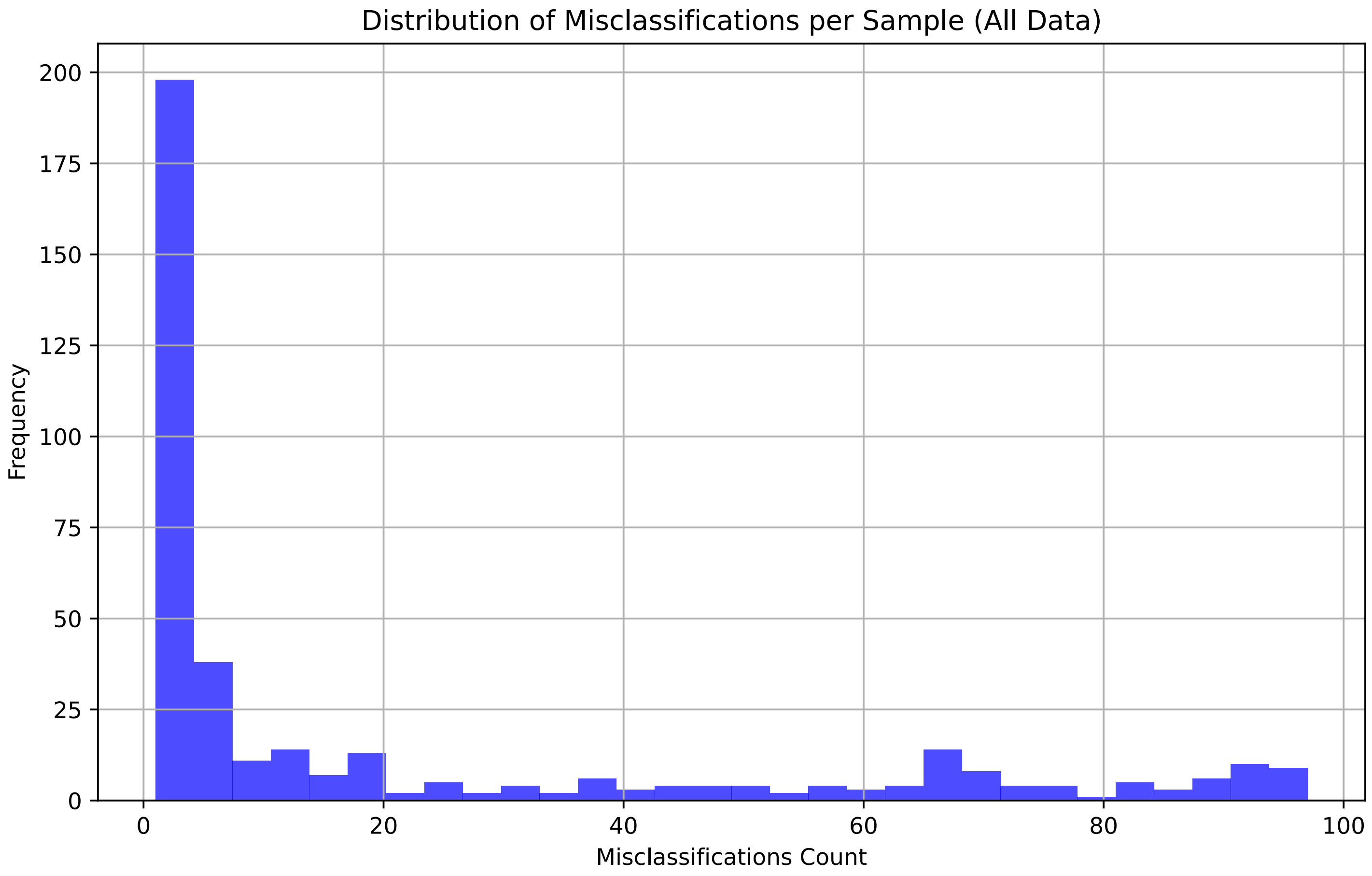

Supplementary Figure 6. Performance comparison of GPT when tweaking creativity parameters: temperature (temp), nucleus sampling (topp), frequency penalty (freqp) and presence penalty (presp). A-D) Accuracy scores for biome prediction are shown, reflecting both exact matches (blue line) and lenient (orange line) matches between the GPT-generated output and the curator-assigned biomes. The similarity for sub-biome prediction is represented through average (cosine) similarity (green line). E-H) P-values (top of each cell) and adjusted p-values (bottom of each cell) of the performance comparisons are displayed. Cells shaded in green represent the statistical significance of biome accuracy comparisons, while those in blue denote the significance of sub-biome similarity comparisons. The color intensity varies according to the p-value significance. McNemar's and paired t-tests were performed for biome and sub-biome prediction comparisons, respectively. Bonferroni correction was applied on p-values.

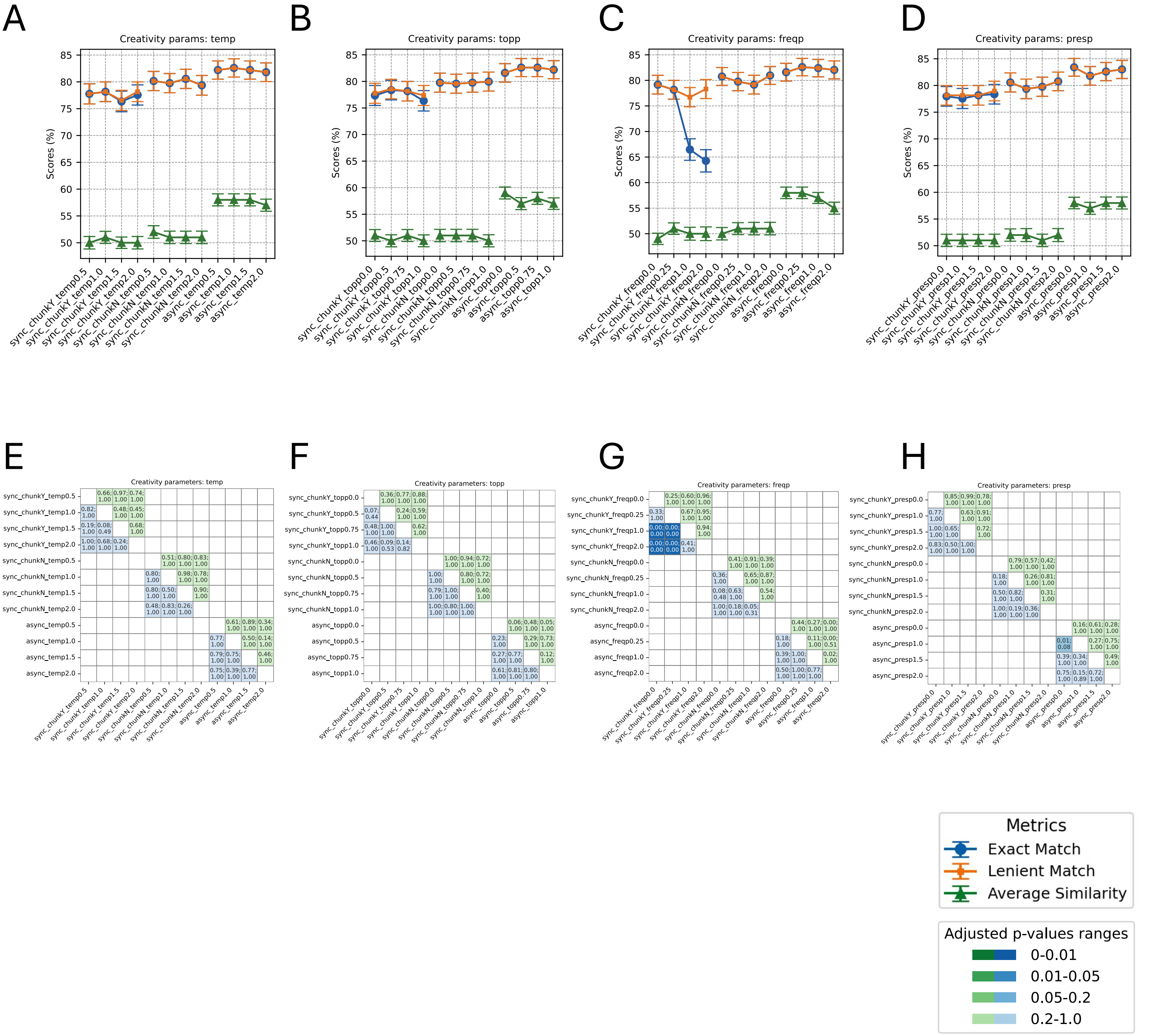

Supplementary Figure 7. Performance reproducibility. A-C) Accuracy scores for biome prediction are shown, reflecting both exact matches and lenient matches between the GPT-generated output and the curator-assigned biomes. The similarity for sub-biome prediction is represented through average (cosine) similarity. D-F) P-values (top of each cell) and adjusted p-values (bottom of each cell) of the performance comparisons are displayed. Cells shaded in green represent the statistical significance of biome accuracy comparisons, while those in blue denote the significance of sub-biome similarity comparisons. The color intensity varies according to the p-value significance. McNemar's and paired t-tests were performed for biome and sub-biome prediction comparisons, respectively, when the sample groups being compared were the same (same 'rs' *i.e.*, random seed). When comparing different sample groups, independent t-tests were applied. Bonferroni correction was applied on p-values.

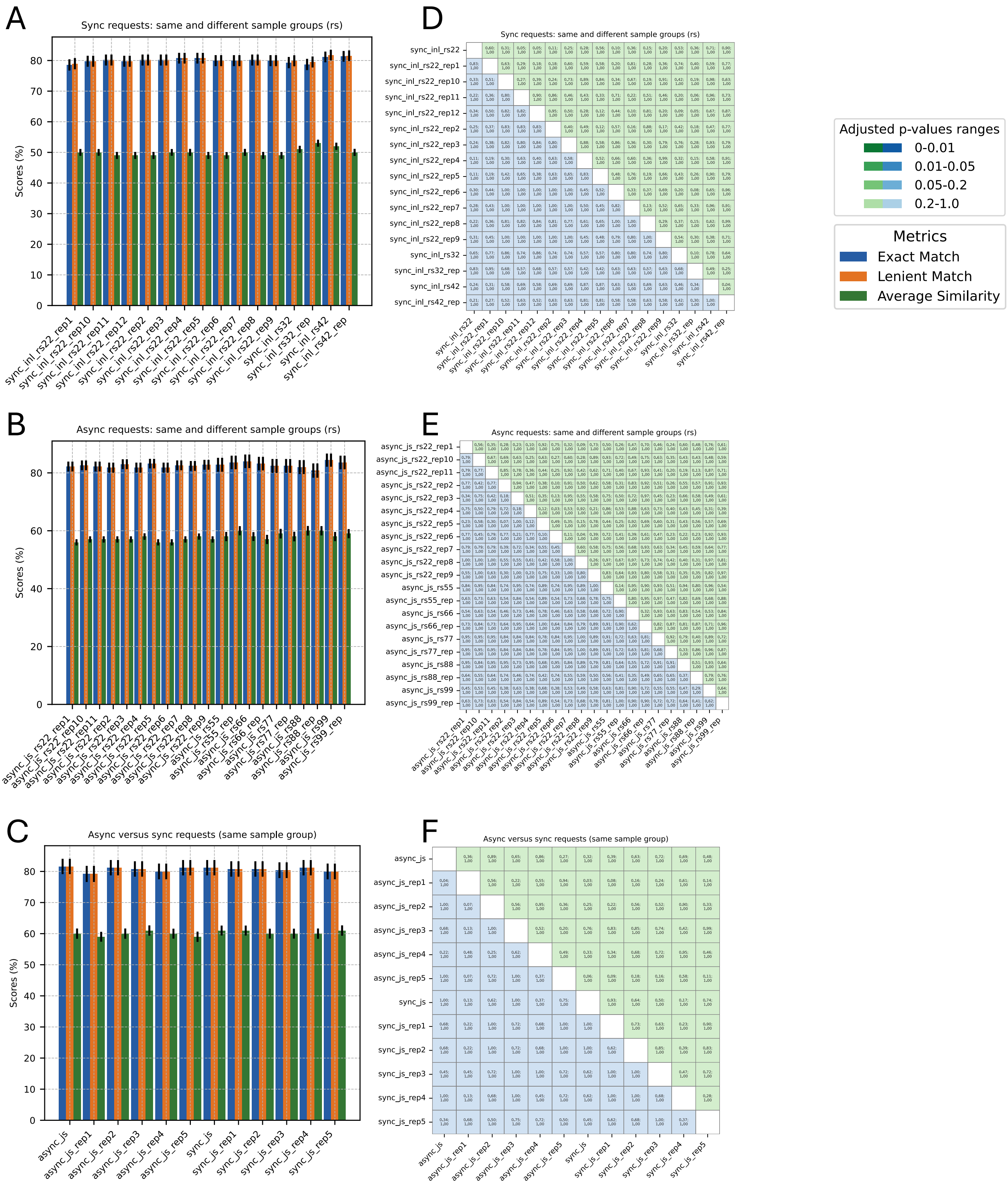

Supplementary Figure 8. Spatial distribution of samples showing GPT–metadata location mismatches. Each point marks a sample for which GPT-inferred location and metadata-derived coordinates differed. Colors denote increasing distance between the two locations (blue ≤ 100 km; green 100–500 km; yellow 500–1,000 km; orange 1,000–4,000 km; red > 4,000 km). Circle size reflects the number of overlapping samples. In the interactive figure, clicking on any point displays detailed information, including the original metadata-derived location, the GPT-inferred name, the geographic distance between them, and the number of samples represented. The map illustrates the geographic extent of annotation inconsistencies and identifies regions with frequent long-distance mismatches. The map displays all mismatches (n=46,311) from a pool of 990,172 samples.

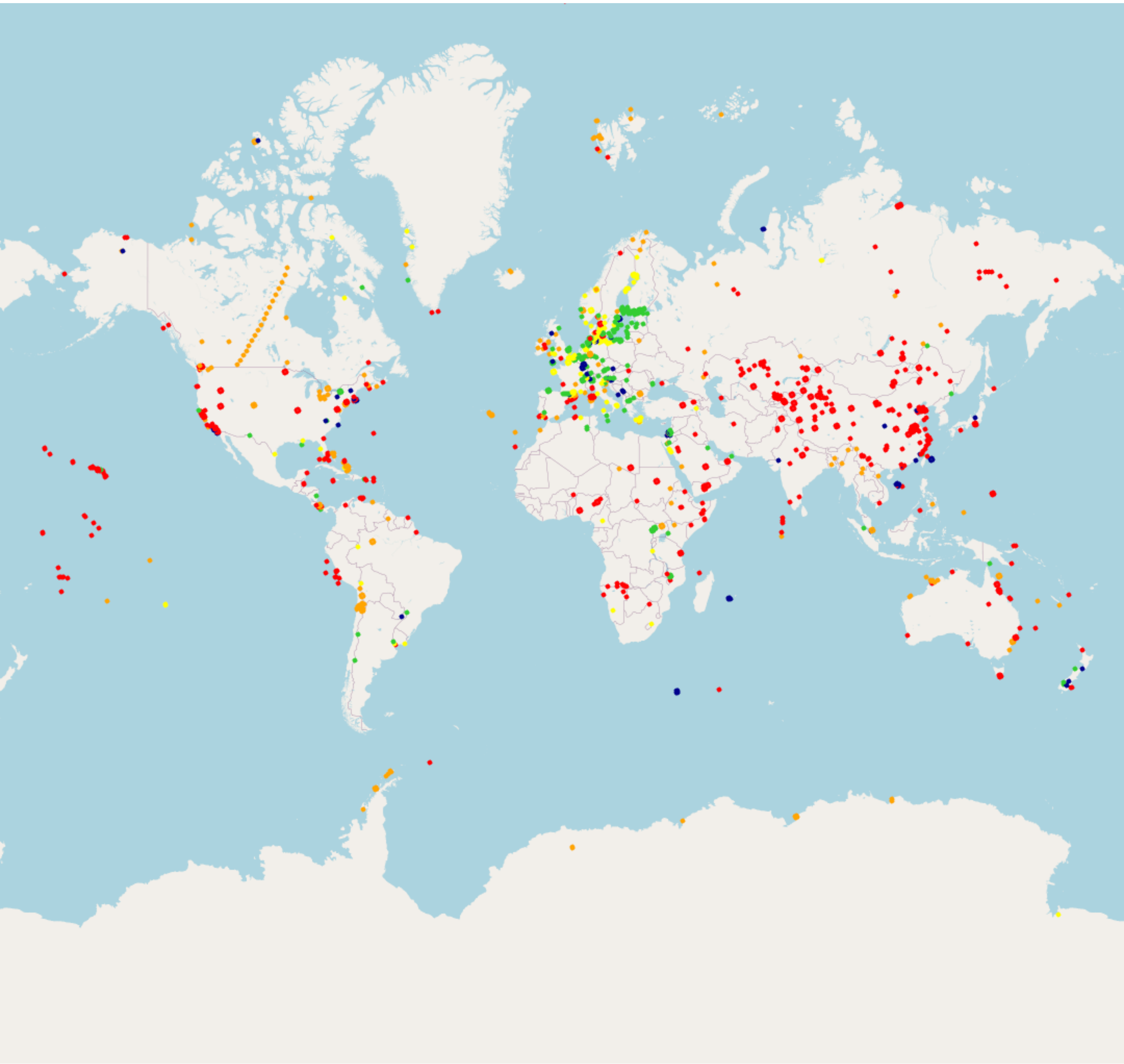

Supplementary Figure 9. Metadata field distribution. Frequency of metadata fields matching benchmark sub-biomes by biome category. Fields with less than 20 partial matches are filtered out.

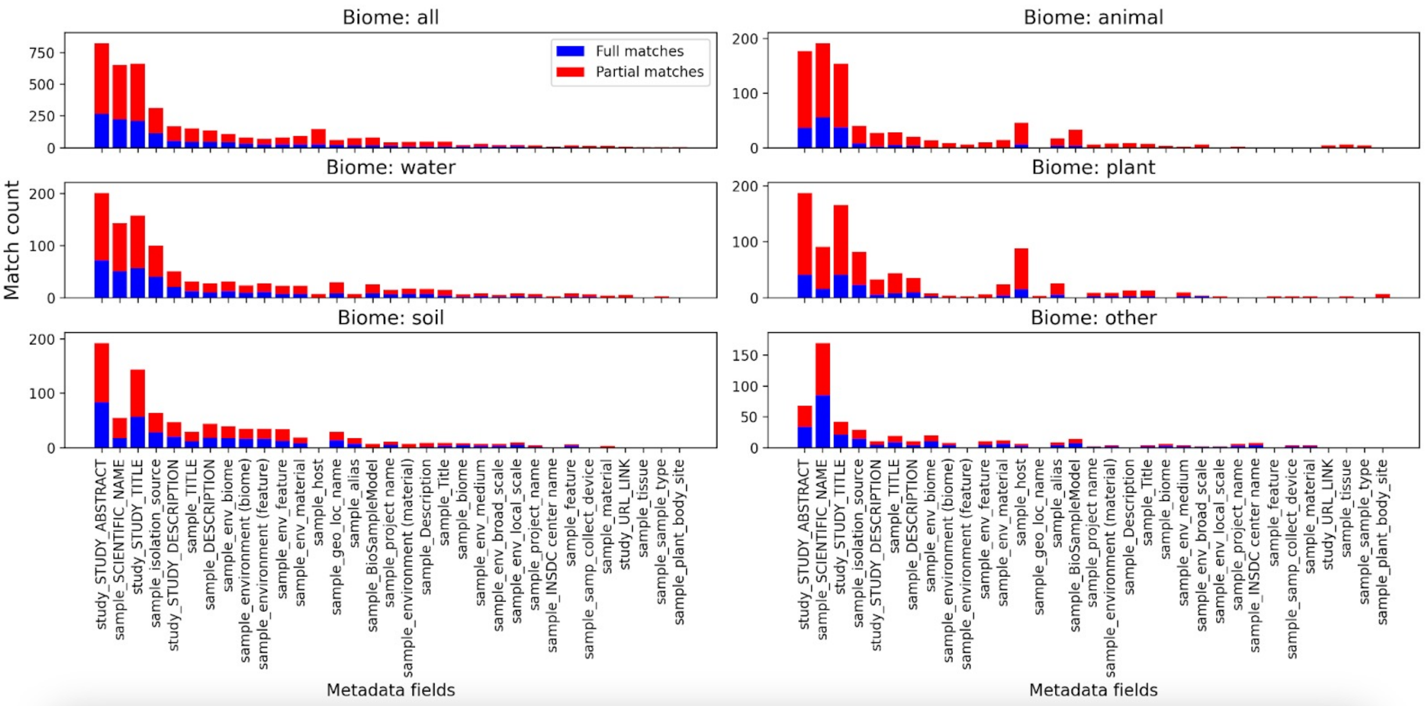

Supplement: giag015_Supplemental_File [file giag015_supplemental_file.pdf]
