# Supplementary material for: Enhanced semantic classification of microbiome sample origins using large language models (LLMs)
Source: Gigascience. 2026 Feb 12;15:giag015. doi: 10.1093/gigascience/giag015 (PMC13042274; doi:10.1093/gigascience/giag015)
Supplement: giag015_GIGA-D-25-00316_revision_1 [file giag015_giga-d-25-00316_revision_1.pdf]

GigaScience

Enhanced semantic classification of microbiome sample origins using Large Language Models (LLMs)

--Manuscript Draft--

**Manuscript Number:**	GIGA-D-25-00316R1
**Full Title:**	Enhanced semantic classification of microbiome sample origins using Large Language Models (LLMs)
**Article Type:**	Research
**Funding Information:**	NCCR Microbiomes (310030_192567) — PhD Daniela Gaio, PhD Christian von Mering<br>SNSF (310030_192569) — MSc Eugenio Perez-Molphe-Montoya, MSc Nicolas Näpflin, PhD Lukas Malfertheiner, MSc Matteo Eustachio Peluso<br>NCCR Microbiomes (310030_192567) — PhD David Patsch
**Abstract:**	Over the past decade, central sequence repositories have expanded significantly in size. This vast accumulation of data holds value and enables further studies, provided that the data entries are well annotated. However, the submitter-provided metadata of sequencing records can be of heterogeneous quality, presenting significant challenges for re-use. Here, we test to what extent large language models (LLMs) can be used to cost-effectively automate the re-annotation of sequencing records against a simplified classification scheme of broad ecological environments with relevance to microbiome studies, without fine-tuning. This effort directly contributes to improving the FAIRness—Findability, Accessibility, Interoperability, and Reusability—of microbiome sequencing metadata, thereby enhancing their “AI readiness” for downstream computational analyses.We focused on sequencing samples taken from the environment, for which metadata is important. We employed OpenAI Generative Pre-trained Transformer (GPT) models, and assessed scalability, time- and cost-effectiveness, as well as performance against a diverse, hand-curated benchmark with 1,000 examples, that span a wide range of complexity in metadata interpretation. Annotation performance markedly outperformed that of a baseline, manually curated, non-ML keyword-based approach. Changing models (or model parameters) has only minor effects on performance, but prompts need to be carefully designed to match the task. Furthermore, when we compared proprietary OpenAI models with open-weight alternatives (e.g., Qwen, meta-Llama, and microsoft-phi-4), we found comparable accuracy for both biome and sub-biome classification, indicating that open-weight architectures can match the performance of proprietary models for large-scale ecological metadata re-annotation.We validated the pipeline with 1,000 hand-curated samples, and we applied the optimized pipeline to 2 million sequencing records from the environment, providing coarse-grained yet standardized sample origin annotations covering the globe. Our work demonstrates the effective use of LLMs to simplify and standardize annotation from complex biological metadata.
**Corresponding Author:**	Daniela Gaio, PhD<br>University of Zurich: Universitat Zurich<br>Zurich, - SWITZERLAND
**Corresponding Author Secondary Information:**	
**Corresponding Author's Institution:**	University of Zurich: Universitat Zurich
**Corresponding Author's Secondary Institution:**	
**First Author:**	Daniela Gaio
**First Author Secondary Information:**	

Powered by Editorial Manager® and ProduXion Manager® from Aries Systems Corporation

**Order of Authors:**	Daniela Gaio
	Janko Tackmann
	Eugenio Perez-Molphe-Montoya
	Nicolas Näpflin
	David Patsch
	Lukas Malfertheiner
	Matteo Eustachio Peluso
	Christian von Mering
**Order of Authors Secondary Information:**	
**Response to Reviewers:**	Dear Hongfang Zhang,<br>We would like to thank you and the reviewers for the time and thoughtful feedback provided on our manuscript “Enhanced semantic classification of microbiome sample origins using Large Language Models (LLMs)” (GIGA-D-20-00347).<br>We have carefully considered all comments and substantially revised the manuscript. The main updates are summarized below, while detailed, point-by-point responses follow.<br>The most significant revision concerns reproducibility, as raised by Reviewer 2. During re-examination of our Dockerized scripts, we identified a silent bug in the file-handling logic of validate_biomes_subbiomes.py, which caused output concatenation and ambiguous comparisons between mismatched runs. This issue has now been fully corrected. Container 4 now generates clean outputs at each run, and the Zenodo repository (new DOI created for new version - please see revised manuscript) and documentation have been reorganized to ensure full reproducibility and transparency.<br>The second major revision expands the manuscript’s analytical scope. In response to Reviewer 1, we added results for more recent proprietary models (GPT-4.1, GPT-5-mini) and several open-weight LLMs (e.g.meta-llama/Meta-Llama-3.1-8B-Instruct, Qwen/Qwen3-Next-80B-A3B-Instruct, microsoft-phi-4, and Mistral-Nemo-Instruct-2407). This addition considerably strengthens the study: several open-weight models achieved comparable or superior accuracy at a fraction of the cost, showing that large-scale metadata re-annotation is now feasible without dependence on commercial APIs.<br>Given the importance and technical nature of this new analysis, we have added Matteo Peluso as a co-author. He is an expert in machine learning and contributed substantially to the implementation of the open-weight model experiments introduced in response to Reviewer 1’s comment.<br>We also wish to thank Reviewer 3 whose insightful remarks helped us clarify the manuscript’s broader relevance with respect to data reusability and the FAIR principles. We have strengthened the introduction and discussion to explicitly connect our work to FAIR and improved readability for both technical and non-technical audiences.<br>Additionally, we would like to kindly ask whether it would be possible to include or confirm the ORCID identifiers of two co-authors, Eugenio Perez-Molphe-Montoya and David Patsch, in the revised submission. We are not certain whether their ORCIDs were included in the first submission and would appreciate the opportunity to ensure that all author records are complete and accurate.<br>●Eugenio Perez-Molphe-Montoya: 0009-0002-9592-9455<br>●David Patsch: 0009-0002-9859-091X<br>All links have been updated in the revised manuscript:<br>-Docker image: https://hub.docker.com/r/gaiotransposon/metadmin (directs to the latest)<br>-Interactive map figures: https://zenodo.org/records/17436923 (new doi) and https://zenodo.org/records/17436804. (old doi, new version)<br>-Deposited data: https://doi.org/10.5281/zenodo.17437043<br>We believe these revisions have enhanced the manuscript’s clarity, reproducibility, and long-term relevance. We are grateful for the reviewers’ work, and the constructive feedback we received, which has led to a stronger and more comprehensive paper.<br>Kind regards<br>Daniela Gaio (on behalf of all co-authors)

Powered by Editorial Manager® and ProduXion Manager® from Aries Systems Corporation

##

Response to Reviewers:

First of all, we would like to thank all three reviewers for their valuable input in our manuscript. We believe the comments were on point, and the changes to the manuscript that followed made a significant impact on its quality and clarity.

Reviewer #1: The manuscript presents a carefully executed study using non-finetuned GPT models to classify microbiome sample metadata. It is very well written, and both the analyses and the interpretations are generally sound. I found the evaluation thorough and the presentation clear.

1. The study provides a detailed evaluation of LLM-based metadata curation, clearly advancing over keyword-based approaches. However, it is surprising that recent related studies using LLMs for metadata curation are not cited. For completeness, I suggest including references such as:
- https://doi.org/10.1093/gigascience/giaf070 (disclaimer: I am an author of the paper. You might like the table 3),
- https://doi.org/10.1093/bib/bbad535 ,
- https://doi.org/10.3897/phytokeys.261.158396 ,
- https://pmc.ncbi.nlm.nih.gov/articles/PMC12099408/.

We thank Reviewer 1 for the references! We agree that our cited literature was not fully up to date with the most recent advancements in the field (2024-2025). We have added the references above throughout the text and a few more references.

2. The scale of the processed data is impressive. However, there appears to be a discrepancy: the Zenodo repository file metadata.out contains 2,254,619 accession IDs (presumably the input), while the GPT output files (gpt_clean_output) include only around 1,000 samples (presumably the benchmark dataset), whereas the manuscript states that 3.8 million samples were processed. It would be helpful to clarify these numbers and, if applicable, explain why fewer outputs are provided. I also recommend reorganizing the Zenodo repository so that readers can download individual files rather than the entire large archive.

Indeed, we did not fully clarify these numbers:

Powered by Editorial Manager® and ProduXion Manager® from Aries Systems Corporation

- The manuscript does state the initial pool of 3.8M samples, but that reflects in fact the initial pool, prior to any filtering. 3.8M is the amount of samples that were originally downloaded from NCBI SRA for having relevant metadata keywords such as "metagenomic", "microb*", "bacteria", or "archea". We have now made that clearer. (lines: 157-161)
- The gpt_clean_output* files are the outputs that have been used for validation, so they indeed only contain the sample IDs from the benchmark (n=1000). We have now added throughout that 1000 is the number of samples used for the benchmark. (lines: 24, 33, 118, 211, 393, 653)
- 2,254,619 are the actual sample IDs that have been submitted for the production run. We now explicitly report the output size of the production run (over 2M samples). (lines: 33-34, 119, 473)
- We clarify in the abstract and in the introduction the numbers to be expected from the validation of the pipeline (1000) and from the production run (over 2M). (lines: 24, 34)
- We noticed that the methods section was missing mentioning the number of samples (1000) used for validation. We have now included this. (line: 211)
- Additionally, in our previous version we did not provide the production run output (neither in the manuscript nor on Zenodo). We now mention its availability in various parts in the manuscript (abstract, introduction, data availability) and we added the files to a new Zenodo DOI. (lines: 34, 560, 889)
- Concerning the large size of the Zenodo repository: we considered splitting it into various ones, but this could be on the other side also a hassle for some users. Instead of splitting its content into various Zenodo links, we now provide a description file which describes what each file contains.

3. The data processing pipeline on GitHub is very useful. The repository currently indicates a CC0 (public domain) license. Since CC0 is typically intended for datasets rather than source code, please clarify whether this was intentional or if a software-specific license (e.g., MIT, Apache 2.0) would be more appropriate.

Very good point. We initially had CC0 because we also had data in the repository (before creating the Zenodo link). It is indeed better to have a more suited license, like MIT. The repo has been updated accordingly.

4. A different typeface appears in some paragraphs (e.g., pp. 19 and 21). Please check whether this was intentional.

Yes, this was unintentional. It has been corrected.

5. The finding that grouping 5-17 samples per request does not substantially affect accuracy is interesting. Given that GPT models often fail with counting or item listing, the observed quality decline with larger chunk sizes seems reasonable and aligns with expectations.

It is indeed expected, and that is what we see with larger chunks. We think we should have also mentioned the mean there, because although 17 samples is the maximum per chunk (in a chunksize 3000) and the median is 5, the mean is actually 6. Taking the mean into consideration, our result ("no significant difference with no-chunking") becomes more intuitive. In the updated version of the manuscript we now report the mean. (line 481)
The pattern of biome accuracy dropping as larger chunks are used confirms the expectation of a detrimental effect when using more samples, even though significance (after post hoc) is reached only between 2000 and 6000 for biome and between 2000 and 5000 for sub-biome (sub-biome accuracy more is more sensitive to chunking). The model likely suffers from "context interference" once it exceeds its optimal working memory window.
It was interesting to us to observe which parts of a given chunk suffer more from this, and in some preliminary tests we investigated this. Our hypothesis was that the last or middle part samples were the ones more likely to get misannotated. What we found was that the middle ones were the ones to suffer most at chunksize 5000 and both (middle and last samples) to suffer the most at chunksize 6000 (see below). We did not include this analysis in the manuscript for the sake of conciseness.

6. On p. 26, the observed variability in field usage may be linked to the BioSample package system used for submission (see: https://www.ncbi.nlm.nih.gov/biosample/docs/packages/). Some fields, such as env_biome and env_feature, were once mandatory for environmental samples but are currently optional, I suppose. Such historical changes may partly explain biases in field usage.

That is an interesting point, and yes, it could help explain our results. We have added a sentence to the Discussion to acknowledge this as a possible contributing factor. (lines 806-809)

7. The manuscript appropriately highlights the presence of ambiguous or unresolvable sample descriptions. We reached a similar conclusion in our own work with local LLMs: in many cases, even expert curators cannot determine a "correct" label, and the right answer may depend on context or application.

We agree. Possibly, the implementation of more structured, hierarchical metadata submission forms could help mitigate this issue. For instance, a decision tree–type system that guides submitters through successive levels of sample description (e.g., first selecting “host-associated” vs. “free-living”, and then specifying subcategories) would greatly improve metadata consistency. This is along the lines of Thompson et al (2017) https://www.nature.com/articles/nature24621/figures/1. At the moment, clustering a posteriori of free-text sample descriptions is a major challenge.

8. The observation that JSON output significantly improves sub-biome classification accuracy is intriguing and consistent with our internal experience with local LLMs. Since output format may also affect processing speed, it would be useful to report whether response times differed between JSON and inline formats.

That' s an interesting point.
We have two inline-format GPT outputs that were completed in 57'' and 1'55'' (batch numbers: batch_67164dbbe8b081908bf02a4d2b6e2f65 and batch_67164db65fd0819097f31ac6c55e8caf, respectively).
The two json-format GPT output were completed in 59'' and 56'' (batch_67164da1e4ac81909991913126e9eecb and batch_67164d8ded048190badb01069142cbda, respectively).

Corresponding files (on Zenodo):

gpt_clean_output_nspb50_chunkingno_chunksize3000_modelgpt-3.5-turbo-1106_temp1.0_maxtokens4096_topp0.75_freqp0.25_presp1.5_rs22_formatjson_batch67164d8ded048190badb01069142cbda_dt202410211448.csv
gpt_clean_output_nspb50_chunkingno_chunksize3000_modelgpt-3.5-turbo-1106_temp1.0_maxtokens4096_topp0.75_freqp0.25_presp1.5_rs22_formatjson_batch67164da1e4ac81909991913126e9eecb_dt202410211448.csv
gpt_clean_output_nspb50_chunkingno_chunksize3000_modelgpt-3.5-turbo-1106_temp1.0_maxtokens4096_topp0.75_freqp0.25_presp1.5_rs22_formatinline_batch67164db65fd0819097f31ac6c55e8caf_dt202410211448.csv
gpt_clean_output_nspb50_chunkingno_chunksize3000_modelgpt-3.5-turbo-1106_temp1.0_maxtokens4096_topp0.75_freqp0.25_presp1.5_rs22_formatinline_batch67164dbbe8b081908bf02a4d2b6e2f65_dt202410211449.csv

Based on these results currently included in the manuscript (and uploaded on Zenodo), we have too few data points to draw conclusions on speed.

However, we were curious and ran 10 more async runs for inline and 10 more async runs for json (each run 2x the size of samples as the above i.e. 100 nspb → 500 samples in total). We did so by alternating the requests (1 inline, 1 json, 1 inline, 1 json, and so forth). These are the completion times we obtained:

inline: 18', 17', 20', 3' 17'', 20', 18', 18', 4' 43'', 17', 20'
json: 3' 49'', 3' 13'', 17', 3' 13'', 11', 18', 18', 17', 17', 17'

Powered by Editorial Manager® and ProduXion Manager® from Aries Systems Corporation

Inline stats:
Mean: 936.0 s (15.60 min)
Median: 1080.0 s (18.00 min)
SD: 373.9 s (6.23 min)
SEM: 118.2 s (1.97 min)

JSON stats:
Mean: 751.5 s (12.53 min)
Median: 1020.0 s (17.00 min)
SD: 395.7 s (6.60 min)
SEM: 125.1 s (2.09 min)

Although there seems to be a small difference, it doesn' t reach significance (Welch's T-test: t = 1.072, p = 0.2981).

We think it's likely that with a larger sample size, we would observe that json format are processed faster than inline, but with the data we currently have and the test we performed we can' t draw the conclusion. Importantly, it needs to be taken into consideration that completion times for remote LLM queries can be confounded by variable resource allocation and network conditions, making them inherently noisy.

9. One major limitation of the study is the dependence on proprietary GPT models accessible only via OpenAI's API. This constrains reproducibility and long-term availability. Indeed, the recent release of GPT-5 already renders some of the reported results outdated. While the present study remains highly valuable, it would be worthwhile to also evaluate local or open-source LLMs to ensure future reproducibility.

We completely agree with Reviewer 1. We now have included open-weight models in our analysis and manuscript, and we also added two more recent GPT models (GPT-4.1 and GPT-5-mini) to make the study more current.
To our surprise, we found that several open-weight models performed similarly to proprietary GPT models in both biome and sub-biome classification accuracy, while other models were less performant. We found that Qwen3-Next-80B-A3B-Instruct achieved comparable or even slightly higher accuracy than GPT-4.1 and GPT-5-mini, while models such as microsoft-phi-4 also demonstrated strong, consistent performance. (lines 593-617)
We also found that Qwen embedding models (also open-weight) consistently outperformed OpenAI's text-embedding-3-small. (618-637)
In our revised discussion we report a cost comparison (open-weight models were typically 10–50 times lower than proprietary GPT models) and we also clarify why there is a cost to begin with: although open-weight models are freely available, running them locally requires dedicated GPU resources, and at present it is often more economical to use third-party hosting platforms (e.g., DeepInfra) than to purchase and maintain GPUs on site. (638-647)
We are particularly glad to have conducted this analysis in response to Reviewer 1's suggestion, as it delivers a strong message: ecological metadata annotation is no longer restricted to commercial APIs. Open-weight models can be run repeatedly to build consensus across predictions, increasing confidence in the annotations. Besides the substantial cost reductions, the use of open-weight models enhances transparency, reproducibility, and long-term sustainability. Overall, this additional analysis proved well worth the effort, as it strengthened the manuscript both scientifically and conceptually by demonstrating that high-quality, reproducible metadata annotation can now be achieved with open tools.

Reviewer #2: Reproducibility report for: Enhanced semantic classification of microbiome sample origins using Large Language Models
Journal: Gigascience
ID number/DOI: GIGA-D-25-00316
Reviewer(s): Laura Caquelin, Department of Clinical Neuroscience, Karolinska Institutet, Sweden [Wrote the report and reproduced the results]

----

1. Summary of the Study

This study evaluates whether Large Language Models (LLMs) can help re-annotate sequencing records. Using GPT models, the authors tested scalability, time, cost, and performance against a benchmark of 1,000 hand-curated examples. They then applied this approach to million environmental sequencing records, producing standardized annotations.

----

2. Scope of reproducibility

According to our assessment the primary objective is: to evaluate how closely GPT's annotation performance approached that a human expert when classifying environmental sequencing samples into biomes and sub-biomes.

- Outcome: Accuracy of biome and sub-biome classification compared against a hand-curated benchmark dataset.

- Analysis method outcome: As described to validate biome classifications: "For paired comparisons of repeated sample IDs, we use the McNemar test, which is appropriate for paired binary outcomes (True/False)", while "for comparisons across different sample sets, we employ the t-test for independent samples. In both scenarios, a Bonferroni correction is applied to adjust for multiple comparisons".

For sub-biomes, comparisons across different sets were performed with independent t-tests, while "for runs involving the same sample IDs, comparisons are performed using the paired t-test". Section "Validation statistics" page 13-14.

- Main result: "The improvement in accuracy between GPT's initial classification and the human's performance with the improved prompt was statistically significant (adj p-value=0.031), but also between the human's attempt first attempt (with the initial prompt) and the human's second attempt (better prompt) (adj p-value≤0.001). No significantly different performances were detected for the sub-biome classification, neither between GPT and the human, nor between prompt versions (adjp-value=1)." Section "Human versus GPT classification accuracy" pages 18-19.

----

3. Availability of Materials

a. Data

- Data availability: Open
- Data completeness: Complete = all data necessary to reproduce main results are available
- Access Method: Repository
- Repository: https://zenodo.org/records/16100607
- Data quality: Complete but no metadata associated with the file

Yes, that is correct, we did not add the file to the Zenodo repository. Instead we only put it on the Github repo. The file is called “MicrobeAtlasProject_files_list_Zenodo.tsv”. In the new Zenodo upload (link reported in the revised manuscript), we now include this file.

b. Code

- Code availability: Open
- Programming Language(s): Python
- Repository link: https://github.com/GaioTransposon/metadata_mining/tree/main
- License: CC0
- Repository status: Public
- Documentation: Readme file clear but require one modification

Yes, that is correct. We updated the README.md file to avoid confusing users into

making ~/MicrobeAtlasProject/MicrobeAtlasProject. We updated the “Requirements” section of the README.md file.

----

4. Computational environment of reproduction analysis

- Operating system for reproduction: MacOS 15.6.1
- Programming Language(s): Python
- Code implementation approach: Using shared code
- Version environment for reproduction: Python3.13.7

----

5. Results

5.1 Original study results

- Results:

"The improvement in accuracy between GPT's initial classification and the human's performance with the improved prompt was statistically significant (adj p-value=0.031), but also between the human's attempt first attempt (with the initial prompt) and the human's second attempt (better prompt) (adj p-value≤0.001). No significantly different performances were detected for the sub-biome classification, neither between GPT and the human, nor between prompt versions (adjp-value=1)."

(The authors identified an error in the manuscript text during the review. Therefore, the following part of the manuscript needs to be updated (see email exchange with the authors below).

5.2 Steps for reproduction

-> Run the two first scripts of the container 4 in the Github: validate_biomes_subbiomes.py and overall_analysis.py

- Issue 1: The README instructions for setting up the ~/MicrobeAtlasProject directory can lead to a nested folder structure (~/MicrobeAtlasProject/MicrobeAtlasProject) if followed literally. This causes the Docker container to fail when attempting to access required files like gpt_file_label_map.tsv, since they are not found at the expected path /MicrobeAtlasProject/.

-- Resolved: The issue was resolved by manually renaming and flattening the directory structure after extraction, ensuring that the contents of MicrobeAtlasProject_Zenodo are directly placed inside ~/MicrobeAtlasProject/. However, the current instructions can mislead users, so a clarification in the README would be helpful.

Yes, that is correct. We updated the README.md file to avoid confusing users into making ~/MicrobeAtlasProject/MicrobeAtlasProject. We updated the “Requirements” section of the README.md file.

- Issue 2: During the execution of the overall_analysis.py script, multiple files with the same label were found, requiring manual selection of the file to use for the analysis.

-- Resolved: The manuscript does not specify which file should be selected to reproduce the results, leading to potential ambiguity. By default, I chose the most recent file among the options, assuming it reflects the final data version used in the manuscript. It would be helpful if the documentation or manuscript explicitly stated this to ensure exact reproducibility.

This was a very good point. We now edited overall_analysis.py to not prompt the user to choose from any “double labeled” file. This issue occurred because multiple GPT outputs shared identical parameters but different timestamps. As reviewer 2 suggested

this caused unnecessary ambiguity. The solution we found was to remove the unnecessary GPT output files from the Zenodo repository (now reflected in the current Zenodo version).

-> Compare the results reproduced to the results presented in the manuscript

- Issue 3: The results obtained by running validate_biomes_subbiomes.py are two files: biome_subbiome_results.csv and biome_subbiome_stats.csv, which contain a large amount of output (1,284 and 48,197 rows respectively). The script overall_analysis.py provides overall performance metrics in the terminal output, but does not produce the adjusted p-values relevant to the scope of this review.

-- Unresolved: It was difficult to identify where to find the results presented in the manuscript, so an email was sent to the authors.

Yes, this was a main issue which has now been resolved. Those fairly large files (1,284 and 48,197 rows) were caused by the script concatenating on an existing file, as mentioned in our correspondence. Now the script validate_biomes_subbiomes.py has been updated to create at each run new biome_subbiome_results*.csv and biome_subbiome_stats*.csv files. There was additionally a problem with the script, that arose silently when editing the code for Docker (after our first submission and under request of the Editor). Without us realizing, the code: 1. silently filled out empty fields of biome_subbiome_results.csv and biome_subbiome_stats.csv even when embeddings .json files were missing; 2. did not group by test_type properly, so it did a comparison of everything against everything, generating an unnecessarily large file, very hard to read.
The removal of the 20 gpt output files caused some values to slightly drift. Here we list the changes (please note that the page and line numbers refer to the previous manuscript version):
-Line 395: “(precision: 81.7%, F1-score: 80.16).“ → “(precision: 82.0%, F1-score: 80.34).”
-Line 396: “n=125” → “n=105” (this is the current number of files)
-Line 397: “F1-score: 76.1%” → “F1-score: 76.4%”
-Line 400: “accuracy rates of 93.6% and 94.3%” → “accuracy with accuracy rates of 94.6% and 93.8%”
-Line 401: “In contrast, ‘plant’, ‘water’, and ‘other’ biome samples exhibit lower accuracy rates (67.2%, 79.3%, and 67.5%, respectively)” → “In contrast, ‘plant’, ‘water’, and ‘other’ biome samples exhibit lower accuracy rates (67.3%, 79.9%, and 67.2%, respectively)”
-Line 405: “precision (81.7%” → “precision (81.9%”
-Line 406: “accuracy: 93.6%” → “accuracy: 93.8%”
-Line 407: “precision: 87.8% vs. 8.24%” → “precision: 87.8% vs. 82.4%” (this was a typo)
-Lines 408-411: “While both methods struggle with plant samples, which they often misclassified as soil (Fig. 2A-B), GPT again produced fewer misclassifications on this subset (4.0% vs. 17.8%). In addition, GPT shows improved predictions in the reverse direction by mislabeling soil samples less frequently as plant biome, compared to the keyword-based classifier (1.3% vs. 11.5%).” → “Both methods struggle with plant samples, which they often misclassified as soil (Fig. 2A-B). Whereas GPT most often misclassified them as ‘soil’ (27.0%), the keyword-based classifier repartitioned the misclassified samples into either ‘soil’ (16.8%) or ‘other’ (17.8%). “ (here the values are updated as well as the phrasing)
-Line 413: “as indicated by Cohen’s kappa values (GPT: 0.757” → “as indicated by Cohen’s kappa values (GPT: 0.760”
-Line 420: “chi-squared” → chi-squared: 7482
-Line 421: “A large fraction of misclassified ‘animal’ samples (49.9%) are erroneously predicted as ‘water’.” → “A large fraction of misclassified ‘animal’ samples (53.0%) are erroneously predicted as ‘water’.”
-Lines 420-423: “misclassifications within ‘plant’ (82.9%) and ‘water’ samples (59.1%) are ‘soil’” → “misclassifications within ‘plant’ (83.9%) and ‘water’ samples (59.0%) are ‘soil’”
-Lines 424-425: “skewness: 1.32; kurtosis: 0.23” → “skewness: 1.34; kurtosis: 0.27”
-Lines 426-427: “Notably, 23 samples (5% of the total) fall at or above the 95th percentile, with 109 or more misclassifications” → “Notably, 21 samples (5.3% of the

Powered by Editorial Manager® and ProduXion Manager® from Aries Systems Corporation

total) fall at or above the 95th percentile, with 90 or more misclassifications”
-Lines 428-430: “Across 117 independent classification runs using different parameter settings, the average number of misclassifications per sample is 26.0 (SD: 36.0).” → “Across 105 independent classification runs using different parameter settings, the average number of misclassifications per run is 21.7 (SD: 29.9).”
-Lines 430-431: “Half of the samples are misclassified 5 times or fewer, and three-quarters have no more than 41 misclassifications” → “Half of the samples are misclassified 4 times or fewer, and three-quarters have no more than 34 misclassifications”
-Line 432: “The maximum observed was 117” → “The maximum observed was 97”
-Line 436: “SRS4776621” → “SRS5304049” (this is another rhizosphere sample that popped in the updated results - the same count for this sample: it was misclassified as soil by GPT in all but one case, as mentioned at the end of the paragraph).
-Line 438: “was incorrectly assigned to ‘water’ by GPT in 109 out of 117 instances” → “was incorrectly assigned to ‘water’ by GPT in 89 out of 97 instances”
-Line 441: “assigned to ‘animal’ by GPT in 109 out of 112 instances” → “assigned to ‘animal’ by GPT in 90 out of 93 instances”
-Line 522: “accuracy was mitigated (freqp 0.0: 80.76%; freqp 2.0: 80.96%)” → “accuracy was mitigated (freqp 0.0: 79.16%; freqp 2.0: 78.31%)”
-Lines 537-553: we worded this part about the replicates better to clarify that the lower accuracy ranges seen across the sync requests compared to the async requests are due to the format difference and not the diff between sync and async. Sync versus async are the 6 replicates (times 2) results we clarify in the last paragraph.

As we refreshed the gpt files (removed the unnecessary ones) the geographic location analysis values also changed. At that point, given that we had many more samples available from the production run (unique ones), we updated the section “Geographic location prediction performance” to include all samples (990,172 instead of the previous 130,689). NB: these are not over 2M because it’s a merge between samples that were geographically annotated by GPT and samples from which metadata we could retrieve the coordinates, hence 990k only. We found values in this section to have shifted: 95.32% vs 95.94% matches, now versus before.
As we did not re-perform the manual-picking inside done in geo_check.py (picking manually 100 samples metadata and deciding who is right and why - gpt vs coordinates), we could use the same dictionary that was created earlier (it’s an argument of geo_check.py). The only thing that changed is the sample size of the analysis, and therefore also the map figures (now Figure 7 and Suppl. Figure 8).

Message sent by the authors

Dear reviewer,

By running validate_biomes_subbiomes.py as described (using gpt_file_label_map.tsv as --map_file), the output will be two .csv files namedbiome_subbiome_results.csv andbiome_subbiome_stats.csv.

The latter file will contain the stats (hence the adjusted p-values).Were you able to reproduce such files?

We did notice there are a few mistakes.

Mistake 1.

In the manuscript it says:

" A trained molecular biologist, with no prior exposure to the project, was given the same prompt instructions as GPT and was asked to classify sample biomes and sub-biomes. While against the benchmark dataset, GPT achieved an accuracy of 79.76% (n=499; SD=40.0), the human annotator reached 78.0% (n=250;SD=33.0). "

The second standard deviation should be replaced withSD=42.0.

We re-confirm this change.

Powered by Editorial Manager® and ProduXion Manager® from Aries Systems Corporation

Mistake 2.

In the manuscript it says:

" The improvement in accuracy between GPT's initial classification and the human's performance with the improved prompt was statistically significant (adj p-value=0.031),but also between the human'sattemptfirst attempt (with the initial prompt) and the human's second attempt (better prompt) (adj p-value≤0.001)."

The first adjusted p-value should not be 0.031 but 0.134 hence not significant so this sentence should be adjusted to:

" There was an improvement in accuracy between the human's first attempt (with the initial prompt) and the human's second attempt (better prompt) (adj p-value≤0.001)."

We re-confirm this change.

Mistake 3.

We built onbiome_subbiome_results.csv andbiome_subbiome_stats.csv further than necessary so the two files on Zenodo should be "cut" earlier to avoid confusion. This was a problem of the scriptvalidate_biomes_subbiomes.py whichconcatenates on existing files (e.g.:biome_subbiome_results.csv andbiome_subbiome_stats.csv) instead of creating new ones. We should probably proceed by replacing these two files with the files without repetitions.

We thank you for your work and please do let us know if everything works out now.

Thank you and kind regards,

We re-confirm this change. Yes, the new Zenodo link has the correct files. The script (as mentioned above) now does not concatenate on existing files anymore, but produces fresh ones when re-run.

####

The authors confirm that the results presented in the manuscript can be found in the biome_subbiome_stats.csv file. This file contains 48197 rows. According to the authors, the file includes data from both existing files and new data. As a result, it is difficult to determine which data have been reproduced. Even when using a Ctrl+F search for the reported p-value (e.g, pvalue = 0.134) in the Excel file, this value appears in several rows labeled under different configurations such as (label1/label2):
--- chunk_size3000/sync_chunkN_presp0.0;
--- chunk_size3000/sync_chunkN_temp1.5;
--- chunk_size5000/gpt4-0613;
--- machine/ sync_chunkY_topp0.0, etc…

Yes, as mentioned above we solved this problem by 1. Replacing the correct biome_subbiome_results*.csv and biome_subbiome_stats*.csv files; 2. De-bugging validate_biomes_subbiomes.py. When we edited our scripts to work with Docker, some issues had arisen that we were unaware of. One was the script silently filling out empty fields of biome_subbiome_results.csv and biome_subbiome_stats.csv even when embeddings .json files were missing. Another issue was not properly grouping by test_type properly, so the output was very hard to read. The strange label pairing Reviewer 2 noticed, no longer happens now. Only files falling under the same column “test_type” (in gpt_file_label_map.tsv file), are compared to each other. It wouldn’t make sense to compare for example “chunk_size3000” with “sync_chunkN_presp0.0”. This is also why the biome_subbiome_stats.csv file was so large. We thank Reviewer 2 for spotting this important problem.

5.3 Statistical comparison Original vs Reproduced results

- Results: The biome_subbiome_stats.csv file was reproduced, but it is difficult to

Powered by Editorial Manager® and ProduXion Manager® from Aries Systems Corporation

distinguish between the newly reproduced data and the existing data already present in the file. Additionally, the data presented in the manuscript are also hard to identify due to the size of the file. No comparison was performed.

Biome_subbiome_stats*.csv (the * now stands as we have three files each, after the addition of the new analysis with the open-weight models) now only produces the correct comparisons, and there is no concatenation to the existing file. With the file now is being concise, we hope we have overcome the problem of poor readability. We stay open to further improvements where found necessary.

- Comments: -
- Errors detected: Authors identified an error in the manuscript text during the review with the first adjusted p-value that is not 0.031 but 0.134.

We re-confirm this change.

- Statistical Consistency: No comparison was performed.
----

6. Conclusion

- Summary of the computational reproducibility review

The main scripts to reproduced the results were successfully executed and the output files were generated. However, due to the size of the output files and the lack of precise references in the manuscript, it was difficult to identify which parts of the output correspond to the results presented in the paper. Moreover, authors mentionned that the script adds data to existing output files rather than generating new ones, making it hard to distinguish between old and new data. This led to confusion when trying to compare the reproduced results with those in the manuscript. Then a comparison of statistical values was not possible.

- Recommendations for authors

To improve the reproducibility of the manuscript, we recommend the authors to:

-- Clarify instructions in the README about the MicrobeAtlasProject folder.

Done.

-- Ensure scripts generate new outputs or clarify which data is new vs. existing in the files with for example a column indicating the origin (e.g., "new" or "existing").

Done. It now generates a new output at each run. No more concatenation.

-- Link results in the manuscript to specific rows/sections in the output files to easily locate the exact data used. Another solution could be to consider including a smaller, or a filtered version of the output files with only the rows used for key reults, figures or tables, to make checking results easier and avoid error.

This is a good suggestion by Reviewer 2, but we were wondering whether the addition in the manuscript of “see line xx of file yy” every time result values or stats are reported, would not make it much heavier to read. We believe now we have significantly reduced the size of the two files so the values are easier to read. Also correcting the issue of random labels being compared to random labels, has solved a major problem. As a result, values are easier to find. The results file is now 154 lines long (instead of 1,284) and the stats file now has 1,172 lines (instead of 48,197). It is worth noting that the new files also include the new output files generated for the comparison of OpenAI with open-weight models (new results - under request of Reviewer 1).

-- Metadata: For the data used or generated by the scripts, it would be helpful to include accompanying metadata files that explain:
--- The definition of each variable name.

Powered by Editorial Manager® and ProduXion Manager® from Aries Systems Corporation

--- The origin of each dataset (raw, processed, etc).
--- Any preprocessing steps applied before analysis.

Yes, this file is “MicrobeAtlasProject_files_list_Zenodo.tsv”, which we previously uploaded to our Github repo, but not on the Zenodo repository. It showed on the front page of the Zenodo repository, but it’s not downloadable. In the new Zenodo version we now include the file. Besides making the file downloadable, we expanded the descriptions for each file, as Reviewer 2 suggested.

Reviewer #3: The ability to reuse scientific data for secondary analysis is an extremely important topic. Since the promotion of the FAIR Guiding Principles a decade ago, the central importance of standards-adherent metadata has received considerable attention. Although this paper surprisingly doesn't mention the FAIR principles, the work is important in understanding what it takes to make datasets FAIR and "AI ready."

We thank Reviewer 3 for this valuable comment and for emphasizing the connection between our work and the FAIR principles. We agree that our study directly contributes to advancing dataset FAIRness and AI readiness through large-scale metadata standardization using LLMs. In response, we have revised the manuscript to explicitly acknowledge and discuss this connection with FAIR. We did so in the Abstract (line 18), Intro (lines 66,75), Results (line 386) and Discussion (lines 722, 878).

A core problem with the paper is that it is unsure who its audience is. The paper is motivated by the needs to scientists to search for and reuse online datasets for secondary analysis, but much of the paper concerns highly technical issues that are related to fine tuning LLM performance. It is laudable that the manuscript annotates its discussion of the authors' methods with pointers to actual Python scripts that would allow third parties to replicate the authors' work. The detailed presentation, however, may make it hard for many readers to understand the computational strategy that all the scripts are implementing. The organization of the paper weaves from discussions of the ability of LLMs to extract scientific standards from "legacy" experimental metadata to details of how to enhance computational efficiency to make the use of LLMs more cost-effective. The title and abstract of the paper suggest that the authors are aiming for a more scientific audience, but much of the manuscript focuses on arcane implementation details that will be less important to such readers.

We thank Reviewer 3 for the thoughtful and constructive comment, and we appreciate the recognition of our efforts to make the pipeline fully transparent and reproducible. Our goal with this manuscript is indeed to reach several audiences:
(1) computational researchers or bioinformaticians who wish to reproduce or extend the pipeline, and
(2) scientists from molecular biology, microbiology, or ecology who regularly encounter poorly structured metadata and wish to understand how far LLMs have progressed toward assisting human curation, and
(3) scientists from molecular biology, microbiology, or ecology who operate at the intersection of experimental and computational biology.
We acknowledge that bridging these communities is ambitious, but it reflects the evolving nature of biological data science, where researchers increasingly operate at the intersection of experimental and computational fields. At present, there is no broadly adopted solution to address the widespread problem of non-standardized or effectively unusable metadata in public repositories. Our work aims to fill this gap by providing both conceptual guidance and a reproducible, step-by-step framework for improving metadata usability through LLM-based re-annotation.
At the same time, we acknowledge that the manuscript provides full transparency at the cost of lots of detail (for example explaining what each script does).Users that do not want to extend or modify the scripts will perhaps not need that level of verbosity and granularity - for them, a concise README file in the GitHub repo walks step-by-step through reproducing the pipeline.
To make the paper easier to follow for non-technical readers, we have now added an overview paragraph at the start of the Results section summarizing the logical flow of analyses and highlighting how each component connects to the study’s central question (lines 370-390). This addition helps maintain orientation through the more detailed methodological subsections that follow.

Powered by Editorial Manager® and ProduXion Manager® from Aries Systems Corporation

Missing from the paper is a detailed discussion of what the metadata in SRA are really like. The reader never sees complete examples of the metadata that are processed in the authors' work, and thus it is hard to have intuition about the problem that the authors are trying to solve. In particular, the paper doesn't present information about the range of attributes in user-defined metadata fields in SRA. The paper would benefit from a discussion of the structure of scientific metadata in general, and of how the authors' work fits into the larger effort in the research community to make datasets FAIR. (Full disclosure: My own laboratory is involved in such activity. See https://arxiv.org/abs/2504.05307v2)

We thank Reviewer 3 for this valuable comment and for sharing their reference, which we have cited in the revised manuscript. We agree that showing concrete examples of metadata structure helps readers better appreciate the challenges addressed by our work. To that end, we have expanded the Introduction to include a discussion of typical metadata inconsistencies, now citing Ikeda et al. (2025), who document malformed BioSample entries, as well as our own representative metadata examples (added as Supplementary Figure 1). These additions provide direct insight into the heterogeneity and irregularities of BioSample metadata, highlighting why automated interpretation is required.

Regarding the “range of attributes in user-defined metadata fields in SRA,” this topic is addressed in the Results section (Informative metadata fields), which details the diversity of fields used to describe sample origin, and is further supported by Supplementary Figure 9. Together, these additions provide both a qualitative and quantitative view of the metadata landscape.

Finally, we note that while a deeper exploration of the metadata problem itself would indeed be valuable, we had to balance scope with readability, given that the primary focus of the manuscript is the evaluation of GPT and open-weight LLM performance for metadata re-annotation. The current version now clarifies the metadata challenges motivating this task while keeping the main emphasis on methodological evaluation and comparative performance.

The abstract of the paper states that the authors "test to what extent LLMs can be used to cost-effectively automate the re-annotation of sequencing records." Alas, the paper really examines re-annotation of only the fields for "biome" and "location." A weakness of the paper is that the reader doesn't learn what other fields may be relevant in these metadata records, and why the authors chose to focus on the particular fields that they studied. Overall, much more attention should be placed on discussion of the limitations of the work and how well the results might scale to more general problems in standardization of scientific metadata.

We agree that our analysis focuses on a subset of metadata fields and that this limitation should be explicitly acknowledged. In the revised version, we have added a paragraph to the Discussion describing this scope, clarifying that our validation was restricted to fields related to sample origin, namely biome, sub-biome, and geographic location. These attributes were chosen because they are central to the MicrobeAtlas project, which serves as the empirical foundation for this study, and because they apply broadly to all sequencing samples regardless of context. They are also among the most inconsistently reported fields in public repositories, making them particularly relevant for testing LLM-based re-annotation.

Other metadata fields (e.g., environmental parameters such as pH, altitude, or ocean depth; or methodological fields such as sequencing platform and library type) are indeed important for downstream analyses but were beyond the scope of this work. Many of these are numeric or at least more highly structured and can be parsed more reliably using conventional NLP or rule-based methods, whereas the biome and sub-biome fields require nuanced semantic interpretation—an area where LLMs can add the most value.

The newly added section in the Discussion explicitly acknowledges this as a limitation of the current study and frames it as a first step toward more comprehensive LLM-driven metadata standardization. We emphasize that the framework presented here can, in principle, be extended to additional metadata fields in future work.

Powered by Editorial Manager® and ProduXion Manager® from Aries Systems Corporation

	Minor comments:<br><br>The term "biome" is never well defined.<br><br>We acknowledge a proper biome definition was missing earlier on. In the revised version we added the definition to the introduction (lines 105-114), methods (lines 174-194); and results section (lines 388-390).<br><br>Frequently, parenthetical remarks begin with "e.g." and end with "etc." This style is redundant; you need only one of these abbreviations in each instance.<br><br>Good point. We corrected this.<br><br>Page 6, para 1: "last three digits" or "last three characters"?<br><br>We relied on the “last three digits” because all sample IDs terminate with digits, whether they are SRS, ERS or DRS. For example: SRS1713087, ERS3774001, DRS005001. Using these digits to define directory names provided a simple and deterministic way to distribute millions of metadata files across subdirectories. This structure was essential for filesystem performance and scalability: storing millions of files in a single directory can dramatically slow down file access, indexing, and I/O operations. By splitting the files into subdirectories named after the last three digits, we ensured that each folder contained a manageable number of files, enabling faster read/write operations and easier parallel processing. This approach benefits both our own workflow as well as any user intending to reproduce the pipeline.<br><br>What is the motivation for consolidating reference ontologies into a single dictionary?<br><br>This was primarily a practical and logistic choice. By consolidating them into one, users who want to reproduce the pipeline can for example directly use our pre-made dictionary (downloadable from the zenodo link in the revised manuscript), or even compare the checksum of our dictionary with theirs to know if it differs (simpler and more transparent with one unified file than with many smaller ones). Also, a single consolidated dictionary ensures consistent ontology mapping across the entire pipeline. It eliminates potential discrepancies that could arise if individual ontology files were updated independently or parsed in different orders. In this way, lookup and caching during annotation are faster, since the script can load one unified key–value structure into memory rather than repeatedly querying multiple smaller files.<br><br>Page 13, para 2: The notion of "lenient matches" requires much more discussion. If the goal is to make the legacy metadata standards-adherent, then a "lenient" match would not seem to be valid. The operative question is, "What metadata terms will users invoke to search for datasets?", and presumably users will be searching for standard terms only.<br>We observed that, despite strict prompt instructions to output only one of the five allowed biome labels (“animal”, “plant”, “water”, “soil”, “other”), LLMs occasionally appended short clarifying text such as “animal (human)” or “water (marine)”. These are still fully valid biome classifications—albeit somewhat more specific—but are not exact string matches to the benchmark. To quantify performance in these cases, we defined a lenient match as an output where one of the five biome labels appears as a complete word (e.g., “animal (human)” counts as a lenient match to “animal”). This does not include partial or fuzzy matches (e.g., “plan” ≠ “plant”). This criterion was only applied when assessing biome classification accuracy and only to highlight the model’s tendency to add detail when certain creativity parameters (i.e.: frequency penalty >0.25) were used during chunked requests (Figur...
**Additional Information:**	
**Question**	**Response**
Are you submitting this manuscript to a special series or article collection?	No
**Experimental design and statistics**	Yes

Powered by Editorial Manager® and ProduXion Manager® from Aries Systems Corporation

Full details of the experimental design and statistical methods used should be given in the Methods section, as detailed in our Minimum Standards Reporting Checklist. Information essential to interpreting the data presented should be made available in the figure legends.<br><br>Have you included all the information requested in your manuscript?	
**Resources**<br><br>A description of all resources used, including antibodies, cell lines, animals and software tools, with enough information to allow them to be uniquely identified, should be included in the Methods section. Authors are strongly encouraged to cite Research Resource Identifiers (RRIDs) for antibodies, model organisms and tools, where possible.<br><br>Have you included the information requested as detailed in our Minimum Standards Reporting Checklist?	Yes
**Availability of data and materials**<br><br>All datasets and code on which the conclusions of the paper rely must be either included in your submission or deposited in publicly available repositories (where available and ethically appropriate), referencing such data using a unique identifier in the references and in the "Availability of Data and Materials" section of your manuscript.<br><br>Have you have met the above requirement as detailed in our Minimum Standards Reporting Checklist?	Yes
GigaScience has policies and guidelines in place for the use of generative AI-	No

writing tools such as ChatGPT. If you have used such writing tools to assist with writing the manuscript this must be declared and cited in the text. Authors should not list AI-writing tools and other AI-assisted technologies as an author or co-author and should acknowledge that they are fully responsible for text generated or refined by AI-writing tools.<p><br><br>A summary of use (particularly in the introduction or among methods) needs to be included at the end of the paper, and the outputs should also be included as a supplementary file hosted in GigaDB or other open repositories. Please read our guidelines for more information. <p><br><br>By submitting to GigaScience, you are aware of the journal's AI-writing tools policy, and if you have declared use of such tools below, you have acknowledged this where appropriate in your manuscript and have made a summary of use and outputs available. <p><br><b>AI-assisted writing tools have been used in the preparation of this manuscript?	

Manuscript

Click here to access/download;Manuscript;openAI_manuscript_Feb 11.docx

Enhanced semantic classification of microbiome sample origins using Large Language Models (LLMs)

Daniela Gaio[1], Janko Tackmann[1], Eugenio Perez-Molphe-Montoya[1], Nicolas Näpflin[1], David Patsch[1], Lukas Malfertheiner[1], Matteo Eustachio Peluso[1], Christian von Mering[1]

[1]Department of Molecular Life Sciences and Swiss Institute of Bioinformatics, University of Zürich, Zürich, CH-8057, Switzerland

ORCIDs: Daniela Gaio [0000-0002-7695-3145]; Janko Tackmann [0000-0003-1467-2863]; Eugenio Perez-Molphe-Montoya [0009-0002-9592-9455]; Nicolas Näpflin [0000-0001-6845-7400]; David Patsch [0009-0002-9859-091X]; Lukas Malfertheiner [0000-0002-5697-2007]; Christian von Mering [0000-0001-7734-9102]

Abstract

Over the past decade, central sequence repositories have expanded significantly in size. This vast accumulation of data holds value and enables further studies, provided that the data entries are well annotated. However, the submitter-provided metadata of sequencing records can be of heterogeneous quality, presenting significant challenges for re-use. Here, we test to what extent large language models (LLMs) can be used to

cost-effectively automate the re-annotation of sequencing records against a simplified classification scheme of broad ecological environments with relevance to microbiome studies, without fine-tuning. This effort directly contributes to improving the FAIRness—Findability, Accessibility, Interoperability, and Reusability—of microbiome sequencing metadata, thereby enhancing their “AI readiness” for downstream computational analyses.

We focused on sequencing samples taken from the environment, for which metadata is important. We employed OpenAI Generative Pre-trained Transformer (GPT) models, and assessed scalability, time- and cost-effectiveness, as well as performance against a diverse, hand-curated benchmark with 1,000 examples, that span a wide range of complexity in metadata interpretation. Annotation performance markedly outperformed that of a baseline, manually curated, non-ML keyword-based approach. Changing models (or model parameters) has only minor effects on performance, but prompts need to be carefully designed to match the task. Furthermore, when we compared proprietary OpenAI models with open-weight alternatives (*e.g.*, Qwen, meta-Llama, and microsoft-phi-4), we found comparable accuracy for both biome and sub-biome classification, indicating that open-weight architectures can match the performance of proprietary models for large-scale ecological metadata re-annotation.

We validated the pipeline with 1,000 hand-curated samples, and we applied the optimized pipeline to 2 million sequencing records from the environment, providing coarse-grained yet standardized sample origin annotations covering the globe. Our

work demonstrates the effective use of LLMs to simplify and standardize annotation from complex biological metadata.

Introduction

Streamlining and standardizing metadata is crucial for ensuring reproducibility in scientific research. Over the past decade, the size of the GenBank database has expanded by more than 30-fold, the whole-genome sequencing database (WGS) grew nearly 40-fold, and the European Nucleotide Archive (ENA) reported a 10-fold increase in a window from 2012 to 2022 [1]. This vast increase underscores the necessity of managing, standardizing, and utilizing such large datasets effectively. Metadata accompanies all scientific data types, and primary data repositories provide submitters with guidelines and facilities for providing structured metadata at the time of submission. However, the submission step—critical as it is—often does not receive as much attention as earlier steps, such as sample collection and processing. Samples with well-organized metadata are more likely to be reused [2–4], indicating that thoughtful metadata submission enhances broader research utilization. In the case of raw DNA sequence data, the National Center for Biotechnology Information (NCBI) SRA submission system suggests a number of metadata fields to be filled, along with controlled vocabularies. However, as is common in many databases, only a minimal number of fields are mandatory, leaving submitters with considerable discretion in how information is provided and formatted. This flexibility, while beneficial for submitters, frequently results in metadata that is challenging to reuse. Similar issues

have been reported in public repositories. A recent study documented BioSample records containing incomplete or nonsensical attribute entries such as placeholder tags ("TODO: TAG NAME"), random strings ("ACAGACAGCGT"), or symbols, highlighting how metadata irregularities can render otherwise valuable datasets difficult to interpret and reuse [5]. We observed comparable problems where metadata entries were often verbose, inconsistent, or redundantly encoded across fields (Supplementary Figure 1). These examples illustrate how even syntactically valid metadata can remain semantically inconsistent, complicating automated parsing and large-scale re-annotation. These challenges directly relate to the FAIR Guiding Principles, which emphasize that data should be Findable, Accessible, Interoperable, and Reusable. While these principles have guided data stewardship for a decade, achieving them in practice remains difficult, particularly for legacy datasets with inconsistent or unstructured metadata. Over time, NCBI has introduced measures to mitigate this issue, such as pre-populated drop-down menus for fields like *organism_type*, and since June 2023, they provide a tutorial to guide submission. While these changes help standardize entries, they are unlikely to completely solve the issue [5]. Moreover, prior submissions that allowed free-form data entry remain difficult to standardize retroactively. Addressing these shortcomings is essential to make large-scale sequencing repositories both FAIR-compliant and "AI ready," ensuring that they can support automated discovery and integrative analyses [6].

The rise of artificial intelligence (AI) in metadata parsing marks a significant evolution from earlier efforts using traditional Natural Language Processing (NLP) methods [5,7–9] or techniques that use term frequency (*e.g.*, term frequency-inverse

document frequency, *i.e.*, TF-IDF) [10] to identify key terms within text-based metadata. The complexity of such metadata, which can range from full sentences to acronyms, uses niche terminology and frequently includes spelling variants or typos, posing substantial challenges to traditional NLP and term frequency methods. Relying solely on term frequency, even when metadata is articulated clearly, can be inadequate for good classification, as these methodologies lack contextual understanding [11]. For example, if both “Komodo dragon” and “mice” are equally mentioned within different, user-defined fields of a metadata text sample, traditional term frequency methods may fail to discern that the first refers to the origin of the sample and the latter to the host’s diet. This limitation highlights the need for more sophisticated approaches. Recent advancements in AI have led to the development of robust pre-trained models, particularly Large Language Models (LLMs), which excel at extracting information from diverse and complex metadata across many domains [6,12–14], including the Sequence Read Archive, where LLMs have been shown to robustly extract experimental attributes like cell lines and target genes [15]. Large language models enhance text mining capabilities by effectively understanding context, making them suited for sophisticated tasks like metadata parsing and mining.

We use *MicrobeAtlas* as a testbed for metadata parsing using LLMs. *MicrobeAtlas* is a large, diverse resource, containing millions of metagenomic SRA samples retrieved from NCBI [16]. MicrobeAtlas uses metadata-extracted keywords to assign samples to defined environmental categories based on hard-coded rules. This non-semantic approach can however fail to assign the correct meaning to terms, particularly in the presence of diverse, user-defined metadata fields, leading to ambiguous or even wrong

assignments. Our objective here is to instead leverage general-purpose LLMs for (re-)classification of samples into defined environmental categories, while, simultaneously, retrieving other useful information from the metadata. We classify samples by operational biomes, defined here as broad sample-origin classes used in MicrobeAtlas: ”animal”, “plant”, “water”, “soil”, and “other”. This use of the term biome is tailored for metadata reuse and differs from classical ecological biome concepts or ENVO’s biome class; it is a controlled label for source context, not a climato-vegetation unit. We further assign sub-biomes as concise, human-readable refinements of sample origin (*e.g.,* ”human gut”, “rhizosphere”, “river water”). The tasks given to the LLMs are: I. classification of samples into biomes; II. further classification of samples into sub-biomes; III. extraction of the geographic location of a given sampling site, and IV. extraction of up to eight key terms describing the sample.

We aim to produce high-quality outputs in a cost- and time-efficient manner by systematically evaluating GPT performance across model versions, conditions, and configurations, and by comparing proprietary (OpenAI GPT) and open-weight alternatives. In addition to benchmarking the pipeline with 1,000 hand-curated sample metadata, we release GPT-parsed metadata for over 2 million samples, providing a valuable resource for the community. More broadly, this work fills a critical gap in metadata reuse by offering an approach that bridges the manual curation of small datasets and the unfiltered use of millions of raw, inconsistently annotated records.

Methods

To enhance reproducibility and portability, our entire pipeline—including metadata download and processing, benchmark creation, GPT requests, and output validation—is fully containerized using Docker. Users can deploy via a single Docker image [17] and follow detailed usage steps in the GitHub README.md [18].

Metadata download and processing

We used MicrobeAtlas as a testbed for LLMs metadata extraction. For MicrobeAtlas, the NCBI Sequence Read Archive (SRA) had been searched for DNA sequencing runs with metadata keywords matching "metagenomic", "microb*", "bacteria" or "archaea", and all metadata files for these runs were downloaded in March 2020 [16]. For the present study, all metadata was merged into a single comprehensive metadata file sample.info.gz [19]. This file was then organized on a per-sample basis and sorted into directories named after the last three digits of each sample's name (script: *01_dirs.py*). Environmental-, food-, plant-, species-, and cross-species anatomy-ontologies (ENVO, FOODON, NCBITaxon, PO, UBERON) were retrieved, parsed and subsequently consolidated into a dictionary (script: *fetch_and_join_ontologies.py)*. Metadata files underwent a cleaning process and ontology terms were converted from their numeric representation to their corresponding textual description, utilizing the ontology dictionary we assembled (script: *clean_and_envo_translate.py*). During cleaning, we

removed empty lines and fields containing placeholder texts (*e.g.*, “missing”, “NaN”, “not applicable”), as well as lines that began with “experiment”, which typically include information about wet lab procedures (*e.g.*, sample preparation, sequencing, library preparation, adapter sequences). Log files were maintained in order to track which lines were removed and which ontology codes were converted to their textual counterparts. Coordinates (latitude and longitude) were extracted from the metadata, based on a combination of field names and parseable numeric formats (script: *parse_lat_lon_from_metadata.py*). Occurrence frequencies of major biome-indicating terms within NCBI submission fields were computed (script: *field_distrib_analysis.py*) and the size of metadata files was monitored to ensure they remained manageable for further processing (script: *check_metadata_sizes.py*). (Figure 1; Supplementary Figure 2)

Building a benchmark dataset

To assess the output from the LLMs, we established a benchmark dataset consisting of hand-curated metadata records. This process began with a sub-selection of metadata records from the initial pool of 3.8 million SRA BioSamples and ultimately selecting 1 million samples based on the availability of sufficient metadata. From these selected samples, we extracted identifiers such as DOI, PMID, PMCID, and BioProject accession numbers. Utilizing NCBI’s *Entrez* system, we retrieved referenced publications for each sample record, retaining all samples for which at least one valid PubMed abstract could be retrieved. In instances where multiple publications were associated with a single

sample, we chose the oldest one, assuming it is the most likely primary data reference. However, if the title of the publication contained keywords suggesting it described a protocol, we selected the second oldest publication. The primary objective of this publication assignment strategy was to ensure that each sample selected for the benchmark was linked to a unique abstract, thereby maximizing the likelihood that the samples within the benchmark dataset were sufficiently distinct from one another. This approach helps avoid potential biases that might arise if multiple samples from the same study are selected, which would result in redundant metadata. The resulting selected samples are provided (training_data_pmid_based.csv; *n*=669,108). (Figure 1; Supplementary Figure 2)

The specific task of the hand curation step was to classify samples into five biomes: ‘animal’, ‘soil’, ‘plant’, ‘water’, and ‘other’, based on all preprocessed metadata. The term biome here denotes the operational sample-origin class. It differs from ENVO’s biome concept and is intended for robust, automatable text classification. The choice of these biomes was based on *MicrobeAtlas'* pre-existing two-level ontology, which is what we refer to with “biome” and “sub-biome”. Following *MicrobeAtlas'* choice, rhizosphere samples were curated as *plant* and sediment samples as *water*. Samples with an identifiable origin that did not match any of the five biomes were discarded from the benchmark dataset. Sub-biomes were determined based on more detailed manual examinations of all metadata fields. For animal and plant biomes, sub-biomes correspond to the host taxon and the specific part from which the sample is taken. For example, samples from the animal biome may be classified into the sub-biome “human gut”, while those from the plant biome can be categorized as “olive tree

leaf". Water biome samples were described by their water body source (*e.g.*, river, sea, waste water, ocean, lake). Soil samples were differentiated by soil type (*e.g.*, agricultural, forest, tundra, desert, peatland). Samples with biome category "other" were sorted into the following most frequently encountered sub-biomes: urban, bioreactor, laboratory, feed/food, fungus, air, or other. The sub-biome was assigned by the curator as it was reported in the metadata. For example, if the sample host was described using its scientific or its common name, it was assigned accordingly. (Figure 1; Supplementary Figure 2).

We describe briefly the "keyword-based classifier" that MicrobeAtlas is currently based on. Keywords are extracted from various fields and processed (*e.g.*, lowercasing, removal of special characters, tokenization). Keywords are matched against environment-specific term sets. For example keywords "leaf, banana, tree, crop" match with "plant". When keywords of a sample matched with more than one environment-specific term set (*e.g.*, "leaf, banana, tree, insect"), the sample was labeled as "unknown".

To facilitate a balanced selection of samples across biomes when building the benchmark dataset, we leveraged MicrobeAtlas biome classifications based on the "keyword-based classifier" described above (script: *make_gold_dict.py*). This script retrieves sample metadata, enabling the curator to dynamically assign the most appropriate biome and sub-biome. To allow overrides of biomes or sub-biomes, the script *edit_gold_dict.py* was employed. Additionally, sub-biomes were used in *field_distrib_analysis.py* where they were matched (exact and lenient matches) against the metadata, in order to establish which fields within the metadata were informative

of the sample origin. (Figure 1; Supplementary Figure 2) Finally, the manually curated benchmark dataset consisted of 1,000 samples (200 per biome).

Requests to LLMs

For synchronous requests to LLMs, a pipeline was built (script: *openai_main.py*), structured into five components: (1) setup of a logging system; (2) fetching metadata texts and segmenting them into manageable chunks; (3) executing requests to the OpenAI API (in case of GPT models) or to the deepinfra API (for all other models); (4) an initial parsing of the LLM output, during which sample IDs between input and output are matched. Step (4) allows sample IDs missing from the output or that failed parsing to be requested again. (Supplementary Figure 3A)

For asynchronous interactions with OpenAI, the pipeline involves retrieving the metadata texts, sending batch requests through the API (script: *gpt_async_batch.py*), and retrieving the output up to 24 hours later (script: *gpt_async_fetch_and_save.py*). (Supplementary Figure 3B)

Both synchronous and asynchronous interactions generate a consolidated output file. The name of the output file reports various parameters, such as the number of metadata samples per biome (--*nspb*) randomly selected from the benchmark dataset, and reproducibility via a fixed random seed (--*rs*). Synchronous runs allow for chunking (--*chunking*), where a single request contains the system prompt followed by multiple metadata samples (if chunking is enabled) or a single sample (if disabled). The maximum number of samples fitting into a single chunk depends on chunk size (--

chunksize), which reflects the total number of tokens that can fit into a single request, including the system prompt. We employed a first-fit decreasing binning mechanism to optimize chunk utilization. This method sorts samples by token count in descending order and packs them into the fewest number of chunks possible, ensuring that each chunk is filled close to its token capacity without exceeding it (script: *openai_o2_metadata_processing.py*). Asynchronous requests are never chunked, meaning that one sample metadata per request is sent. Furthermore, users can specify the model (--*model*), the maximum token count (--*maxtokens*), and parameters that determine creativity of chat completion, *e.g.*, temperature (--*temp*), nucleus sampling (--*topp*), frequency penalty (--*freqp*), and presence penalty (--*presp*). The output filename also includes optional text (--*opt_text*) where the user optionally details specifics of the run, the total number of API requests (for synchronous requests), and the timestamp of the output file creation. The designation "batch" within the filename indicates the OpenAI-generated unique ID of an asynchronous request.

To ensure uniformity across all interactions with the LLM, a standardized system prompt was used. This prompt was designed to systematically collect specific information for each sample from its metadata in a structured manner, so as to facilitate a streamlined parsing and analysis of the output. The system prompt was structured as follows:

——

We kindly request your expertise in analyzing the following microbial metagenomic samples from their metadata texts:

- Please deduce the source category for the sample, choosing from 'animal' (including humans), 'plant', 'water', 'soil', or 'other'. Your choices are: 'animal' (incl. human), 'plant', 'water', 'soil', 'other'. Give strictly a concise, single-word label for the sample ID.

- Please infer geographical location where the sample was collected, including the country (NOT the coordinates).

- Extract strictly 5 to 8 keywords descriptive of the sample origin, separated by commas. Put them within curly brackets.

- We seek a brief, up to three-word description of the sample's specific origin. For 'animal' or 'plant' sources, please specify the host and part thereof. For 'water' samples, the type of water body is sought (e.g., lake, brine, sea, waste water, etc). If from 'soil', specify (e.g.: agricultural, desert, forest, etc). If from 'other' specify which (e.g.: urban, laboratory, feed/food, fungus, air, etc).

If information is missing, kindly indicate 'NA'. Please separate all values with three underscores ('____').

An example response: SRS123456____animal____Los Angeles, USA____{medical, bone fracture, infection, collagen, hospital, intensive care, cast, Staphylococcus epidermidis}____human elbow

———

The prompt above was used in all cases except for asynchronous requests and synchronous requests that are used for a direct comparison with asynchronous requests. For these cases, the LLM is prompted to generate the output in a JSON format ("openai_system_prompt_json.txt").

To improve the accuracy of LLM classifications for challenging cases (*e.g.*, rhizosphere and sediment samples), we implemented a tailored prompt

("openai_system_better_prompt.txt"). This prompt instructs the LLM to categorize rhizosphere samples as 'plant' and sediment samples as 'water'. In addition, a human curator was engaged to assess sample classifications using the standard prompt initially and the better prompt in a subsequent round. This approach allowed us to directly compare the performance of the LLM against human curation, particularly for these challenging sample types, and to verify whether targeted prompting improves sample classification. For the comparison of various LLMs (OpenAI *versus* open-weight), the tailored prompt was used.

We conducted a series of tests to compare synchronous and asynchronous request methodologies. For synchronous requests, we examined the impact of enabling chunking versus disabling it, and we assessed how varying the chunk sizes affect the output. Additionally, we evaluate different models, robustness, alongside adjustments in creativity parameters of chat completion, which can influence, among others, term repetition. In scenarios where chunking is disabled, we focus on the effects of tweaking creativity parameters. Our rationale is that, while concatenating multiple metadata texts into a single request might affect output due to term repetition penalties, this effect might differ when each metadata text is sent as a separate request. Similarly, for asynchronous requests, tests to assess performance of different models, as well as their robustness and the effect of tweaking creativity parameters are performed. (Figure 1)

Validation statistics

To validate biome classifications, we parsed the output files to extract the “biome” column and compared each LLM-assigned label with the corresponding curator-assigned biome. We distinguished exact matches, where the two labels are identical, from lenient matches, where one of the five allowed biome labels (“animal”, “plant”, “water”, “soil”, “other”) appears as a complete word but is followed by extra clarifying text (e.g., curator: *animal*; GPT output: *animal (human)*). Lenient matches were counted as correct only for benchmarking purposes, to capture the model’s occasional tendency to append detail under certain parameter settings, and do not represent fuzzy or approximate matching. For comparisons involving repeated sample IDs across runs, we use the McNemar’s test, which is appropriate for paired binary outcomes (True/False). For comparisons across different sample sets, we employ the t-test for independent samples. In both scenarios, a Bonferroni correction is applied to adjust for multiple comparisons. (script: *validate_biomes_subbiomes.py*) (Supplementary Figure 2)

Sub-biome validation is less straightforward, given the free-form data entries in the benchmark dataset and the flexible assignments by the LLM. The “sub-biome” column is extracted from the LLM output and an embedding model is used to generate embeddings for each sample ID from both the LLM sub-biomes and the benchmark dataset sub-biomes (script: *embeddings_from_sb.py*). Unless noted otherwise, sub-biome embeddings were computed with OpenAI *text-embedding-3-small* (1536-D). For model comparisons we also used *Qwen/Qwen3-Embedding-0.6B* (1024-D), *Qwen/Qwen3-Embedding-4B* (2560-D), and *Qwen/Qwen3-Embedding-8B* (4096-D). All embeddings files are compared to calculate cosine similarity (between the LLM- and

the curator-assigned sub-biome embedding), providing a quantitative measure of the accuracy of LLM's sub-biome predictions (script: *validate_biomes_subbiomes.py*). Similarly to biome prediction validation, for runs involving different sample IDs, a t-test for independent samples is used. For runs involving the same sample IDs, comparisons are performed using the paired t-test, which is suitable for comparing cosine similarity scores. (Supplementary Figure 2)

In order to assess the performance of the LLM in accurately determining the location of sample collection, we use latitudes and longitudes extracted from the metadata (script: *parse_lat_lon_from_metadata.py*). For this analysis we include only GPT outputs. The latitudes and longitudes are geo-coded to textual descriptions using the geopy library (script: *coord_to_text.py*). String matches between LLM-predicted locations and the geo-coded locations are recorded. For mismatches, latitude and longitude coordinates for the LLM-predicted locations are obtained using the Google Maps API. This allows us to measure the distance between the LLM-predicted coordinates and those extracted from the metadata. Mismatches are categorized based on the distance between these points into the following categories: less than 100 km, 100-500 km, 500-1000 km, 1000-4000 km, and over 4000 km. Additionally, to determine whether mismatches are due to incorrect LLM predictions or errors in the metadata-derived coordinates, 100 randomly selected samples are manually validated (script: *geo_check.py*). (Supplementary Figure 2)

We conduct a comprehensive analysis of LLM performance by aggregating outputs across various experimental parameters (*e.g.*, chunking, (a)synchronicity of requests, and creativity parameters) (script: *overall_analysis.py*). For this analysis we

include only GPT outputs. The script compiles all GPT output files, merging them into a dataset for comparative analysis against curator-assigned biomes (benchmark dataset). The lenient match accuracy is calculated to assess the accuracy of biome predictions both overall and within specific biome categories. The script also compares the GPT-predicted classifications with MicrobeAtlas's previous biome predictions (based on the "keyword-based classifier") to determine whether GPT has improved our sample classification. (Supplementary Figure 2)

To contextualize the accuracy of LLM-based classification, we conducted a comparative assessment between LLM and human performance. For this test we only included GPT outputs as representative of LLMs. We randomly selected 250 samples from the benchmark dataset and provided the corresponding metadata to a trained molecular biologist with no prior exposure to this project. The human annotator was asked to assign biomes and sub-biomes to the samples using the same system prompt initially used for GPT (see: "Requests to LLMs" section). In a second round, both the human annotator and GPT were presented with an improved version of the prompt. The revised prompt included a clarifying instruction: "Please note that rhizosphere samples should be categorized as 'plant' and sediment samples as 'water'." This addition was made to assess whether explicit guidance improves classification accuracy for ambiguous cases. We evaluated and compared the performance of both classifiers-- GPT and human-- under each prompt condition (standard *vs* 'better'). This experiment allowed us to understand how well GPT performs relative to an uninitiated but scientifically literate human, and whether targeted prompting can effectively steer, not only human, but also machine classification.

Results

To provide an overview of the analyses presented below, we first benchmarked GPT-based classification of microbiome samples against a manually curated dataset and a keyword-based reference system. GPT achieved markedly higher biome-level accuracy, precision, and F1-scores than the keyword-based approach, with performance approaching that of a trained human annotator. We then systematically evaluated factors influencing model performance, including prompt design, model version, output format, creativity parameters, and computational strategies such as chunking and asynchronous querying. These analyses revealed that simple prompt refinements improved accuracy, while large chunk sizes (*i.e.*, multiple sample metadata per request) reduced it. Across GPT model versions, GPT-4 and later models provided slightly higher sub-biome annotations quality, particularly when using structured JSON output. We also assessed GPT's ability to infer sample geographic origins, finding high agreement (~96%) between GPT-extracted and metadata-derived locations. Finally, we extended our analysis beyond OpenAI models, showing that recent open-weight models (*e.g.*, *Qwen3/Qwen-80B-A3B-Instruct*) achieve comparable accuracy at substantially lower cost, underscoring that both proprietary and open-weight LLMs can support large-scale, FAIR-aligned metadata re-annotation. The following sections provide detailed results and methodological context for each of these analyses, including, at last, an examination of which metadata fields most frequently capture sample origin information. All accuracies reported below refer to the operational biome

labels and sub-biome labels defined in the Introduction and in Methods, not classical ecological biomes.

GPT overall performance

When testing the classification performance of our LLM-based classifier (GPT) on our manually curated benchmark dataset (*n*=1,000 samples, 200 per biome), using a carefully crafted prompt (see Methods), we found that the model reached an average biome classification accuracy of 80.6% (precision: 82.0%, F1-score: 80.34). This performance was robust to variations in GPT run modes and parameters (n=105; accuracy: 76.0% to 84.1%; precision: 77.1% to 85.8%; F1-score: 76.4% to 84.0%) and was achieved without any example-driven fine-tuning of the model. When examined separately by biome, the accuracy shows variability. ‘Soil’ and ‘animal’ biome samples show the highest accuracy with accuracy rates of 94.6% and 93.8%, respectively. In contrast, ‘plant’, ‘water’, and ‘other’ biome samples exhibit lower accuracy rates (67.3%, 79.9%, and 67.2%, respectively) (Figure 2).

Importantly, GPT markedly outperformed MicrobeAtlas’s current, keyword-based classification system (“keyword-based classifier”) in terms of biome prediction accuracy (80.6% vs. 62.5%), precision (81.9% vs. 67.3%) and F1-scores (80.3% vs. 63.5%). We observed the largest performance gain for animal samples (accuracy: 93.8% vs 53.2%, precision: 87.8% vs. 82.4%; F1-score: 90.7% vs. 64.7%), which the keyword-based classifier frequently bins into 'unknown'. Both methods struggle with plant samples, which they often misclassified as soil (Figure 2A-B). Whereas GPT most often misclassified them as ‘soil’ (27.0%), the keyword-based classifier repartitioned the

misclassified samples into either ‘soil’ (16.8%) or ‘other’ (17.8%). Overall, GPT predictions resulted in a more balanced distribution across biomes and a better alignment with expected frequencies, as indicated by Cohen’s kappa values (GPT: 0.760; keyword-based classifier: 0.530). (Figure 2)

Analysis of GPT misclassified samples

The overall 19.4% of incorrect predictions led us to investigate whether specific samples are consistently misclassified in predictable ways. Detailed analysis of all GPT misclassifications confirms a subset of samples with significant biases among misclassified biomes (chi-squared: 7482; p-value < 0.001). A large fraction of misclassified ‘animal’ samples (53.0%) are erroneously predicted as ‘water’. In contrast, the majority of misclassifications within ‘plant’ (83.9%) and ‘water’ samples (59.0%) are ‘soil’ (Supplementary Figure 4).

The distribution of misclassifications per sample is right-skewed (skewness: 1.34; kurtosis: 0.27), indicating that most samples have relatively few misclassifications compared to a small subset with disproportionally high values (Supplementary Figure 5). Notably, 21 samples (5.3% of the total) fall at or above the 95th percentile, with 90 or more misclassifications, which is consistent with a right-skewed distribution. Across 105 independent classification runs using different parameter settings, the average number of misclassifications per run is 21.7 (SD: 29.9). Half of the samples are misclassified 4 times or fewer, and three-quarters have no more

than 34 misclassifications. The maximum observed was 97, indicating that some samples were misclassified in almost every run, highlighting extreme cases where GPT predictions consistently disagreed with the curator-assigned biome. (Supplementary Figure 5) A detailed examination of such cases includes: a bioreactor digester wastewater sample (SRS994677), a mock community sample (SRS2217033), and two rhizosphere samples (SRS5304049, SRS942824). The bioreactor digester wastewater sample, categorized as ‘other’ by the curator, was incorrectly assigned to ‘water’ by GPT in 89 out of 97 instances. The other eight were correctly assigned as ‘other’. The mock community from a gut microbiome study should have been categorized as ‘other’ due to its laboratory nature, while it was erroneously assigned to ‘animal’ by GPT in 90 out of 93 instances. The other three were correctly assigned. The two rhizosphere samples were misclassified as ‘soil’ by GPT in all but one case.

Human *versus* GPT classification accuracy

To evaluate how close GPT's performance comes to the upper limit of achievable accuracy, we compared it to that of a human annotator. A trained molecular biologist, with no prior exposure to the project, was given the same prompt instructions as GPT and was asked to classify sample biomes and sub-biomes. While against the benchmark dataset, GPT achieved an accuracy of 79.76% (n=499; SD=40.0), the human annotator reached 78.0% (n=250; SD=42.0).

In a second round, both GPT and the human were given the same set of samples to classify, this time using an improved prompt (here referred to as “better prompt”).

The only change was an added instruction: "Please note that rhizosphere samples should be categorized as 'plant', and sediment samples as 'water'." With the better prompt, both classifiers showed improved performance. GPT's accuracy increased to 83.17% (SD=37.0), while the human annotator reached 88.0% (SD=33.0).

There was an improvement in accuracy between the human's first attempt (with the initial prompt) and the human's second attempt (better prompt) (adj p-value≤0.001). No significantly different performances were detected for the sub-biome classification, neither between GPT and the human, nor between prompt versions (adj p-value=1).

Resource-saving pipeline settings and their impact on GPT output quality

One way to reduce GPT costs is by minimizing the token count submitted to the API, which we achieved by eliminating fields that are empty or have non-informative place-holders such as “NaN” and “unknown”. While the input had to be lengthened in some areas (for example, Environment Ontology *i.e.*, ENVO codes needed to be converted from numeric IDs to their English text counterparts), we overall achieved a 35% decrease in the size of the input data. Specifically, for the GPT-3.5-turbo-0125 model, which as of October 2025 charged $0.5 per million input tokens, a 35% reduction in tokens results in the cost for processing 2 million samples dropping from approximately $522 to $340.

Instead of sending metadata for individual samples in separate requests, we explored grouping multiple samples' metadata into single requests (chunking) to reduce the token overhead of repeatedly specifying the input/system prompt. We compared the cost-effectiveness of chunking versus no chunking, and evaluated whether chunking affected performance. We evaluated both biome as well as sub-biome prediction. Our initial findings show no significant performance difference between mild chunking *(i.e.*: chunk size 3000: median of 5, mean of 6, maximum of 17 samples per request) and no chunking (one sample per request). This was consistent across both biome prediction (adj *p*-value=0.56) and sub-biome prediction (adj *p*-value=1) (Figure 3B)

Biome prediction starts to slightly worsen when using larger chunk sizes: biome accuracies for chunk sizes of 2000, 3000, 4000, 5000, and 6000 tokens per chunk (median sample counts of 3, 5, 7, 9, and 15, respectively) were 78.76%, 78.2%, 77.2%, 73.85%, and 72.15% (Figure 3A). The noticeable drop in accuracy starting with chunk size 6000 was significant compared to chunk size 2000 (adj p-value=0.03) (Figure 3B). Additionally, cosine similarity between benchmark dataset sub-biomes and predicted sub-biomes starts to lower significantly with chunk size 5000 (average cosine similarity: 0.46) compared to chunk size 2000 (average cosine similarity: 0.52) (adj p-value=0.00115), indicating that GPT is worse at predicting sub-biomes at higher chunk sizes (Figure 3B).

GPT models & output formats

We compared the performance of three OpenAI models: *GPT-3.5-turbo-0125*, *GPT-3.5-turbo-1106* and *GPT-4-0613* by sending synchronous requests to each model. The accuracy of biome predictions is found consistent across all models, with no statistically significant differences (adj p-value $\geq$ 0.05) (Figure 4). However, we observed differences in the cosine similarity between benchmark dataset sub-biomes and predicted sub-biomes. Specifically, *GPT-4-0613* demonstrated a slightly yet significantly higher cosine similarity compared to both *GPT-3.5-turbo-0125* (adj p-value $\leq$ 0.001) and *GPT-3.5-turbo-1106* (adj p-value $\leq$ 0.001) (Figure 4B).

To assess the impact of different output formatting on GPT's performance, we designed two system prompts that instruct GPT to format its output either inline (using double underscores to separate answers for each sample), or in JSON format (by stating it in the prompt and by specifying the 'format' parameter in the API request). While biome prediction accuracy remained constant across both formats, we observed an improvement in sub-biome cosine similarity when responses were formatted in JSON (adj p-value < 0.001). (Figure 5)

The effect of tweaking GPT creativity parameters

Parameters such as temperature, nucleus sampling, and penalty settings influence how deterministic or diverse the outputs of LLMs are, and thus could potentially affect prediction. Adjustments to temperature, nucleus sampling, or presence-penalty

settings did not impact the prediction accuracy for biome or sub-biome categories (adj p-value ≥ 0.05) (Supplementary Figure 6). Accuracy consistently dropped with increasing frequency penalty, from an initial 79.2% (freqp 0.0) to a minimum of 64.3% (freqp 2.0). This decline was significant (adj p-value < 0.001) (Figure 6). However, when the more lenient criterion for string-matching of the GPT output with the benchmark dataset biome was applied (*i.e.*, “lenient match”), the impact of increased frequency penalty on biome accuracy was mitigated (freqp 0.0: 79.16%; freqp 2.0: 78.31%) (Figure 6A). By examining GPT answers, it was clear that a higher frequency penalty led GPT to deviate from strictly adhering to the prompt instructions "Answer with exactly one word from the following...". Instead, the model tended to provide unsolicited information *e.g.*, instead of providing “animal” for an answer, it provided “animal (incl. human)”. In fact, biome accuracy under the lenient matching condition remained unaltered with differing frequency penalties.

Remarkably, significant differences across creativity parameters were detected only in the case of chunked requests. Without chunking, neither synchronous requests nor asynchronous requests showed significant differences (Supplementary Figure 6). This indicates that the tweaking of creativity parameters, specifically frequency penalty, affects the output only when multiple samples' metadata is submitted in a single request.

GPT synchronous and asynchronous requests

To assess the reliability of different querying strategies in large-scale automated analyses, we compared the robustness of synchronous (without chunking) and asynchronous requests. We conducted 10 replicate tests for each: 500 randomly selected metadata samples were sent individually to 10 synchronous runs or 10 asynchronous runs (replicates 1 to 10). For synchronous requests, the biome accuracy (lenient match) did not vary (range=78.2-80.8), and neither did the sub-biome cosine similarity (range=0.49-0.51) (Supplementary Figure 7A,D). Asynchronous requests demonstrated similar consistency for biomes accuracy (lenient match) (range=81.8-83.2), and no significant variations across different runs for sub-biome predictions (range=0.56-0.58) (adj p-value = 1) (Supplementary Figure 7B,E). Note that the higher accuracies for asynchronous requests here are due to the different output format used for each.

We evaluated the performance differences between synchronous and asynchronous requests (n=6 replicates each) in terms of both biome and sub-biome prediction accuracies, keeping all parameters equal (including the output format). Our analysis revealed that whether requests are sent synchronously or asynchronously does not correlate with a better prediction of biomes nor sub-biomes (adj p-value=1) (Supplementary Figure 7C,F).

Geographic location prediction performance

During the production run, we instructed *GPT-3.5-turbo-0125* to extract the geographical location where each sample was collected, including the country (in textual format, *i.e.,* not in latitude/longitude coordinates). We measure the performance by comparing the output with textual descriptions derived directly from lat/lon coordinates parsed from the metadata. Among 990,172 samples analyzed (of over 2M), 95.32% matched the geo-coded location. The distribution of the matching and mismatching samples can be visualized on a global map (Figure 7).

To further analyze the 4.68% mismatches (n=46,311), we obtained the actual coordinates for the GPT-predicted locations using the Google Maps API and assessed the distance between these and the metadata-derived coordinates. The distances ranged from 0.6 km to 19,856 km (1st quartile: 244.7 km, 3rd quartile: 10,294.4 km). The distribution of the mismatching samples color-coded by distance categories can be visualized on a global map (Supplementary Figure 8). For a focused investigation, a subset of 100 misclassified samples, randomly picked and with a discrepancy above 1000 km between the GPT-predicted locations and the metadata-derived coordinates, were examined manually. This analysis revealed that:

- In half of the cases GPT correctly predicted the location, while the lat/lon coordinates parsed from the metadata were incorrect. This happened for various reasons: the coordinates in the metadata were either completely wrong (30%), lat/lon were swapped (7%), had wrong longitudinal signs (negative (34%) or positive (5%)), had wrong latitudinal signs (negative, 9%), a wrong latitude (5%), or the coordinates

pointed at the academic institution of the authors rather than to the location where the sample was collected (7%);

- In 27% of the cases both GPT and coordinates pointed at the right geographic location, but the name given by GPT did not textually match with the geographic location from the coordinates (*e.g.*, McMurdo station vs Antarctica). This often happened with coastal samples of the United States, where GPT returns "United States".

- In 16% of the cases the coordinates pointed to the right location, but GPT didn't extract it correctly. This happened because GPT mistook the institute location for the sampling location (41%), the coordinates pointed to a water body or an island (*e.g.*, "Hawaii") while GPT predicted the location after the country of belonging of that water body (*e.g.*, "United States") (18%), or the metadata did not clearly mention the location in text format (41%).;

- In 7% of the cases metadata-extracted coordinates and GPT geolocation did not match because neither information was retrievable from the metadata as it was ambiguous or absent.

How does GPT compare with non-OpenAI models?

To evaluate whether OpenAI's GPT models perform comparably to other state-of-the-art large language models, we benchmarked *GPT-3.5-turbo-1106*, *GPT-4.1*, and *GPT-5-mini* against a representative set of open-weight models, including *meta-llama/Meta-Llama-3.1-8B-Instruct*, *Qwen/Qwen3-Next-80B-A3B-Instruct,* *microsoft-phi-4,* and

Mistral-Nemo-Instruct-2407. All models were prompted identically using the optimized "better prompt" (see Methods), ensuring a fair comparison across models.

Biome accuracy was similar across models, with several models forming a single *post hoc* group (Figure 8A). *Qwen3-Next-80B-A3B-Instruc*t achieved the highest observed accuracy for exact/lenient biome agreement (86.37%), very slightly above GPT-5-mini (85.57%), *GPT-4.1* (84.97%), *GPT-3.5-turbo-1106* (84.77%), and *microsoft-phi-4* (83.37%); however, these differences did not reach statistical significance after *post hoc* correction (letters "a" in Figure 8A). In contrast, *Mistral-Nemo-Instruct-2407* (76.95%) and especially *Meta-Llama-3.1-8B-Instruct* (69.34%) lagged behind the other models. (Figure 8A)

For sub-biome similarity, results were broadly consistent across embedding models—the LLM ranking was preserved and differences within a given LLM were modest (Figure 8B). Using *text-embedding-3-small* as the embedding model, *GPT-4.1* and *Qwen3-Next-80B-A3B-Instruct* achieved the top scores (0.63 and 0.62, respectively), followed by *GPT-5-mini* and *microsoft-phi-4* (both 0.61) and *GPT-3.5-turbo-1106* (0.60). *Mistral-Nemo-Instruct-2407* (0.58) and *Meta-Llama-3.1-8B-Instruct* (0.57) had the lowest mean scores. Pairwise testing reflected these gaps: *Mistral-Nemo-Instruct-2407* was significantly below *GPT-4.1* (adj p-value = 0.00128), and *Meta-Llama-3.1-8B-Instruct* was significantly below *Qwen3-Next-80B-A3B-Instruct* (adj p-value < 0.001) and *microsoft-phi-4* (adj p-value = 0.03816).

With respect to embedding model choice, pooling across LLMs, *Qwen* embedding models outperformed *text-embedding-3-small* (adj p-value < 0.001). *Qwen3-Embedding-8B* resulted in the highest average similarity and was not statistically

different from *Qwen3-Embedding-4B* across models (adj p-value > 0.05), while *Qwen3-Embedding-0.6B* was significantly lower than both *Qwen3-Embedding-8B* and *Qwen3-Embedding-4B* in several model comparisons (*e.g.*, *Meta-Llama-3.1-8B-Instruct* and *microsoft-phi-4*; adj p-value < 0.05). Notably, the embedding models used in these analyses differed in dimensionality: *text-embedding-3-small* produces 1,536-dimensional vectors, whereas *Qwen3-Embedding-0.6B*, *Qwen3-Embedding-4B*, and *Qwen3-Embedding-8B* produce 1,024-, 2,560-, and 4,096-dimensional vectors, respectively. Together, these results indicate that embedding choice substantially raises or lowers the absolute cosine values (*Qwen* embeddings score notably higher than *text-embedding-3-small* (adj p-value < 0.001)), but it does not alter the relative LLM ranking: *GPT-4.1* and *Qwen3-Next-80B-A3B-Instruct* remain at the top, with *Meta-Llama-3.1-8B-Instruct* and *Mistral-Nemo-Instruct-2407* at the bottom. At the biome level, OpenAI's GPT models perform strongly, but several non-OpenAI models, particularly *Qwen3-Next-80B-A3B-Instruct* and, for embeddings, *Qwen3-Embedding-8B* or *Qwen3-Embedding-4B*, achieve comparable or better accuracy, respectively, indicating that high-quality biome and sub-biome annotation can be achieved with both proprietary and open-weight architectures, expanding options for large-scale ecological metadata re-annotation.

In addition to comparable performance, open-weight models offer a substantial cost advantage. Based on current pricing (input/output tokens per million), their costs are typically 10–50 times lower than those of proprietary GPT models. For example, *Qwen3-Next-80B-A3B-Instruct*, *Meta-Llama-3.1-8B-Instruct*, and *microsoft-phi-4* are priced at approximately $0.03–0.14 per million input tokens and $0.05–1.10 per million

output tokens, compared with $1.00-2.00 for input and $2.00-8.00 for output tokens for OpenAI's *GPT-3.5-turbo-1106* and *GPT-4.1-mini*. Although the open-weight models themselves are free of cost, the costs mentioned here reflect fees from third-party hosting platforms (*e.g.*, DeepInfra) used to access the models, since running large open-weight models locally requires dedicated GPU hardware.

Informative metadata fields

Not all metadata fields contain information about sample origins, but as field names are not always standardized, it can be difficult to know *a priori* which fields to parse and focus on. By programmatically comparing the metadata of 1,000 samples to their curator-assigned sub-biomes, we determined which fields typically report the sample origin. We found that the sample origin was documented in 67 distinct fields when considering full matches (*e.g.*, "cow rumen") and in 127 distinct fields when considering lenient matches (*e.g.*, "cow" or "rumen"). On average, the full origin of a sample is found in 1.36 fields per sample (SD: 1.67), and at least part of the sample origin is reported in 3.0 fields (SD: 2.2) per sample, indicating a modest variability in how sample origins are documented across different metadata fields.

Some fields are more commonly used than others to report the origin of the sample. The fields most frequently containing sample origin information are: "study_STUDY_ABSTRACT" (267 instances), "sample_SCIENTIFIC_NAME" (225 instances), "study_STUDY_TITLE" (212 instances), "sample_isolation_source" (113 instances), and "study_STUDY_DESCRIPTION" (53 instances). Despite their relevance

for mentioning sample origins, the fields "study_STUDY_ABSTRACT" and "study_STUDY_DESCRIPTION" are more challenging to parse given their average word count (72.8 and 144.8, respectively) compared to "sample_SCIENTIFIC_NAME" (2.12), "sample_isolation_source" (2.9), and "study_STUDY_TITLE" (8.9).

The variability in field usage does not seem to be arbitrary, but appears influenced by the biome associated with the sample. For example, sample origin is more likely to be found under the field "sample_host" in the case of animal and plant samples, while the fields "sample_env_biome" and "sample_env_feature" are more likely to contain useful information in the case of soil, water and "other" samples. This demonstrates that certain fields are preferentially selected to report sample origin, depending on the sample biome. (Supplementary Figure 9)

Discussion

A central goal of data repositories is to enable the effective secondary use of research data, potentially leading to new discoveries beyond the original scope of the study. In practice, however, most datasets are rarely re-analyzed by researchers outside the submitting laboratory. A major barrier to reusability is poor metadata, often incomplete, inconsistent, or non-standardized, which limits automated parsing and interpretation. As a result, the full potential of many datasets remains untapped. Recent systematic assessments confirm this trend: nearly half of microbiome studies fail to meet minimum metadata reporting standards, despite sharing sequence data,

underscoring the persistent lack of standardization and interoperability in the field [20]. Two strategies come to mind to address this issue: (1) enforcing more rigorous reporting standards at the time of submission, and (2) retroactively improving metadata quality using computational tools. While ongoing efforts have improved metadata submission guidelines, these changes are difficult to enforce consistently and do not address the vast amount of legacy data already in repositories. In contrast, large language models (LLMs) offer a flexible and scalable means to retrospectively annotate and standardize metadata, even when it is noisy or poorly structured. In this study, we evaluated the use of large language models (LLMs) for metadata re-annotation of environmental sequencing samples. To this scope, we used proprietary OpenAI models as well as several open-weight models, and we compared their performance.

When evaluating LLM performance in classifying environmental sequencing samples, we found that it consistently outperformed a traditional keyword-based classifier. Unlike the latter, which relies on static keyword matching and extensive hard-coded white lists, LLMs leverage context and semantics to disambiguate terms based on their usage and surrounding information within the metadata. Importantly, our approach achieved this performance without any fine-tuning, demonstrating that general-purpose text generation models can perform complex metadata re-annotation tasks effectively. This contrasts with task-specific approaches such as *ChIP-GPT*, where a model was fine-tuned on curated examples to extract metadata from SRA records with comparable accuracy [15].

To contextualize model performance, we compared GPT's classifications with those of a trained molecular biologist unfamiliar with the project. Both were presented

with the same metadata and prompt, and their classifications were evaluated against the benchmark. Under standard conditions, GPT and the human annotator achieved similar accuracy. When given a clearer, more explicit prompt, both improved. This demonstrates not only GPT's capacity to perform at a near-expert level for this classification task, but also highlights the importance of precise instructions—an aspect that benefits both human and machine annotators. These findings align with observations by Sundaram *et al.* (2025), who showed that prompts incorporating explicit metadata schema information markedly improved LLM output consistency and completeness [21].

However, LLMs are not without their limitations. One challenge is the need to translate ontology accession codes—commonly found in metadata—into human-readable terms, as LLMs do not interpret these codes natively. This step of ontology translation directly supports the *Interoperability* component of FAIR by aligning textual metadata with standardized vocabularies such as ENVO and FOODON [21]. This approach parallels the template-augmented prompting approach of Sundaram *et al.* (2025), where domain-specific schema and structure guide LLMs toward outputs that are consistent with metadata standards and reduce semantic inconsistencies [21]. Secondly, LLMs do not always "read between the lines". For instance, if a sample is part of an animal study but correctly described as a mock community, the LLM may still classify it as 'animal' rather than 'other'. Prompt adherence is also imperfect: if the metadata points frequently at the term 'soil', the LLM may default to assigning that biome, regardless of more nuanced cues. This was especially evident for rhizosphere and sediment samples, which were major contributors to biome misclassification.

These cases illustrate a broader challenge: sample classification in environmental data is often inherently ambiguous. A sediment sample taken from a coastal area, for example, could reasonably belong to either 'soil' or 'water' depending on phrasing. Similar issues affected the keyword-based classifier, which also showed bias in such edge cases. These examples underscore the difficulty of resolving ambiguity in free-text metadata, regardless of the method applied.

Taken together, the approximately 20% misclassification rate observed with LLMs likely reflects an upper limit of LLM performance, rather than a true failure rate. Given the unavoidable ambiguity and challenges in re-annotating diverse samples against a necessarily limited and simplified classification scheme, even a second human curator might not agree with the benchmark labels. This suggests that even LLMs operating at human level may not be sufficient for perfect categorization, especially in domains like environmental sampling where a certain amount of overlap is to be expected. This is consistent with findings from other domains, where LLMs can extract complex biological attributes with high accuracy yet still require human verification and post-processing to correct misapplied or ambiguous entries [22].

An alternative to direct classification is to prompt the model to generate a brief description of the sample's origin and then derive embeddings from these summaries. These embeddings can be compared to those generated from curator-provided descriptions. This approach allows for more flexible sample grouping: even if a sample is misclassified at the biome level, its sub-biome description—*e.g.*, "mock community"—may still position it correctly in embedding space alongside similar samples. In this study, we used such comparisons primarily to validate the LLM output

via cosine similarity scores, but the same method could also support clustering or reclassification of ambiguous samples.

Tweaking GPT 'creativity parameters' such as temperature, nucleus sampling, and presence penalty had no impact on biome or sub-biome prediction accuracy. However, increasing the frequency penalty led to reduced biome accuracy—but only when multiple samples were included in a single request (*i.e.*, chunking). This effect disappeared when samples were submitted individually. A likely explanation is that repeated use of a term like 'animal' within a chunk triggers the penalty, prompting GPT to vary its wording (*e.g.*, returning "animal (human)" instead). Supporting this, the accuracy drop vanished when using lenient string matching. Interestingly, rather than avoiding penalized terms altogether, GPT tended to add detail to them. Aside from frequency penalty effects during chunking, no other creativity parameter significantly affected classification performance.

We also assessed the impact of request mode, model version, and output format on GPT's performance. As expected, there was no difference between synchronous and asynchronous requests, since both use the same underlying model. Given this, asynchronous requests are currently preferable, as they are easier to manage at scale and are more cost-effective. Model choice, however, did influence output: *GPT-4-0613* outperformed both *GPT-3.5-turbo-1106* and *GPT-3.5-turbo-0125* in sub-biome prediction. Output format also had an effect. While biome predictions were unchanged, sub-biome accuracy improved significantly when using JSON rather than inline formatting. This likely reflects the benefit of structured, machine-readable output, which reduces ambiguity and promotes consistent generation. Although both formats

yielded similar recall, the structured format may help the model better focus on the task, possibly by encouraging more consistent output or reducing ambiguity during generation. Ensuring consistent and machine-readable output is critical for scalable downstream analysis. Similar challenges have been documented in other LLM-based workflows, for example, in morphological extraction tasks where unexpected HTML or LaTeX formatting required repeated re-queries [22], and in systems like ChIP-GPT where outputs sometimes include unsolicited content or deviate from strict formatting [15].

As the use of GPT's API is invoiced based on the number of input and output tokens, we attempted to reduce costs by (1) cleaning away uninformative fields from the metadata and (2) sending multiple samples' metadata under the same request (*i.e.*, chunking). The preliminary cleaning of the metadata proved valuable, reducing input token size by 35%. However, we observed diminishing returns at larger chunk sizes. We hypothesized that sending too many samples at once might "confuse" the model and tested the limit of this strategy. In our case performance declined significantly at 5000 tokens per request (roughly nine samples), while remaining stable at 2000 tokens (about three samples). A minor decline was visible at 4,000 tokens (seven samples), but not statistically significant. Although we did not test chunking with asynchronous requests, we have no reason to believe the results would differ. It is important to note that these thresholds may vary for different metadata types.

Our analysis of 1000 samples demonstrated that the sample origin typically appears in 1 to 3 fields but spans across no fewer than 127 distinct field types, underscoring the immense variability in how metadata is reported. The best field for inferring the broad origin of a sample is the study_STUDY_ABSTRACT field, which is

also the most verbose and thus challenging to process. The sample_SCIENTIFIC_NAME field often reflects the sample origin in host-associated metagenomic samples and is more concise in length. Overall, the usefulness of specific fields varies across biomes, suggesting that a one-size-fits-all selection of metadata fields would not perform equally well across all sample types. This variability is likely influenced not only by user practices but also by differences in the BioSample package system used for submission. Historical or package-specific requirements may have further contributed to the uneven distribution of field usage across biomes. This highlights the persistent challenges in standardizing metadata extraction, despite ongoing efforts to regulate SRA submissions.

We also used GPT to extract the geographic location of each sample's collection site. This was useful in cases where latitude/longitude coordinates were missing, incorrect, or inconsistently formatted. Issues included swapped coordinates, decimal errors, or identical shifts across samples of the same study due to submitters using the drag-copy function in Excel. In many such cases, GPT successfully inferred the sampling country or locality based on free-text metadata, achieving 95% accuracy. Among mismatches, about half were due to incorrect metadata coordinates, while a quarter were semantic mismatches (*e.g.*, "Antarctica" vs. "McMurdo Station"). In a fifth of the cases, GPT misidentified the location, either due to confusing institution names with sampling sites or due to vague metadata. A small fraction of samples had no retrievable geographic information at all.

To determine whether our findings were specific to proprietary GPT models or broadly applicable across architectures, we compared OpenAI models with several

open-weight LLMs. This comparison is relevant for reproducibility, cost, and long-term accessibility, as open-weight models can be self-hosted and remain usable independent of commercial APIs. Performance was comparable across models: OpenAI's GPTs remained strong, but the open-weight *Qwen3-Next-80B-A3B-Instruct* achieved similar accuracy for biome and sub-biome annotation. Importantly, these open-weight models achieved comparable performance at a fraction of the cost—typically 10–50 times lower than proprietary GPT models—further supporting their scalability for large-scale annotation. Embedding choice was also relevant, with three Qwen embedding models (0.6B, 4B, and 8B) outperforming OpenAI's *text-embedding-3-small*. This is unlikely due to higher dimensionality alone, as the 1,024-dimensional Qwen3-Embedding-0.6B outperformed the 1,536-dimensional OpenAI's *text-embedding-3-small* model. Importantly, within the Qwen family, performance did not scale monotonically with size: the 0.6B model consistently exceeded the 4B, whereas the 8B was best overall, suggesting that training objectives, corpus alignment, and potentially lower overfitting in smaller models, rather than embedding dimensionality, are the main drivers of performance. Overall these results suggest that open-weight LLMs have reached a maturity level that enables reliable, cost-effective large-scale metadata re-annotation while improving reproducibility and sustainability [5,22].

While our analyses demonstrate the utility of LLMs for metadata re-annotation, the present study focuses on a limited subset of metadata fields—specifically those related to sample origin, including *biome*, *sub-biome*, and geographic location. These fields were selected because they are among the most informative yet inconsistently reported attributes in microbiome studies, and because they serve as strong indicators

of sample context, a key determinant of ecological interpretation. Other metadata categories, such as sequencing methods, host characteristics, or experimental conditions, were not systematically evaluated here. Their structure, controlled vocabularies, and interdependencies may introduce different types of ambiguity or standardization challenges. Accordingly, the current findings should be viewed as a proof of concept demonstrating that LLMs can successfully handle complex, text-rich metadata fields. Extending these approaches to other metadata domains will require additional benchmarking and potentially more specialized prompt designs or schema-aware models.

In conclusion, our results indicate that LLMs already operate at near-human accuracy for structured re-annotation tasks—at least in relatively structured tasks like microbiome sample origin classification. Errors still occur, partly due to model limitations and the inherent ambiguity of real-world samples, and occasionally due to parsing issues, an observation consistent with reports that complex metadata descriptions, schema limitations, and computational constraints continue to challenge full automation [5]. More straightforward technical constraints include rate limits imposed by commercial APIs and token limits per request. However, open-weight architectures alleviate many of these constraints and, as demonstrated in this study, now demonstrate performance equivalent to proprietary models for ecological metadata annotation. They also offer greater scalability and flexibility, since the models themselves are freely available and can be run locally or on institutional hardware. Additionally, open-weight models can be run repeatedly to build consensus across predictions, increasing confidence in the annotations. In practice, most users

currently rely on third-party platforms such as DeepInfra or Hugging Face to access sufficient GPU resources, which may still incur computational costs, but these are substantially lower than those associated with proprietary model providers. This balance between openness, affordability, and performance positions open-weight models as a sustainable option for large-scale metadata re-annotation. Recent work applying LLMs to BioSample metadata has shown similar benefits, with model-assisted extraction markedly improving the findability and reusability of experimental records [5]. Together, these findings demonstrate that LLMs-including open-weight architecture- provide a practical and scalable foundation for FAIR-compliant metadata re-annotation in microbiome research, improving data interoperability and enabling future discoveries.

Data availability

All data necessary to run the containers can be downloaded from Zenodo [19]. All outputs and results of biome and sub-biome validation and all results from the comparisons performed (biome_subbiome_results*.csv, and biome_subbiome_stats*.csv, respectively) are available under the same Zenodo link. All output from the production run (>2M samples) is available under the "production" directory, also available under the same Zenodo link. Files descriptions are provided. Interactive map figures are downloadable from Zenodo [23,24].

Availability of Source Code and Requirements

Project name: metadata_mining

Project homepage: https://github.com/GaioTransposon/metadata_mining

Operating system: Linux, macOS, and Windows (via Docker)

Programming language: Python

Other requirements: Docker (gaiotransposon/metadmin) (all scripts and dependencies are packaged in a Docker image; see repository README for execution instructions).

Acknowledgements

We are grateful to the members of the von Mering lab—past and present—for their valuable input and stimulating discussions that shaped the development of this manuscript, in particular Marija Dmitrijeva, João Rodrigues, Tao Fang, Tülay Karakulak, Damian Szklarczyk, Qingyao Huang, Radja Hachilif, and Maria Heimlicher. We extend special thanks to George Hausmann for his critical role in annotating a subset of benchmark samples. His independent annotations provided an important reference point for evaluating the upper bound of classification performance and greatly contributed to the interpretation of results. D.G., D.P. and C.v.M. were supported by the Swiss National Science Foundation through its National Competence Center for Research "Microbiomes" (grant number 310030_192567). L.M., N.N., E.P.-M.-M, and M.E.P. were supported by an SNSF project grant to C.v.M. (grant 310030_192569).

Competing interests

The authors declare that they have no competing interests.

References

1. ENA Browser. https://www.ebi.ac.uk/ena/browser/about/statistics Accessed 2025 Mar 26.

2. Caliskan A, Dangwal S, Dandekar T. Metadata integrity in bioinformatics: Bridging the gap between data and knowledge. *Comput Struct Biotechnol J*. Elsevier; 2023; doi: https://doi.org/10.1016/j.csbj.2023.10.006.

3. Piwowar HA, Vision TJ. Data reuse and the open data citation advantage. *PeerJ*. PeerJ Inc.; 2013; doi: https://doi.org/10.7717/peerj.175.

4. Park MS, Park H. An examination of metadata practices for research data reuse: Characteristics and predictive probability of metadata elements. *Malays J Libr Inf Sci*. 2019; doi: https://doi.org/10.22452/mjlis.vol24no3.4.

5. Ikeda S, Zou Z, Bono H, Moriya Y, Kawashima S, Katayama T, et al.. Extraction of biological terms using large language models enhances the usability of metadata in the BioSample database. *GigaScience*. Oxford University Press; 2025; doi: https://doi.org/10.1093/gigascience/giaf070.

6. Sundaram SS, Musen MA. Making Metadata More FAIR Using Large Language Models. *ArXiv Prepr ArXiv230713085*. 2023; doi: https://doi.org/10.48550/arXiv.2307.13085.

7. Jensen LJ, Saric J, Bork P. Literature mining for the biologist: from information retrieval to biological discovery. *Nat Rev Genet*. Nature Publishing Group UK London; 2006; doi: https://doi.org/10.1038/nrg1768.

8. Harmston N, Filsell W, Stumpf MP. What the papers say: Text mining for genomics and systems biology. *Hum Genomics*. Springer; 2010; doi: https://doi.org/10.1186/1479-7364-5-1-17.

9. Blanchy G, Albrecht L, Koestel J, Garré S. Potential of natural language processing for

metadata extraction from environmental scientific publications. *Soil*. Copernicus Publications Göttingen, Germany; 2023; doi: https://doi.org/10.5194/soil-9-155-2023.

10. Salton G, Buckley C. Term weighting approaches in automatic text retrieval. Cornell University; 1987.

11. Adelman JS, Brown GD, Quesada JF. Contextual diversity, not word frequency, determines word-naming and lexical decision times. *Psychol Sci*. SAGE Publications Sage CA: Los Angeles, CA; 2006; doi: https://doi.org/10.1111/j.1467-9280.2006.01787.x.

12. Masoumi S, Amirkhani H, Sadeghian N, Shahraz S. Natural language processing (NLP) to facilitate abstract review in medical research: the application of BioBERT to exploring the 20-year use of NLP in medical research. *Syst Rev*. Springer; 2024; doi: https://doi.org/10.1186/s13643-024-02470-y.

13. Naveed H, Khan AU, Qiu S, Saqib M, Anwar S, Usman M, et al.. A comprehensive overview of large language models. *ArXiv Prepr ArXiv230706435*. 2023; doi: https://doi.org/10.48550/arXiv.2307.06435.

14. Polak MP, Morgan D. Extracting accurate materials data from research papers with conversational language models and prompt engineering. *Nat Commun*. Nature Publishing Group UK London; 2024; doi: https://doi.org/10.1038/s41467-024-45914-8.

15. Cinquin O. ChIP-GPT: a managed large language model for robust data extraction from biomedical database records. *Brief Bioinform*. Oxford Academic; 2024; doi: https://doi.org/10.1093/bib/bbad535.

16. Rodrigues JFM, Tackmann J, Malfertheiner L, Patsch D, Perez-Molphe-Montoya E, Näpflin N, et al.. The MicrobeAtlas database: Global trends and insights into earth's microbial ecosystems. *bioRxiv*. Cold Spring Harbor Laboratory; 2025; doi: https://doi.org/10.1101/2025.07.18.665519.

17. Gaio D. metadmin (Docker image); https://hub.docker.com/r/gaiotransposon/metadmin.

18. Gaio D. metadata_mining (Github repository); https://github.com/GaioTransposon/metadata_mining/releases/tag/v1.0.0.

19. Gaio D. Data for github repository https://github.com/GaioTransposon/metadata_mining. Zenodo; 2026; doi: 10.5281/ZENODO.16100606.

20. Kim L, Lavrinienko A, Sebechlebska Z, Stoltenberg S, Bokulich NA. Tier-based standards for FAIR sequence data and metadata sharing in microbiome research. *Nucleic Acids Research*. 2025; doi: https://doi.org/10.1093/nar/gkaf777.

21. Sundaram SS, Solomon B, Khatri A, Laumas A, Khatri P, Musen MA. Use of a structured knowledge base enhances metadata curation by large language models. *ArXiv Prepr ArXiv240405893*. 2024;

22. Schmidt-Lebuhn AN, Knerr N. Large Language Models can extract morphological data from taxonomic descriptions, but their stochastic nature makes automation challenging: a test on Australian Asteraceae. *PhytoKeys*. 261:1892025;

23. Gaio D. Global distribution of microbiome samples and geographic annotation consistency. Zenodo; 2025; doi: https://zenodo.org/doi/10.5281/zenodo.17436922.

24. Gaio D. Distribution of the mismatching samples color coded by distance between GPT predicted location and coordinates-extracted geo location. Zenodo; 2025; doi: https://zenodo.org/doi/10.5281/zenodo.15274466.

Figure 1

Click here to access/download;Figure;20251002_manuscript_figs_sup

Figure 1. Summarized pipeline. For a detailed pipeline see Supplementary Figure 1.

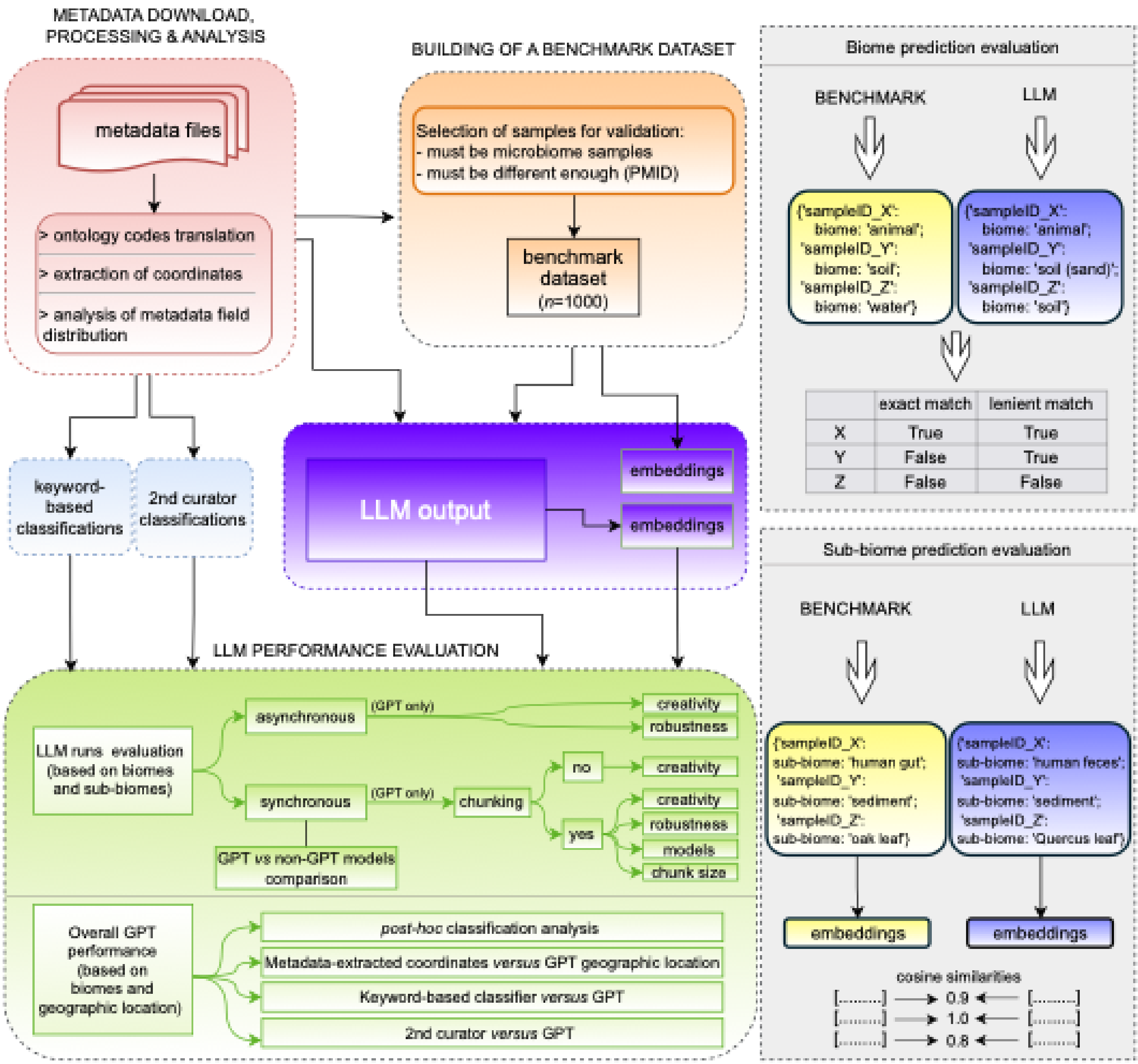

Figure 2
Click here to access/download;Figure;20251002_manuscript_figs_sup

Figure 2. Heatmaps of biome classification accuracy (an average over all GPT runs, n=105). Accuracy rates between benchmark and GPT predicted biome classifications and between benchmark and the keyword-based biome classifications are shown in the respective heatmaps. Precision and F1-score bar plots are shown on the right. Notably, the ‘unknown’ category from the previous classification is aligned with ‘other’ from GPT predictions, for direct comparison. The heatmaps are normalized by row. The benchmark dataset consists of n=1,000 samples. Overall biome accuracy: 80.6% (GPT); 62.5% (Keyword-based classifier). Cohen’s Kappa: 0.760 (GPT); 0.530 (Keyword-based classifier).

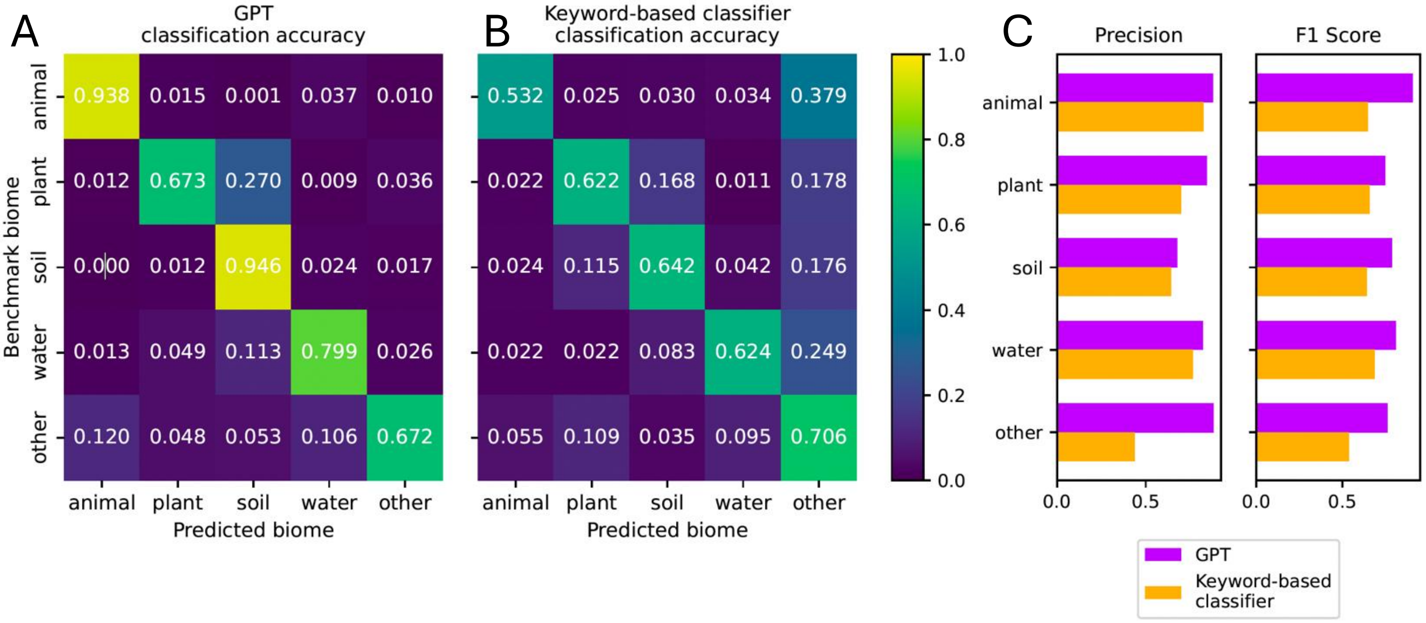

Figure 3

Click here to access/download;Figure;20251002_manuscript_figs_sup

Figure 3. Performance comparison of GPT requests with different chunking sizes. A) Accuracy percentages for biome prediction are shown, reflecting both exact matches and lenient matches between the GPT-generated output and the curator-assigned biomes. The similarity for sub-biome prediction is represented through average cosine similarity. Chunk size (2000 up to 6000) refers to the number of tokens within a single request *i.e.*, chunk. The label "chunk_no" refers to no chunking, where metadata from a single sample is sent in a single request. B) P-values (top of each cell) and adjusted p-values (bottom of each cell) of the performance comparisons are displayed. Cells shaded in green represent the statistical significance of biome accuracy comparisons, while those in blue denote the significance of sub-biome similarity comparisons. The color intensity varies according to the p-value significance. McNemar's and paired t-tests were performed for biome and sub-biome prediction comparisons, respectively. Bonferroni correction was applied on p-values.

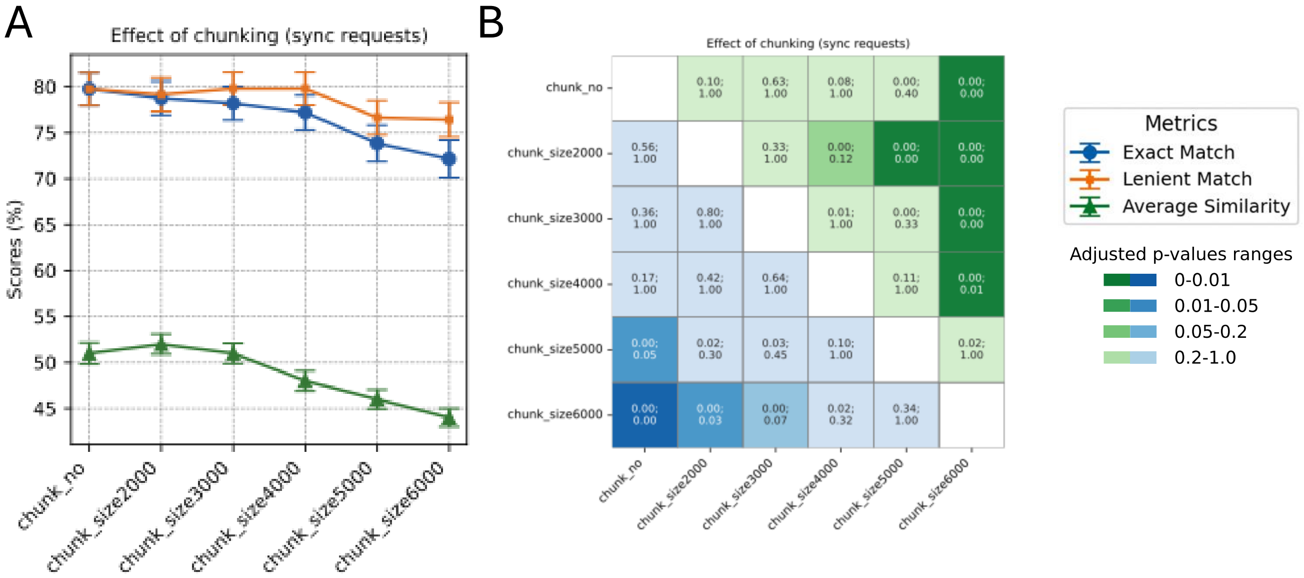

Figure 4 Click here to access/download;Figure;20251002_manuscript_figs_sup

Figure 4. Performance comparison of GPT models. A) Accuracy scores for biome prediction are shown, reflecting both exact matches and lenient matches between the GPT-generated output and the curator-assigned biomes. The similarity for sub-biome prediction is represented through average cosine similarity. B) P-values (top of each cell) and adjusted p-values (bottom of each cell) of the performance comparisons are displayed. Cells shaded in green represent the statistical significance of biome accuracy comparisons, while those in blue denote the significance of sub-biome similarity comparisons. The color intensity varies according to significance. Each run had a replicate (suffix “rep”). McNemar’s and paired t-tests were performed for biome and sub-biome prediction comparisons, respectively. Bonferroni correction was applied on p-values.

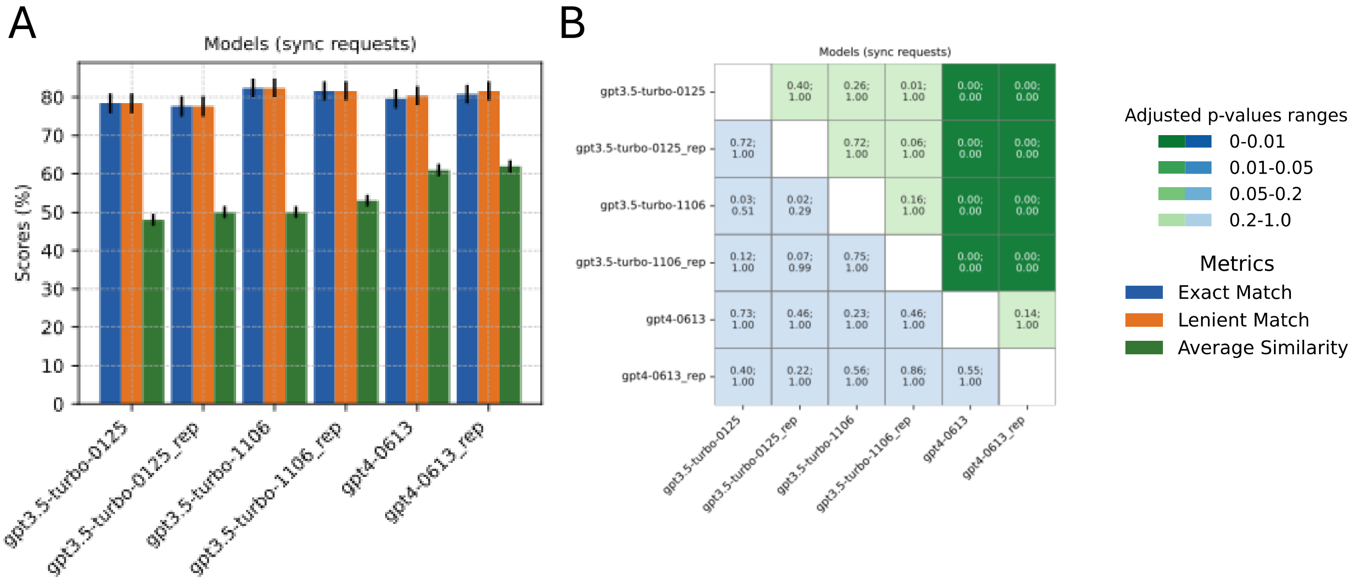

Figure 5 Click here to access/download;Figure;20251002_manuscript_figs_sup

Figure 5. Performance comparison of *GPT-3.5-turbo-1106* using either in-line or json output format. A) Accuracy scores for biome prediction are shown, reflecting both exact matches and lenient matches between the GPT-generated output and the curator-assigned biomes. The similarity for sub-biome prediction is represented through average cosine similarity. B) P-values (top of each cell) and adjusted p-values (bottom of each cell) of the performance comparisons are displayed. Cells shaded in green represent the statistical significance of biome accuracy comparisons, while those in blue denote the significance of sub-biome similarity comparisons. The color intensity varies according to the p-value significance. McNemar's and paired t-tests were performed for biome and sub-biome prediction comparisons, respectively. Bonferroni correction was applied on p-values.

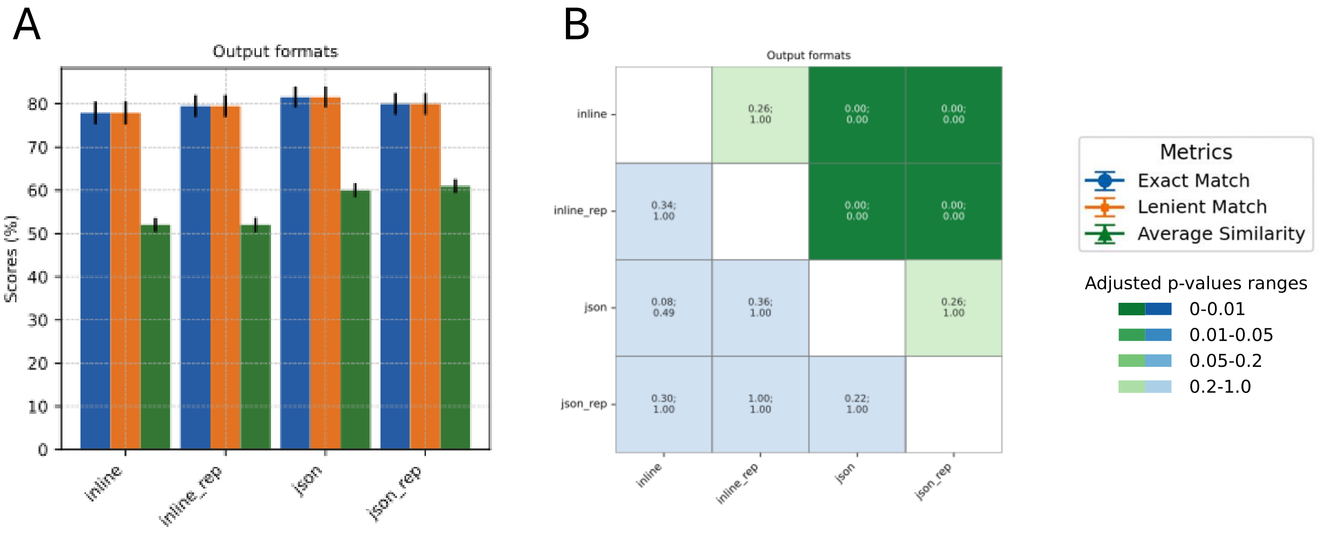

Figure 6
Click here to access/download;Figure;20251002_manuscript_figs_sup

Figure 6. Performance comparison of *GPT-3.5-turbo-1106* when tweaking frequency penalty. A) Accuracy scores for biome prediction are shown, reflecting both exact matches and lenient matches between the GPT-generated output and the curator-assigned biomes. The similarity for sub-biome prediction is represented through average cosine similarity. B) P-values (top of each cell) and adjusted p-values (bottom of each cell) of the performance comparisons are displayed. Cells shaded in green represent the statistical significance of biome accuracy comparisons, while those in blue denote the significance of sub-biome similarity comparisons. The color intensity varies according to the p-value significance. For an assessment of all other creativity parameters see Supplementary Figure 3. McNemar's and paired t-tests were performed for biome and sub-biome prediction comparisons, respectively. Bonferroni correction was applied on p-values.

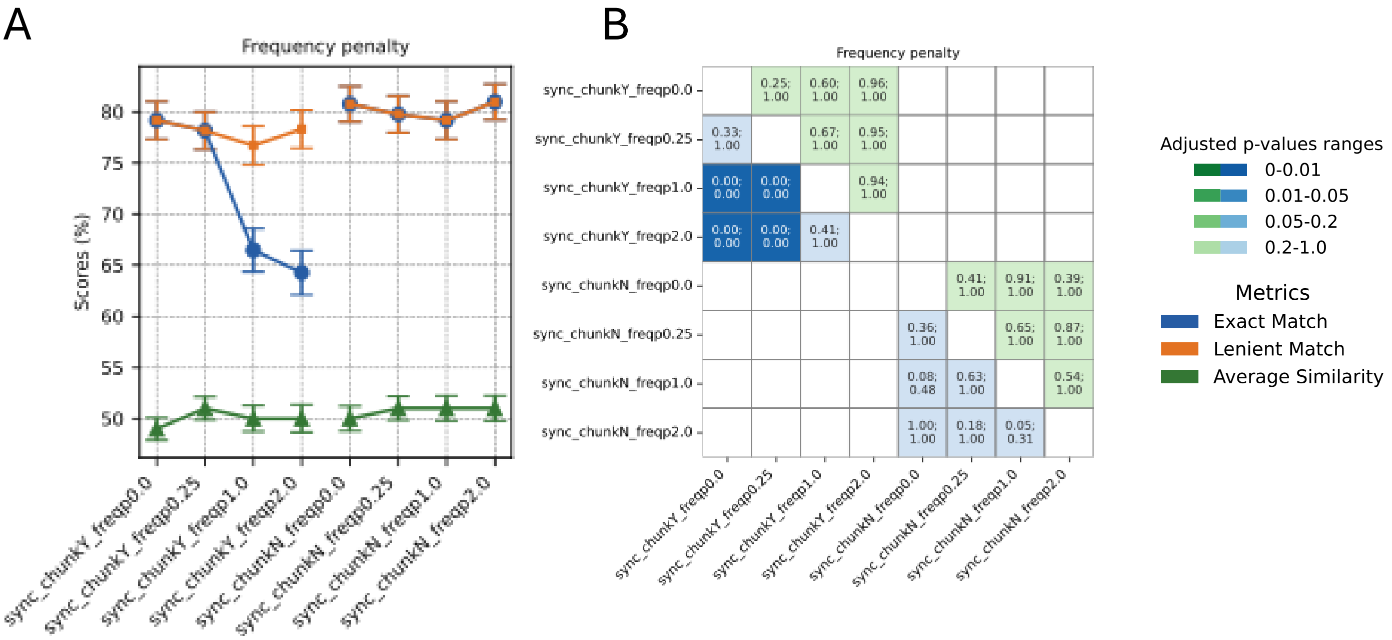

Figure 7 Click here to access/download;Figure;20251002_manuscript_figs_sup

Figure 7. Global distribution of microbiome samples and geographic annotation consistency. A heatmap (blue–green–yellow–red gradient) depicts the global density of a subset of 200,000 microbiome samples (from a pool of 990,172), with warmer colors indicating regions of higher sampling intensity. Overlaid circular clusters mark samples where GPT-inferred geographic locations did not match coordinates extracted from the metadata. Cluster color indicates the number of mismatched samples (green = few, yellow = moderate, red = many). Together, these layers highlight both the uneven geographic distribution of microbiome sampling and the spatial patterns of metadata inconsistencies. The interactive figure can be downloaded from here.

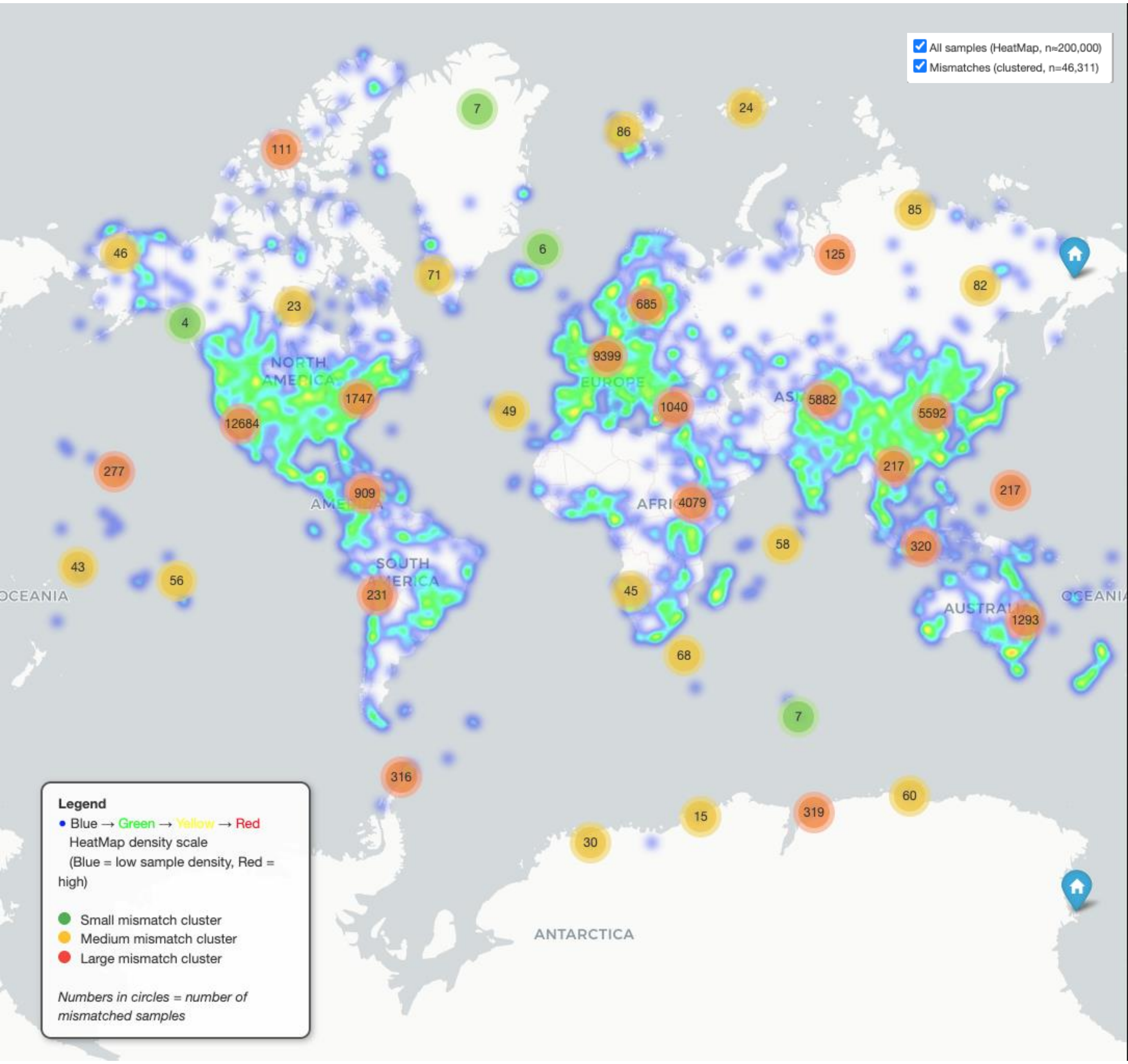

Figure 8
Click here to access/download;Figure;20251002_manuscript_figs_sup

Figure 8. Comparison of Large Language Models (LLMs) and embedding models for biome and sub-biome classification performance. **A**) Accuracy of biome predictions across models, shown as both exact and lenient matches between LLM-generated outputs and curator-assigned biome annotations. Lowercase letters (*a*–*c*) above points indicate results of *post hoc* groupings: models that share the same letter (*e.g.*, all marked "a") do not differ significantly, whereas models with different letters (*e.g.*, "a" *vs*. "b" or "c") show statistically significant differences in accuracy (McNemar's test, Bonferroni-adjusted $p < 0.05$). B) Average cosine similarity between sub-biome annotations generated by the LLMs and converted into embeddings using four different embedding models. Statistical significance for biome predictions was assessed using McNemar's test, and for sub-biome similarities using paired t-tests. Bonferroni correction was applied to adjust p-values for multiple comparisons.

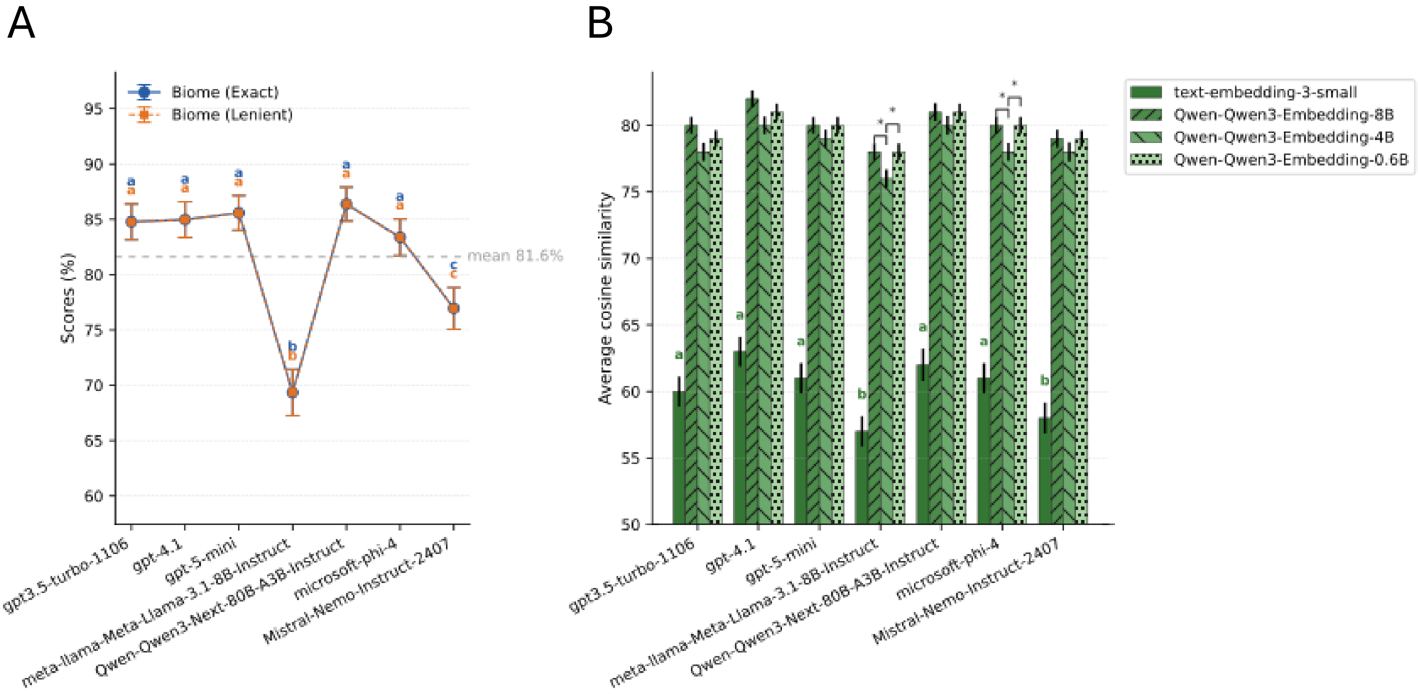

Supplementary Figure 1

Click here to access/download
Supplementary Material
20251002_manuscript_figs_supplfigs-pages-9.pdf

Supplementary Figure 2

Click here to access/download
Supplementary Material
20251002_manuscript_figs_supplfigs-pages-10.pdf

Supplementary Figure 3

Click here to access/download
Supplementary Material
20251002_manuscript_figs_supplfigs-pages-11.pdf

Supplementary Figure 4

Click here to access/download
Supplementary Material
20251002_manuscript_figs_supplfigs-pages-12.pdf

Supplementary Figure 5

Click here to access/download
Supplementary Material
20251002_manuscript_figs_supplfigs-pages-13.pdf

Supplementary Figure 6

Click here to access/download
Supplementary Material
20251002_manuscript_figs_supplfigs-pages-14.pdf

Supplementary Figure 7

Click here to access/download
Supplementary Material
20251002_manuscript_figs_supplfigs-pages-15.pdf

Supplementary Figure 8

Click here to access/download
Supplementary Material
20251002_manuscript_figs_supplfigs-pages-16.pdf

Supplementary Figure 9

Click here to access/download
Supplementary Material
20251002_manuscript_figs_supplfigs-pages-17.pdf

Dear Hongfang Zhang,

We would like to thank you and the reviewers for the time and thoughtful feedback provided on our manuscript *"Enhanced semantic classification of microbiome sample origins using Large Language Models (LLMs)"* (GIGA-D-20-00347).

We have carefully considered all comments and substantially revised the manuscript. The main updates are summarized below, while detailed, point-by-point responses follow.

The most significant revision concerns reproducibility, as raised by Reviewer 2. During re-examination of our Dockerized scripts, we identified a silent bug in the file-handling logic of validate_biomes_subbiomes.py, which caused output concatenation and ambiguous comparisons between mismatched runs. This issue has now been fully corrected. Container 4 now generates clean outputs at each run, and the Zenodo repository (new DOI created for new version - please see revised manuscript) and documentation have been reorganized to ensure full reproducibility and transparency.

The second major revision expands the manuscript's analytical scope. In response to Reviewer 1, we added results for more recent proprietary models (GPT-4.1, GPT-5-mini) and several open-weight LLMs (*e.g.*meta-llama/Meta-Llama-3.1-8B-Instruct, Qwen/Qwen3-Next-80B-A3B-Instruct, microsoft-phi-4, and Mistral-Nemo-Instruct-2407). This addition considerably strengthens the study: several open-weight models achieved comparable or superior accuracy at a fraction of the cost, showing that large-scale metadata re-annotation is now feasible without dependence on commercial APIs.

Given the importance and technical nature of this new analysis, we have added Matteo Peluso as a co-author. He is an expert in machine learning and contributed substantially to the implementation of the open-weight model experiments introduced in response to Reviewer 1's comment.

We also wish to thank Reviewer 3 whose insightful remarks helped us clarify the manuscript's broader relevance with respect to data reusability and the FAIR principles. We have strengthened the introduction and discussion to explicitly connect our work to FAIR and improved readability for both technical and non-technical audiences.

Additionally, we would like to kindly ask whether it would be possible to include or confirm the ORCID identifiers of two co-authors, Eugenio Perez-Molphe-Montoya and David Patsch, in the revised submission. We are not certain whether their ORCIDs were included in the first submission and would appreciate the opportunity to ensure that all author records are complete and accurate.

- Eugenio Perez-Molphe-Montoya: 0009-0002-9592-9455
- David Patsch: 0009-0002-9859-091X

All links have been updated in the revised manuscript:

- Docker image: https://hub.docker.com/r/gaiotransposon/metadmin (directs to the latest)
- Interactive map figures: https://zenodo.org/records/17436923 (new doi) and https://zenodo.org/records/17436804. (old doi, new version)
- Deposited data: https://doi.org/10.5281/zenodo.17437043

We believe these revisions have enhanced the manuscript's clarity, reproducibility, and long-term relevance. We are grateful for the reviewers' work, and the constructive feedback we received, which has led to a stronger and more comprehensive paper.

Kind regards

Daniela Gaio (on behalf of all co-authors)

###

Response to Reviewers:

First of all, we would like to thank all three reviewers for their valuable input in our manuscript. We believe the comments were on point, and the changes to the manuscript that followed made a significant impact on its quality and clarity.

Reviewer #1: The manuscript presents a carefully executed study using non-finetuned GPT models to classify microbiome sample metadata. It is very well written, and both the analyses and the interpretations are generally sound. I found the evaluation thorough and the presentation clear.

1. The study provides a detailed evaluation of LLM-based metadata curation, clearly advancing over keyword-based approaches. However, it is surprising that recent related studies using LLMs for metadata curation are not cited. For completeness, I suggest including references such as:
- https://doi.org/10.1093/gigascience/giaf070 (disclaimer: I am an author of the paper. You might like the table 3),
- https://doi.org/10.1093/bib/bbad535 ,
- https://doi.org/10.3897/phytokeys.261.158396 ,
- https://pmc.ncbi.nlm.nih.gov/articles/PMC12099408/.

We thank Reviewer 1 for the references! We agree that our cited literature was not fully up to date with the most recent advancements in the field (2024-2025). We have added the references above throughout the text and a few more references.

2. The scale of the processed data is impressive. However, there appears to be a discrepancy: the Zenodo repository file metadata.out contains 2,254,619 accession IDs (presumably the input), while the GPT output files (gpt_clean_output) include only around 1,000 samples (presumably the benchmark dataset), whereas the manuscript states that 3.8 million samples were processed. It would be helpful to clarify these numbers and, if applicable, explain why fewer outputs are provided. I also recommend reorganizing the Zenodo repository so that readers can download individual files rather than the entire large archive.

Indeed, we did not fully clarify these numbers:
- The manuscript does state the initial pool of 3.8M samples, but that reflects in fact the initial pool, prior to any filtering. 3.8M is the amount of samples that were originally downloaded from NCBI SRA for having relevant metadata keywords such as "metagenomic", "microb*", "bacteria", or "archea". We have now made that clearer. (lines: 157-161)
- The gpt_clean_output* files are the outputs that have been used for validation, so they indeed only contain the sample IDs from the benchmark (n=1000). We have now

added throughout that 1000 is the number of samples used for the benchmark. (lines: 24, 33, 118, 211, 393, 653)
- 2,254,619 are the actual sample IDs that have been submitted for the production run. We now explicitly report the output size of the production run (over 2M samples). (lines: 33-34, 119, 473)
- We clarify in the abstract and in the introduction the numbers to be expected from the validation of the pipeline (1000) and from the production run (over 2M). (lines: 24, 34)
- We noticed that the methods section was missing mentioning the number of samples (1000) used for validation. We have now included this. (line: 211)
- Additionally, in our previous version we did not provide the production run output (neither in the manuscript nor on Zenodo). We now mention its availability in various parts in the manuscript (abstract, introduction, data availability) and we added the files to a new Zenodo DOI. (lines: 34, 560, 889)
- Concerning the large size of the Zenodo repository: we considered splitting it into various ones, but this could be on the other side also a hassle for some users. Instead of splitting its content into various Zenodo links, we now provide a description file which describes what each file contains.

3. The data processing pipeline on GitHub is very useful. The repository currently indicates a CC0 (public domain) license. Since CC0 is typically intended for datasets rather than source code, please clarify whether this was intentional or if a software-specific license (e.g., MIT, Apache 2.0) would be more appropriate.

Very good point. We initially had CC0 because we also had data in the repository (before creating the Zenodo link). It is indeed better to have a more suited license, like MIT. The repo has been updated accordingly.

4. A different typeface appears in some paragraphs (e.g., pp. 19 and 21). Please check whether this was intentional.

Yes, this was unintentional. It has been corrected.

5. The finding that grouping 5-17 samples per request does not substantially affect accuracy is interesting. Given that GPT models often fail with counting or item listing, the observed quality decline with larger chunk sizes seems reasonable and aligns with expectations.

It is indeed expected, and that is what we see with larger chunks. We think we should have also mentioned the mean there, because although 17 samples is the maximum per chunk (in a chunksize 3000) and the median is 5, the mean is actually 6. Taking the mean into consideration, our result (“no significant difference with no-chunking”) becomes more intuitive. In the updated version of the manuscript we now report the mean. (line 481)

The pattern of biome accuracy dropping as larger chunks are used confirms the expectation of a detrimental effect when using more samples, even though significance (after *post hoc*) is reached only between 2000 and 6000 for biome and between 2000 and 5000 for sub-biome (sub-biome accuracy more is more sensitive to chunking). The model likely suffers from “context interference” once it exceeds its optimal working memory window.
It was interesting to us to observe which parts of a given chunk suffer more from this, and in some preliminary tests we investigated this. Our hypothesis was that the *last* or *middle* part samples were the ones more likely to get misannotated. What we found was that the *middle* ones were the ones to suffer most at chunksize 5000 and both (*middle* and *last* samples) to suffer the most at chunksize 6000 (see below). We did not include this analysis in the manuscript for the sake of conciseness.

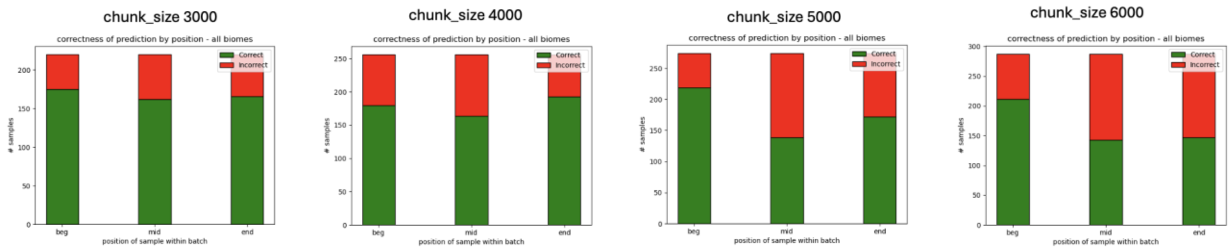

6. On p. 26, the observed variability in field usage may be linked to the BioSample package system used for submission (see: https://www.ncbi.nlm.nih.gov/biosample/docs/packages/). Some fields, such as env_biome and env_feature, were once mandatory for environmental samples but are currently optional, I suppose. Such historical changes may partly explain biases in field usage.

That is an interesting point, and yes, it could help explain our results. We have added a sentence to the Discussion to acknowledge this as a possible contributing factor. (lines 806-809)

7. The manuscript appropriately highlights the presence of ambiguous or unresolvable sample descriptions. We reached a similar conclusion in our own work with local LLMs: in many cases, even expert curators cannot determine a "correct" label, and the right answer may depend on context or application.

We agree. Possibly, the implementation of more structured, hierarchical metadata submission forms could help mitigate this issue. For instance, a decision tree–type system that guides submitters through successive levels of sample description (e.g., first selecting *“host-associated”* vs. *“free-living”*, and then specifying subcategories) would greatly improve metadata consistency. This is along the lines of Thompson et al (2017) https://www.nature.com/articles/nature24621/figures/1. At the moment, clustering a posteriori of free-text sample descriptions is a major challenge.

8. The observation that JSON output significantly improves sub-biome classification accuracy is intriguing and consistent with our internal experience with local LLMs. Since output format may also affect processing speed, it would be useful to report whether response times differed between JSON and inline formats.

That’ s an interesting point.
We have two inline-format GPT outputs that were completed in 57’’ and 1’55’’ (batch numbers: batch_67164dbbe8b081908bf02a4d2b6e2f65 and batch_67164db65fd0819097f31ac6c55e8caf, respectively).
The two json-format GPT output were completed in 59’’ and 56’’ (batch_67164da1e4ac81909991913126e9eecb and batch_67164d8ded048190badb01069142cbda, respectively).

Corresponding files (on Zenodo):

gpt_clean_output_nspb50_chunkingno_chunksize3000_modelgpt-3.5-turbo-1106_temp1.0_maxtokens4096_topp0.75_freqp0.25_presp1.5_rs22_formatjson_batch67164d8ded048190badb01069142cbda_dt202410211448.csv
gpt_clean_output_nspb50_chunkingno_chunksize3000_modelgpt-3.5-turbo-1106_temp1.0_maxtokens4096_topp0.75_freqp0.25_presp1.5_rs22_formatjson_batch67164da1e4ac81909991913126e9eecb_dt202410211448.csv
gpt_clean_output_nspb50_chunkingno_chunksize3000_modelgpt-3.5-turbo-1106_temp1.0_maxtokens4096_topp0.75_freqp0.25_presp1.5_rs22_formatinline_batch67164db65fd0819097f31ac6c55e8caf_dt202410211448.csv
gpt_clean_output_nspb50_chunkingno_chunksize3000_modelgpt-3.5-turbo-1106_temp1.0_maxtokens4096_topp0.75_freqp0.25_presp1.5_rs22_formatinline_batch67164dbbe8b081908bf02a4d2b6e2f65_dt202410211449.csv

Based on these results currently included in the manuscript (and uploaded on Zenodo), we have too few data points to draw conclusions on speed.

However, we were curious and ran 10 more async runs for inline and 10 more async runs for json (each run 2x the size of samples as the above i.e. 100 nspb → 500 samples in total). We did so by alternating the requests (1 inline, 1 json, 1 inline, 1 json, and so forth). These are the completion times we obtained:

inline: 18', 17', 20', 3' 17'', 20', 18', 18', 4' 43'', 17', 20'
json: 3' 49'', 3' 13'', 17', 3' 13'', 11', 18', 18', 17', 17', 17'

Inline stats:
Mean: 936.0 s (15.60 min)

Median: 1080.0 s (18.00 min)
SD: 373.9 s (6.23 min)
SEM: 118.2 s (1.97 min)

JSON stats:
Mean: 751.5 s (12.53 min)
Median: 1020.0 s (17.00 min)
SD: 395.7 s (6.60 min)
SEM: 125.1 s (2.09 min)

Although there seems to be a small difference, it doesn' t reach significance (Welch's T-test: t = 1.072, p = 0.2981).

We think it's likely that with a larger sample size, we would observe that json format are processed faster than inline, but with the data we currently have and the test we performed we can' t draw the conclusion. Importantly, it needs to be taken into consideration that completion times for remote LLM queries can be confounded by variable resource allocation and network conditions, making them inherently noisy.

9. One major limitation of the study is the dependence on proprietary GPT models accessible only via OpenAI's API. This constrains reproducibility and long-term availability. Indeed, the recent release of GPT-5 already renders some of the reported results outdated. While the present study remains highly valuable, it would be worthwhile to also evaluate local or open-source LLMs to ensure future reproducibility.

We completely agree with Reviewer 1. We now have included open-weight models in our analysis and manuscript, and we also added two more recent GPT models (GPT-4.1 and GPT-5-mini) to make the study more current.
To our surprise, we found that several open-weight models performed similarly to proprietary GPT models in both biome and sub-biome classification accuracy, while other models were less performant. We found that Qwen3-Next-80B-A3B-Instruct achieved comparable or even slightly higher accuracy than GPT-4.1 and GPT-5-mini, while models such as microsoft-phi-4 also demonstrated strong, consistent performance. (lines 593-617)
We also found that Qwen embedding models (also open-weight) consistently outperformed OpenAI's text-embedding-3-small. (618-637)
In our revised discussion we report a cost comparison (open-weight models were typically 10–50 times lower than proprietary GPT models) and we also clarify why there is a cost to begin with: although open-weight models are freely available, running them locally requires dedicated GPU resources, and at present it is often more economical to use third-party hosting platforms (*e.g.*, DeepInfra) than to purchase and maintain GPUs on site. (638-647)
We are particularly glad to have conducted this analysis in response to Reviewer 1's suggestion, as it delivers a strong message: ecological metadata annotation is no

longer restricted to commercial APIs. Open-weight models can be run repeatedly to build consensus across predictions, increasing confidence in the annotations. Besides the substantial cost reductions, the use of open-weight models enhances transparency, reproducibility, and long-term sustainability. Overall, this additional analysis proved well worth the effort, as it strengthened the manuscript both scientifically and conceptually by demonstrating that high-quality, reproducible metadata annotation can now be achieved with open tools.

Reviewer #2: Reproducibility report for: Enhanced semantic classification of microbiome sample origins using Large Language Models
Journal: Gigascience
ID number/DOI: GIGA-D-25-00316
Reviewer(s): Laura Caquelin, Department of Clinical Neuroscience, Karolinska Institutet, Sweden [Wrote the report and reproduced the results]

----

1. Summary of the Study

This study evaluates whether Large Language Models (LLMs) can help re-annotate sequencing records. Using GPT models, the authors tested scalability, time, cost, and performance against a benchmark of 1,000 hand-curated examples. They then applied this approach to million environmental sequencing records, producing standardized annotations.

----

2. Scope of reproducibility

According to our assessment the primary objective is: to evaluate how closely GPT's annotation performance approached that a human expert when classifying environmental sequencing samples into biomes and sub-biomes.

- Outcome: Accuracy of biome and sub-biome classification compared against a hand-curated benchmark dataset.

- Analysis method outcome: As described to validate biome classifications: "For paired comparisons of repeated sample IDs, we use the McNemar test, which is appropriate for paired binary outcomes (True/False)", while "for comparisons across different sample sets, we employ the t-test for independent samples. In both scenarios, a Bonferroni correction is applied to adjust for multiple comparisons".

For sub-biomes, comparisons across different sets were performed with independent t-tests, while "for runs involving the same sample IDs, comparisons are performed using the paired t-test". Section "Validation statistics" page 13-14.

- Main result: "The improvement in accuracy between GPT's initial classification and the human's performance with the improved prompt was statistically significant (adj p-value=0.031), but also between the human's attempt first attempt (with the initial prompt) and the human's second attempt (better prompt) (adj p-value≤0.001). No significantly different performances were detected for the sub-biome classification, neither between GPT and the human, nor between prompt versions (adjp-value=1)." Section "Human versus GPT classification accuracy" pages 18-19.

----
3. Availability of Materials

a. Data

- Data availability: Open
- Data completeness: Complete = all data necessary to reproduce main results are available
- Access Method: Repository
- Repository: https://zenodo.org/records/16100607
- Data quality: Complete but no metadata associated with the file

Yes, that is correct, we did not add the file to the Zenodo repository. Instead we only put it on the Github repo. The file is called “MicrobeAtlasProject_files_list_Zenodo.tsv”. In the new Zenodo upload (link reported in the revised manuscript), we now include this file.

b. Code

- Code availability: Open
- Programming Language(s): Python
- Repository link: https://github.com/GaioTransposon/metadata_mining/tree/main
- License: CC0
- Repository status: Public
- Documentation: Readme file clear but require one modification

Yes, that is correct. We updated the README.md file to avoid confusing users into making ~/MicrobeAtlasProject/MicrobeAtlasProject. We updated the “Requirements” section of the README.md file.

----

4. Computational environment of reproduction analysis

- Operating system for reproduction: MacOS 15.6.1
- Programming Language(s): Python
- Code implementation approach: Using shared code
- Version environment for reproduction: Python 3.13.7

----

5. Results

5.1 Original study results

- Results:

"The improvement in accuracy between GPT's initial classification and the human's performance with the improved prompt was statistically significant (adj p-value=0.031), but also between the human's attempt first attempt (with the initial prompt) and the human's second attempt (better prompt) (adj p-value≤0.001). No significantly different performances were detected for the sub-biome classification, neither between GPT and the human, nor between prompt versions (adjp-value=1)."

(The authors identified an error in the manuscript text during the review. Therefore, the following part of the manuscript needs to be updated (see email exchange with the authors below).

5.2 Steps for reproduction

-> Run the two first scripts of the container 4 in the Github: validate_biomes_subbiomes.py and overall_analysis.py

- Issue 1: The README instructions for setting up the ~/MicrobeAtlasProject directory can lead to a nested folder structure (~/MicrobeAtlasProject/MicrobeAtlasProject) if followed literally. This causes the Docker container to fail when attempting to access required files like gpt_file_label_map.tsv, since they are not found at the expected path /MicrobeAtlasProject/.

-- Resolved: The issue was resolved by manually renaming and flattening the directory structure after extraction, ensuring that the contents of MicrobeAtlasProject_Zenodo are directly placed inside ~/MicrobeAtlasProject/. However, the current instructions can mislead users, so a clarification in the README would be helpful.

Yes, that is correct. We updated the README.md file to avoid confusing users into making ~/MicrobeAtlasProject/MicrobeAtlasProject. We updated the "Requirements" section of the README.md file.

- Issue 2: During the execution of the overall_analysis.py script, multiple files with the same label were found, requiring manual selection of the file to use for the analysis.

-- Resolved: The manuscript does not specify which file should be selected to reproduce the results, leading to potential ambiguity. By default, I chose the most recent file among the options, assuming it reflects the final data version used in the manuscript. It would be helpful if the documentation or manuscript explicitly stated this to ensure exact reproducibility.

This was a very good point. We now edited overall_analysis.py to not prompt the user to choose from any "double labeled" file. This issue occurred because multiple GPT outputs shared identical parameters but different timestamps. As reviewer 2 suggested this caused unnecessary ambiguity. The solution we found was to remove the unnecessary GPT output files from the Zenodo repository (now reflected in the current Zenodo version).

-> Compare the results reproduced to the results presented in the manuscript

- Issue 3: The results obtained by running validate_biomes_subbiomes.py are two files: biome_subbiome_results.csv and biome_subbiome_stats.csv, which contain a large amount of output (1,284 and 48,197 rows respectively). The script overall_analysis.py provides overall performance metrics in the terminal output, but does not produce the adjusted p-values relevant to the scope of this review.

-- Unresolved: It was difficult to identify where to find the results presented in the manuscript, so an email was sent to the authors.

Yes, this was a main issue which has now been resolved. Those fairly large files (1,284 and 48,197 rows) were caused by the script concatenating on an existing file, as mentioned in our correspondence. Now the script validate_biomes_subbiomes.py has been updated to create at each run new biome_subbiome_results*.csv and biome_subbiome_stats*.csv files. There was additionally a problem with the script, that arose silently when editing the code for Docker (after our first submission and under request of the Editor). Without us realizing, the code: 1. silently filled out empty fields of biome_subbiome_results.csv and biome_subbiome_stats.csv even when embeddings .json files were missing; 2. did not group by test_type properly, so it did a comparison of everything against everything, generating an unnecessarily large file, very hard to read.

The removal of the 20 gpt output files caused some values to slightly drift. Here we list the changes (please note that the page and line numbers refer to the previous manuscript version):

- Line 395: “(precision: 81.7%, F1-score: 80.16).“ → “(precision: 82.0%, F1-score: 80.34).”
- Line 396: “n=125” → “n=105” (this is the current number of files)
- Line 397: “F1-score: 76.1%” → “F1-score: 76.4%”
- Line 400: “accuracy rates of 93.6% and 94.3%” → “accuracy with accuracy rates of 94.6% and 93.8%”
- Line 401: “In contrast, ‘plant’, ‘water’, and ‘other’ biome samples exhibit lower accuracy rates (67.2%, 79.3%, and 67.5%, respectively)” → “In contrast, ‘plant’, ‘water’, and ‘other’ biome samples exhibit lower accuracy rates (67.3%, 79.9%, and 67.2%, respectively)”
- Line 405: “precision (81.7%” → “precision (81.9%”
- Line 406: “accuracy: 93.6%” → “accuracy: 93.8%”
- Line 407: “precision: 87.8% vs. 8.24%” → “precision: 87.8% vs. 82.4%” (this was a typo)
- Lines 408-411: “While both methods struggle with plant samples, which they often misclassified as soil (Fig. 2A-B), GPT again produced fewer misclassifications on this subset (4.0% vs. 17.8%). In addition, GPT shows improved predictions in the reverse direction by mislabeling soil samples less frequently as plant biome, compared to the keyword-based classifier (1.3% vs. 11.5%).” → “Both methods struggle with plant samples, which they often misclassified as soil (Fig. 2A-B). Whereas GPT most often misclassified them as ‘soil’ (27.0%), the keyword-based classifier repartitioned the misclassified samples into either ‘soil’ (16.8%) or ‘other’ (17.8%). “ (here the values are updated as well as the phrasing)
- Line 413: “as indicated by Cohen’s kappa values (GPT: 0.757” → “as indicated by Cohen’s kappa values (GPT: 0.760”
- Line 420: “chi-squared” → chi-squared: 7482
- Line 421: “A large fraction of misclassified ‘animal’ samples (49.9%) are erroneously predicted as ‘water’.” → “A large fraction of misclassified ‘animal’ samples (53.0%) are erroneously predicted as ‘water’.”
- Lines 420-423: “misclassifications within ‘plant’ (82.9%) and ‘water’ samples (59.1%) are ‘soil’” → “misclassifications within ‘plant’ (83.9%) and ‘water’ samples (59.0%) are ‘soil’”
- Lines 424-425: “skewness: 1.32; kurtosis: 0.23” → “skewness: 1.34; kurtosis: 0.27”
- Lines 426-427: “Notably, 23 samples (5% of the total) fall at or above the 95th percentile, with 109 or more misclassifications” → “Notably, 21 samples (5.3% of the total) fall at or above the 95th percentile, with 90 or more misclassifications”
- Lines 428-430: “Across 117 independent classification runs using different parameter settings, the average number of misclassifications per sample is 26.0 (SD: 36.0).” → “Across 105 independent classification runs using different parameter settings, the average number of misclassifications per run is 21.7 (SD: 29.9).”
- Lines 430-431: “Half of the samples are misclassified 5 times or fewer, and three-quarters have no more than 41 misclassifications” → “Half of the samples are misclassified 4 times or fewer, and three-quarters have no more than 34 misclassifications”
- Line 432: “The maximum observed was 117” → “The maximum observed was 97”

- Line 436: “SRS4776621” → “SRS5304049” (this is another rhizosphere sample that popped in the updated results - the same count for this sample: it was misclassified as soil by GPT in all but one case, as mentioned at the end of the paragraph).
- Line 438: “was incorrectly assigned to ‘water’ by GPT in 109 out of 117 instances” → “was incorrectly assigned to ‘water’ by GPT in 89 out of 97 instances”
- Line 441: “assigned to ‘animal’ by GPT in 109 out of 112 instances” → “assigned to ‘animal’ by GPT in 90 out of 93 instances”
- Line 522: “accuracy was mitigated (freqp 0.0: 80.76%; freqp 2.0: 80.96%)” → “accuracy was mitigated (freqp 0.0: 79.16%; freqp 2.0: 78.31%)”
- Lines 537-553: we worded this part about the replicates better to clarify that the lower accuracy ranges seen across the sync requests compared to the async requests are due to the format difference and not the diff between sync and async. Sync versus async are the 6 replicates (times 2) results we clarify in the last paragraph.

As we refreshed the gpt files (removed the unnecessary ones) the geographic location analysis values also changed. At that point, given that we had many more samples available from the production run (unique ones), we updated the section “Geographic location prediction performance” to include all samples (990,172 instead of the previous 130,689). NB: these are not over 2M because it’s a merge between samples that were geographically annotated by GPT and samples from which metadata we could retrieve the coordinates, hence 990k only. We found values in this section to have shifted: 95.32% vs 95.94% matches, now versus before.
As we did not re-perform the manual-picking inside done in geo_check.py (picking manually 100 samples metadata and deciding who is right and why - gpt vs coordinates), we could use the same dictionary that was created earlier (it’s an argument of geo_check.py). The only thing that changed is the sample size of the analysis, and therefore also the map figures (now Figure 7 and Suppl. Figure 8).

Message sent by the authors

Dear reviewer,

By running validate_biomes_subbiomes.py as described (using gpt_file_label_map.tsv as --map_file), the output will be two .csv files named biome_subbiome_results.csv and biome_subbiome_stats.csv.

The latter file will contain the stats (hence the adjusted p-values). Were you able to reproduce such files?

We did notice there are a few mistakes.

Mistake 1.

In the manuscript it says:

" A trained molecular biologist, with no prior exposure to the project, was given the same prompt instructions as GPT and was asked to classify sample biomes and sub-biomes. While against the benchmark dataset, GPT achieved an accuracy of 79.76% (n=499; SD=40.0), the human annotator reached 78.0% (n=250; SD=33.0). "

The second standard deviation should be replaced with SD=42.0.

We re-confirm this change.

Mistake 2.

In the manuscript it says:

" The improvement in accuracy between GPT's initial classification and the human's performance with the improved prompt was statistically significant (adj p-value=0.031), but also between the human's attempt first attempt (with the initial prompt) and the human's second attempt (better prompt) (adj p-value≤0.001). "

The first adjusted p-value should not be 0.031 but 0.134 hence not significant so this sentence should be adjusted to:

" There was an improvement in accuracy between the human's first attempt (with the initial prompt) and the human's second attempt (better prompt) (adj p-value≤0.001). "

We re-confirm this change.

Mistake 3.

We built on biome_subbiome_results.csv and biome_subbiome_stats.csv further than necessary so the two files on Zenodo should be "cut" earlier to avoid confusion. This was a problem of the script validate_biomes_subbiomes.py which concatenates on existing files (e.g.: biome_subbiome_results.csv and biome_subbiome_stats.csv) instead of creating new ones. We should probably proceed by replacing these two files with the files without repetitions.

We thank you for your work and please do let us know if everything works out now.

Thank you and kind regards,

We re-confirm this change. Yes, the new Zenodo link has the correct files. The script (as mentioned above) now does not concatenate on existing files anymore, but produces fresh ones when re-run.

####

The authors confirm that the results presented in the manuscript can be found in the biome_subbiome_stats.csv file. This file contains 48197 rows. According to the authors, the file includes data from both existing files and new data. As a result, it is difficult to determine which data have been reproduced. Even when using a Ctrl+F search for the reported p-value (e.g, pvalue = 0.134) in the Excel file, this value appears in several rows labeled under different configurations such as (label1/label2):
--- chunk_size3000/sync_chunkN_presp0.0;
--- chunk_size3000/sync_chunkN_temp1.5;
--- chunk_size5000/gpt4-0613;
--- machine/ sync_chunkY_topp0.0, etc…

Yes, as mentioned above we solved this problem by 1. Replacing the correct biome_subbiome_results*.csv and biome_subbiome_stats*.csv files; 2. De-bugging validate_biomes_subbiomes.py. When we edited our scripts to work with Docker, some issues had arisen that we were unaware of. One was the script silently filling out empty fields of biome_subbiome_results.csv and biome_subbiome_stats.csv even when embeddings .json files were missing. Another issue was not properly grouping by test_type properly, so the output was very hard to read. The strange label pairing Reviewer 2 noticed, no longer happens now. Only files falling under the same column "test_type" (in gpt_file_label_map.tsv file), are compared to each other. It wouldn't make sense to compare for example "chunk_size3000" with "sync_chunkN_presp0.0". This is also why the biome_subbiome_stats.csv file was so large. We thank Reviewer 2 for spotting this important problem.

5.3 Statistical comparison Original vs Reproduced results

- Results: The biome_subbiome_stats.csv file was reproduced, but it is difficult to distinguish between the newly reproduced data and the existing data already present in the file. Additionally, the data presented in the manuscript are also hard to identify due to the size of the file. No comparison was performed.

Biome_subbiome_stats*.csv (the * now stands as we have three files each, after the addition of the new analysis with the open-weight models) now only produces the correct comparisons, and there is no concatenation to the existing file. With the file now

is being concise, we hope we have overcome the problem of poor readability. We stay open to further improvements where found necessary.

- Comments: -
- Errors detected: Authors identified an error in the manuscript text during the review with the first adjusted p-value that is not 0.031 but 0.134.

We re-confirm this change.

- Statistical Consistency: No comparison was performed.
----

6. Conclusion

- Summary of the computational reproducibility review

The main scripts to reproduced the results were successfully executed and the output files were generated. However, due to the size of the output files and the lack of precise references in the manuscript, it was difficult to identify which parts of the output correspond to the results presented in the paper. Moreover, authors mentionned that the script adds data to existing output files rather than generating new ones, making it hard to distinguish between old and new data. This led to confusion when trying to compare the reproduced results with those in the manuscript. Then a comparison of statistical values was not possible.

- Recommendations for authors

To improve the reproducibility of the manuscript, we recommend the authors to:

-- Clarify instructions in the README about the MicrobeAtlasProject folder.

Done.

-- Ensure scripts generate new outputs or clarify which data is new vs. existing in the files with for example a column indicating the origin (e.g., "new" or "existing").

Done. It now generates a new output at each run. No more concatenation.

-- Link results in the manuscript to specific rows/sections in the output files to easily locate the exact data used. Another solution could be to consider including a smaller, or a filtered version of the output files with only the rows used for key reults, figures or tables, to make checking results easier and avoid error.

This is a good suggestion by Reviewer 2, but we were wondering whether the addition in the manuscript of “see line xx of file yy” every time result values or stats are reported, would not make it much heavier to read. We believe now we have significantly reduced the size of the two files so the values are easier to read. Also correcting the issue of random labels being compared to random labels, has solved a major problem. As a result, values are easier to find. The results file is now 154 lines long (instead of 1,284) and the stats file now has 1,172 lines (instead of 48,197). It is worth noting that the new files also include the new output files generated for the comparison of OpenAI with open-weight models (new results - under request of Reviewer 1).

-- Metadata: For the data used or generated by the scripts, it would be helpful to include accompanying metadata files that explain:
--- The definition of each variable name.
--- The origin of each dataset (raw, processed, etc).
--- Any preprocessing steps applied before analysis.

Yes, this file is “MicrobeAtlasProject_files_list_Zenodo.tsv”, which we previously uploaded to our Github repo, but not on the Zenodo repository. It showed on the front page of the Zenodo repository, but it’s not downloadable. In the new Zenodo version we now include the file. Besides making the file downloadable, we expanded the descriptions for each file, as Reviewer 2 suggested.

Reviewer #3: The ability to reuse scientific data for secondary analysis is an extremely important topic. Since the promotion of the FAIR Guiding Principles a decade ago, the central importance of standards-adherent metadata has received considerable attention. Although this paper surprisingly doesn't mention the FAIR principles, the work is important in understanding what it takes to make datasets FAIR and "AI ready."

We thank Reviewer 3 for this valuable comment and for emphasizing the connection between our work and the FAIR principles. We agree that our study directly contributes to advancing dataset FAIRness and AI readiness through large-scale metadata standardization using LLMs. In response, we have revised the manuscript to explicitly acknowledge and discuss this connection with FAIR. We did so in the Abstract (line 18), Intro (lines 66,75), Results (line 386) and Discussion (lines 722, 878).

A core problem with the paper is that it is unsure who its audience is. The paper is motivated by the needs to scientists to search for and reuse online datasets for secondary analysis, but much of the paper concerns highly technical issues that are related to fine tuning LLM performance. It is laudable that the manuscript annotates its discussion of the authors' methods with pointers to actual Python scripts that would

allow third parties to replicate the authors' work. The detailed presentation, however, may make it hard for many readers to understand the computational strategy that all the scripts are implementing. The organization of the paper weaves from discussions of the ability of LLMs to extract scientific standards from "legacy" experimental metadata to details of how to enhance computational efficiency to make the use of LLMs more cost-effective. The title and abstract of the paper suggest that the authors are aiming for a more scientific audience, but much of the manuscript focuses on arcane implementation details that will be less important to such readers.

We thank Reviewer 3 for the thoughtful and constructive comment, and we appreciate the recognition of our efforts to make the pipeline fully transparent and reproducible. Our goal with this manuscript is indeed to reach several audiences:

(1) computational researchers or bioinformaticians who wish to reproduce or extend the pipeline, and

(2) scientists from molecular biology, microbiology, or ecology who regularly encounter poorly structured metadata and wish to understand how far LLMs have progressed toward assisting human curation, and

(3) scientists from molecular biology, microbiology, or ecology who operate at the intersection of experimental and computational biology.

We acknowledge that bridging these communities is ambitious, but it reflects the evolving nature of biological data science, where researchers increasingly operate at the intersection of experimental and computational fields. At present, there is no broadly adopted solution to address the widespread problem of non-standardized or effectively unusable metadata in public repositories. Our work aims to fill this gap by providing both conceptual guidance and a reproducible, step-by-step framework for improving metadata usability through LLM-based re-annotation.

At the same time, we acknowledge that the manuscript provides full transparency at the cost of lots of detail (for example explaining what each script does).Users that do not want to extend or modify the scripts will perhaps not need that level of verbosity and granularity - for them, a concise README file in the GitHub repo walks step-by-step through reproducing the pipeline.

To make the paper easier to follow for non-technical readers, we have now added an overview paragraph at the start of the Results section summarizing the logical flow of analyses and highlighting how each component connects to the study’s central question (lines 370-390). This addition helps maintain orientation through the more detailed methodological subsections that follow.

Missing from the paper is a detailed discussion of what the metadata in SRA are really like. The reader never sees complete examples of the metadata that are processed in

the authors' work, and thus it is hard to have intuition about the problem that the authors are trying to solve. In particular, the paper doesn't present information about the range of attributes in user-defined metadata fields in SRA. The paper would benefit from a discussion of the structure of scientific metadata in general, and of how the authors' work fits into the larger effort in the research community to make datasets FAIR. (Full disclosure: My own laboratory is involved in such activity. See https://arxiv.org/abs/2504.05307v2)

We thank Reviewer 3 for this valuable comment and for sharing their reference, which we have cited in the revised manuscript. We agree that showing concrete examples of metadata structure helps readers better appreciate the challenges addressed by our work. To that end, we have expanded the *Introduction* to include a discussion of typical metadata inconsistencies, now citing Ikeda *et al.* (2025), who document malformed BioSample entries, as well as our own representative metadata examples (added as *Supplementary Figure 1*). These additions provide direct insight into the heterogeneity and irregularities of BioSample metadata, highlighting why automated interpretation is required.

Regarding the “range of attributes in user-defined metadata fields in SRA,” this topic is addressed in the *Results* section (*Informative metadata fields*), which details the diversity of fields used to describe sample origin, and is further supported by *Supplementary Figure 9*. Together, these additions provide both a qualitative and quantitative view of the metadata landscape.

Finally, we note that while a deeper exploration of the metadata problem itself would indeed be valuable, we had to balance scope with readability, given that the primary focus of the manuscript is the evaluation of GPT and open-weight LLM performance for metadata re-annotation. The current version now clarifies the metadata challenges motivating this task while keeping the main emphasis on methodological evaluation and comparative performance.

The abstract of the paper states that the authors "test to what extent LLMs can be used to cost-effectively automate the re-annotation of sequencing records." Alas, the paper really examines re-annotation of only the fields for "biome" and "location." A weakness of the paper is that the reader doesn't learn what other fields may be relevant in these metadata records, and why the authors chose to focus on the particular fields that they studied. Overall, much more attention should be placed on discussion of the limitations of the work and how well the results might scale to more general problems in standardization of scientific metadata.

We agree that our analysis focuses on a subset of metadata fields and that this limitation should be explicitly acknowledged. In the revised version, we have added a paragraph to the Discussion describing this scope, clarifying that our validation was

restricted to fields related to sample origin, namely *biome*, *sub-biome*, and *geographic location*. These attributes were chosen because they are central to the MicrobeAtlas project, which serves as the empirical foundation for this study, and because they apply broadly to all sequencing samples regardless of context. They are also among the most inconsistently reported fields in public repositories, making them particularly relevant for testing LLM-based re-annotation.

Other metadata fields (*e.g.*, environmental parameters such as pH, altitude, or ocean depth; or methodological fields such as sequencing platform and library type) are indeed important for downstream analyses but were beyond the scope of this work. Many of these are numeric or at least more highly structured and can be parsed more reliably using conventional NLP or rule-based methods, whereas the biome and sub-biome fields require nuanced semantic interpretation—an area where LLMs can add the most value.

The newly added section in the Discussion explicitly acknowledges this as a limitation of the current study and frames it as a first step toward more comprehensive LLM-driven metadata standardization. We emphasize that the framework presented here can, in principle, be extended to additional metadata fields in future work.

Minor comments:

The term "biome" is never well defined.

We acknowledge a proper biome definition was missing earlier on. In the revised version we added the definition to the introduction (lines 105-114), methods (lines 174-194); and results section (lines 388-390).

Frequently, parenthetical remarks begin with "e.g." and end with "etc." This style is redundant; you need only one of these abbreviations in each instance.

Good point. We corrected this.

Page 6, para 1: "last three digits" or "last three characters"?

We relied on the “last three digits” because all sample IDs terminate with digits, whether they are SRS, ERS or DRS. For example: SRS1713087, ERS3774001, DRS005001. Using these digits to define directory names provided a simple and deterministic way to distribute millions of metadata files across subdirectories. This structure was essential for filesystem performance and scalability: storing millions of files in a single directory can dramatically slow down file access, indexing, and I/O

operations. By splitting the files into subdirectories named after the last three digits, we ensured that each folder contained a manageable number of files, enabling faster read/write operations and easier parallel processing. This approach benefits both our own workflow as well as any user intending to reproduce the pipeline.

What is the motivation for consolidating reference ontologies into a single dictionary?

This was primarily a practical and logistic choice. By consolidating them into one, users who want to reproduce the pipeline can for example directly use our pre-made dictionary (downloadable from the zenodo link in the revised manuscript), or even compare the checksum of our dictionary with theirs to know if it differs (simpler and more transparent with one unified file than with many smaller ones). Also, a single consolidated dictionary ensures consistent ontology mapping across the entire pipeline. It eliminates potential discrepancies that could arise if individual ontology files were updated independently or parsed in different orders. In this way, lookup and caching during annotation are faster, since the script can load one unified key–value structure into memory rather than repeatedly querying multiple smaller files.

Page 13, para 2: The notion of "lenient matches" requires much more discussion. If the goal is to make the legacy metadata standards-adherent, then a "lenient" match would not seem to be valid. The operative question is, "What metadata terms will users invoke to search for datasets?", and presumably users will be searching for standard terms only.

We observed that, despite strict prompt instructions to output only one of the five allowed biome labels (“animal”, “plant”, “water”, “soil”, “other”), LLMs occasionally appended short clarifying text such as “animal (human)” or “water (marine)”. These are still fully valid biome classifications—albeit somewhat more specific—but are not exact string matches to the benchmark. To quantify performance in these cases, we defined a *lenient match* as an output where one of the five biome labels appears as a complete word (e.g., “animal (human)” counts as a lenient match to “animal”). This does *not* include partial or fuzzy matches (e.g., “plan” ≠ “plant”). This criterion was only applied when assessing biome classification accuracy and only to highlight the model’s tendency to add detail when certain creativity parameters (*i.e.:* frequency penalty >0.25) were used during chunked requests (Figure 6).These are the corresponding files:

gpt_clean_output_nspb100_chunkingyes_chunksize2000_modelgpt-3.5-turbo-1106_temp1.0_maxtokens4096_topp0.75_freqp0.25_presp1.5_rs22_API118_normal_dt202406051326.txt
gpt_clean_output_nspb100_chunkingyes_chunksize2000_modelgpt-3.5-turbo-1106_temp1.0_maxtokens4096_topp0.75_freqp0.0_presp1.5_rs22_API120_normal_dt202406051442.txt

gpt_clean_output_nspb100_chunkingyes_chunksize2000_modelgpt-3.5-turbo-1106_temp1.0_maxtokens4096_topp0.75_freqp1.0_presp1.5_rs22_API132_normal_dt202406051450.txt
gpt_clean_output_nspb100_chunkingyes_chunksize2000_modelgpt-3.5-turbo-1106_temp1.0_maxtokens4096_topp0.75_freqp2.0_presp1.5_rs22_API153_normal_dt202406051500.txt

In the production run (no chunking used), where we processed over 2M samples, we never obtained extra info to the biome description requested. Please see below:

```
$ awk -F'\t' '{count[$2]++} END {for (b in count) print b, count[b]}' GPT_biomes.txt
plant 185706
other 85324
animal 1241645
air 10
food 616
water 305883
coral 1
soil 237225
```

The GPT_biomes.txt file, as well as other parsed GPT output files are now downloadable on the new Zenodo link (in the revised manuscript).

In conclusion, the lenient match metric serves purely as an analytic tool to understand how parameter changes affect model behaviour—not as a relaxation of standards for metadata annotation. In practical terms, lenient matching ensures that variants like “animal (human)” are still grouped under the canonical “animal” biome for evaluation, which reflects how such records would ultimately be indexed and retrieved by users searching for standard biome terms.

We have now clarified in the Methods (lines 302-309) what we mean by ‘lenient match,’ emphasizing that it refers only to appended clarifications (e.g., ‘animal (human)’) and is used solely for benchmarking, not for metadata standardization.

Page 17, para 3: It's unclear what is meant by "most samples have fewer misclassifications." Fewer than what?

That is a good point. It needs clearer wording as it’s comparative. We changed it from:

< The distribution of misclassifications per sample is right-skewed (skewness: 1.34; kurtosis: 0.27), indicating that while most samples have fewer misclassifications, a small subset exhibit disproportionally high values (Supplementary Figure 4). >

to:

< The distribution of misclassifications per sample is right-skewed (skewness: 1.34; kurtosis: 0.27), indicating that most samples have relatively few misclassifications compared to a small subset with disproportionately high values (Supplementary Figure 4). >

Page 24, para 2: It's not clear what you mean when you say, "In half of the cases GPT correctly predicted the location, while the lat/lon coordinates parsed from the metadata were incorrect." Are you saying that GPT gave correct results when the lat/lon data were incorrect?

In short, yes. GPT extracts the geo location in text form (*e.g.*: if the metadata reports “Orosei, Italy” or just “Orosei”, it will return “Orosei, Italy” - because the prompt instructs the LLM to “*infer geographical location where the sample was collected, including the country (NOT the coordinates)”*).
“In half of the cases” refers to half of the pool of 100 misclassified samples. These 100 misclassified samples were picked randomly (must have a discrepancy of more than 1000 km between GPT predicted geo location and the metadata derived coordinates).

Half of these (n=50) GPT guessed the location in text form correctly, while the metadata extracted coordinates were incorrect. Then we go ahead explaining why the coordinates were erroneous. This is still based on the 100 misclassified samples we manually observed to get an idea of what the underlying causes of these discrepancies might be.

It is worth noting that, in our broader dataset, a surprisingly large fraction of entries contain evidently incorrect latitude/longitude values—such as swapped coordinates, truncated decimals, misplaced signs, or mismatched hemispheres. These inconsistencies often arise from manual entry errors or from metadata formatting changes during submission to public repositories. In this context, the ability of LLMs to cross-validate location information based on textual cues offers an additional layer of quality control, flagging cases where numeric coordinates may not correspond to the declared sampling site.

Figure 1 is very busy and the tiny font is hard to read.

We updated figure 1 and increased the font.
