# Supplementary material for: Enhanced semantic classification of microbiome sample origins using large language models (LLMs)
Source: Gigascience. 2026 Feb 12;15:giag015. doi: 10.1093/gigascience/giag015 (PMC13042274; doi:10.1093/gigascience/giag015)
Supplement: giag015_Authors_Response_To_Reviewer_Comments_original_submission [file giag015_authors_response_to_reviewer_comments_original_submission.pdf]

Author's Response To Reviewer Comments

Dear Hongfang Zhang,
We would like to thank you and the reviewers for the time and thoughtful feedback provided on our manuscript "Enhanced se
microbiome sample origins using Large Language Models (LLMs)" (GIGA-D-20-00347).
We have carefully considered all comments and substantially revised the manuscript. The main updates are summarized belo
by-point responses follow.
The most significant revision concerns reproducibility, as raised by Reviewer 2. During re-examination of our Dockerized scrip
bug in the file-handling logic of validate_biomes_subbiomes.py, which caused output concatenation and ambiguous comparis
mismatched runs. This issue has now been fully corrected. Container 4 now generates clean outputs at each run, and the Zer
DOI created for new version - please see revised manuscript) and documentation have been reorganized to ensure full reproc
transparency.
The second major revision expands the manuscript's analytical scope. In response to Reviewer 1, we added results for more
models (GPT-4.1, GPT-5-mini) and several open-weight LLMs (e.g.meta-llama/Meta-Llama-3.1-8B-Instruct, Qwen/Qwen3-Ne
microsoft-phi-4, and Mistral-Nemo-Instruct-2407). This addition considerably strengthens the study: several open-weight mo
comparable or superior accuracy at a fraction of the cost, showing that large-scale metadata re-annotation is now feasible wi
commercial APIs.
Given the importance and technical nature of this new analysis, we have added Matteo Peluso as a co-author. He is an expert
and contributed substantially to the implementation of the open-weight model experiments introduced in response to Reviewe
We also wish to thank Reviewer 3 whose insightful remarks helped us clarify the manuscript's broader relevance with respect
the FAIR principles. We have strengthened the introduction and discussion to explicitly connect our work to FAIR and improve
technical and non-technical audiences.
Additionally, we would like to kindly ask whether it would be possible to include or confirm the ORCID identifiers of two co-au
Molphe-Montoya and David Patsch, in the revised submission. We are not certain whether their ORCIDs were included in the
would appreciate the opportunity to ensure that all author records are complete and accurate.

- Eugenio Perez-Molphe-Montoya: 0009-0002-9592-9455
- David Patsch: 0009-0002-9859-091X

All links have been updated in the revised manuscript:
- Docker image: https://hub.docker.com/r/gaiotransposon/metadmin (directs to the latest)
- Interactive map figures: https://zenodo.org/records/17436923 (new doi) and https://zenodo.org/records/17436804. (old d
- Deposited data: https://doi.org/10.5281/zenodo.17437043

We believe these revisions have enhanced the manuscript's clarity, reproducibility, and long-term relevance. We are grateful
and the constructive feedback we received, which has led to a stronger and more comprehensive paper.
Kind regards
Daniela Gaio (on behalf of all co-authors)

###

Response to Reviewers:

First of all, we would like to thank all three reviewers for their valuable input in our manuscript. We believe the comments we[illegible] changes to the manuscript that followed made a significant impact on its quality and clarity.

Reviewer #1: The manuscript presents a carefully executed study using non-finetuned GPT models to classify microbiome sa[illegible] very well written, and both the analyses and the interpretations are generally sound. I found the evaluation thorough and the[illegible]

1. The study provides a detailed evaluation of LLM-based metadata curation, clearly advancing over keyword-based approach[illegible] surprising that recent related studies using LLMs for metadata curation are not cited. For completeness, I suggest including r[illegible]
- https://doi.org/10.1093/gigascience/giaf070 (disclaimer: I am an author of the paper. You might like the table 3),
- https://doi.org/10.1093/bib/bbad535 ,
- https://doi.org/10.3897/phytokeys.261.158396 ,
- https://pmc.ncbi.nlm.nih.gov/articles/PMC12099408/.

We thank Reviewer 1 for the references! We agree that our cited literature was not fully up to date with the most recent adva[illegible] (2024-2025). We have added the references above throughout the text and a few more references.

2. The scale of the processed data is impressive. However, there appears to be a discrepancy: the Zenodo repository file met[illegible] 2,254,619 accession IDs (presumably the input), while the GPT output files (gpt_clean_output) include only around 1,000 sa[illegible] benchmark dataset), whereas the manuscript states that 3.8 million samples were processed. It would be helpful to clarify th[illegible] applicable, explain why fewer outputs are provided. I also recommend reorganizing the Zenodo repository so that readers ca[illegible] files rather than the entire large archive.

Indeed, we did not fully clarify these numbers:
- The manuscript does state the initial pool of 3.8M samples, but that reflects in fact the initial pool, prior to any filtering. 3.8[illegible] samples that were originally downloaded from NCBI SRA for having relevant metadata keywords such as “metagenomic”, “m[illegible] “archea”. We have now made that clearer. (lines: 157-161)
- The gpt_clean_output* files are the outputs that have been used for validation, so they indeed only contain the sample IDs [illegible] (n=1000). We have now added throughout that 1000 is the number of samples used for the benchmark. (lines: 24, 33, 118, [illegible]
- 2,254,619 are the actual sample IDs that have been submitted for the production run. We now explicitly report the output s[illegible] run (over 2M samples). (lines: 33-34, 119, 473)
- We clarify in the abstract and in the introduction the numbers to be expected from the validation of the pipeline (1000) and[illegible] run (over 2M). (lines: 24, 34)
- We noticed that the methods section was missing mentioning the number of samples (1000) used for validation. We have n[illegible] 211)
- Additionally, in our previous version we did not provide the production run output (neither in the manuscript nor on Zenodo[illegible] availability in various parts in the manuscript (abstract, introduction, data availability) and we added the files to a new Zenod[illegible] 889)
- Concerning the large size of the Zenodo repository: we considered splitting it into various ones, but this could be on the oth[illegible] some users. Instead of splitting its content into various Zenodo links, we now provide a description file which describes what [illegible]

3. The data processing pipeline on GitHub is very useful. The repository currently indicates a CC0 (public domain) license. Si[illegible] intended for datasets rather than source code, please clarify whether this was intentional or if a software-specific license (e.g[illegible] would be more appropriate.

Very good point. We initially had CC0 because we also had data in the repository (before creating the Zenodo link). It is indee
more suited license, like MIT. The repo has been updated accordingly.

4. A different typeface appears in some paragraphs (e.g., pp. 19 and 21). Please check whether this was intentional.

Yes, this was unintentional. It has been corrected.

5. The finding that grouping 5-17 samples per request does not substantially affect accuracy is interesting. Given that GPT m
counting or item listing, the observed quality decline with larger chunk sizes seems reasonable and aligns with expectations.

It is indeed expected, and that is what we see with larger chunks. We think we should have also mentioned the mean there,
samples is the maximum per chunk (in a chunksize 3000) and the median is 5, the mean is actually 6. Taking the mean into
("no significant difference with no-chunking") becomes more intuitive. In the updated version of the manuscript we now repo
The pattern of biome accuracy dropping as larger chunks are used confirms the expectation of a detrimental effect when usin
though significance (after post hoc) is reached only between 2000 and 6000 for biome and between 2000 and 5000 for sub-b
accuracy more is more sensitive to chunking). The model likely suffers from "context interference" once it exceeds its optima
window.
It was interesting to us to observe which parts of a given chunk suffer more from this, and in some preliminary tests we inve
hypothesis was that the last or middle part samples were the ones more likely to get misannotated. What we found was that
the ones to suffer most at chunksize 5000 and both (middle and last samples) to suffer the most at chunksize 6000 (see belo
this analysis in the manuscript for the sake of conciseness.

6. On p. 26, the observed variability in field usage may be linked to the BioSample package system used for submission (see
https://www.ncbi.nlm.nih.gov/biosample/docs/packages/). Some fields, such as env_biome and env_feature, were once man
environmental samples but are currently optional, I suppose. Such historical changes may partly explain biases in field usage

That is an interesting point, and yes, it could help explain our results. We have added a sentence to the Discussion to acknow
contributing factor. (lines 806-809)

7. The manuscript appropriately highlights the presence of ambiguous or unresolvable sample descriptions. We reached a sim
own work with local LLMs: in many cases, even expert curators cannot determine a "correct" label, and the right answer may
application.

We agree. Possibly, the implementation of more structured, hierarchical metadata submission forms could help mitigate this i
decision tree–type system that guides submitters through successive levels of sample description (e.g., first selecting "host-a
living", and then specifying subcategories) would greatly improve metadata consistency. This is along the lines of Thompson
https://www.nature.com/articles/nature24621/figures/1. At the moment, clustering a posteriori of free-text sample descriptio
challenge.

8. The observation that JSON output significantly improves sub-biome classification accuracy is intriguing and consistent with
with local LLMs. Since output format may also affect processing speed, it would be useful to report whether response times d
and inline formats.

That' s an interesting point.
We have two inline-format GPT outputs that were completed in 57'' and 1'55'' (batch numbers: batch_67164dbbe8b081908b
batch_67164db65fd0819097f31ac6c55e8caf, respectively).
The two json-format GPT output were completed in 59'' and 56'' (batch_67164da1e4ac81909991913126e9eecb and
batch_67164d8ded048190badb01069142cbda, respectively).

Corresponding files (on Zenodo):

gpt_clean_output_nspb50_chunkingno_chunksize3000_modelgpt-3.5-turbo-
1106_temp1.0_maxtokens4096_topp0.75_freqp0.25_presp1.5_rs22_formatjson_batch67164d8ded048190badb01069142cbd
gpt_clean_output_nspb50_chunkingno_chunksize3000_modelgpt-3.5-turbo-
1106_temp1.0_maxtokens4096_topp0.75_freqp0.25_presp1.5_rs22_formatjson_batch67164da1e4ac81909991913126e9eec

gpt_clean_output_nspb50_chunkingno_chunksize3000_modelgpt-3.5-turbo-1106_temp1.0_maxtokens4096_topp0.75_freqp0.25_presp1.5_rs22_formatinline_batch67164db65fd0819097f31ac6c55e8ca
gpt_clean_output_nspb50_chunkingno_chunksize3000_modelgpt-3.5-turbo-1106_temp1.0_maxtokens4096_topp0.75_freqp0.25_presp1.5_rs22_formatinline_batch67164dbbe8b081908bf02a4d2b6e2f

Based on these results currently included in the manuscript (and uploaded on Zenodo), we have too few data points to draw

However, we were curious and ran 10 more async runs for inline and 10 more async runs for json (each run 2x the size of sa 100 nspb → 500 samples in total). We did so by alternating the requests (1 inline, 1 json, 1 inline, 1 json, and so forth). Thes times we obtained:

inline: 18', 17', 20', 3' 17'', 20', 18', 18', 4' 43'', 17', 20'
json: 3' 49'', 3' 13'', 17', 3' 13'', 11', 18', 18', 17', 17', 17'

Inline stats:
Mean: 936.0 s (15.60 min)
Median: 1080.0 s (18.00 min)
SD: 373.9 s (6.23 min)
SEM: 118.2 s (1.97 min)

JSON stats:
Mean: 751.5 s (12.53 min)
Median: 1020.0 s (17.00 min)
SD: 395.7 s (6.60 min)
SEM: 125.1 s (2.09 min)

Although there seems to be a small difference, it doesn’ t reach significance (Welch’s T-test: t = 1.072, p = 0.2981).

We think it’s likely that with a larger sample size, we would observe that json format are processed faster than inline, but wit have and the test we performed we can’ t draw the conclusion. Importantly, it needs to be taken into consideration that comp LLM queries can be confounded by variable resource allocation and network conditions, making them inherently noisy.

9. One major limitation of the study is the dependence on proprietary GPT models accessible only via OpenAI's API. This cons and long-term availability. Indeed, the recent release of GPT-5 already renders some of the reported results outdated. While remains highly valuable, it would be worthwhile to also evaluate local or open-source LLMs to ensure future reproducibility.

We completely agree with Reviewer 1. We now have included open-weight models in our analysis and manuscript, and we als recent GPT models (GPT-4.1 and GPT-5-mini) to make the study more current.
To our surprise, we found that several open-weight models performed similarly to proprietary GPT models in both biome and accuracy, while other models were less performant. We found that Qwen3-Next-80B-A3B-Instruct achieved comparable or ev accuracy than GPT-4.1 and GPT-5-mini, while models such as microsoft-phi-4 also demonstrated strong, consistent performa
We also found that Qwen embedding models (also open-weight) consistently outperformed OpenAI’s text-embedding-3-small
In our revised discussion we report a cost comparison (open-weight models were typically 10–50 times lower than proprietar also clarify why there is a cost to begin with: although open-weight models are freely available, running them locally requires resources, and at present it is often more economical to use third-party hosting platforms (e.g., DeepInfra) than to purchase site. (638-647)
We are particularly glad to have conducted this analysis in response to Reviewer 1’s suggestion, as it delivers a strong messa annotation is no longer restricted to commercial APIs. Open-weight models can be run repeatedly to build consensus across p confidence in the annotations. Besides the substantial cost reductions, the use of open-weight models enhances transparency long-term sustainability. Overall, this additional analysis proved well worth the effort, as it strengthened the manuscript both conceptually by demonstrating that high-quality, reproducible metadata annotation can now be achieved with open tools.

Reviewer #2: Reproducibility report for: Enhanced semantic classification of microbiome sample origins using Large Languag
Journal: Gigascience
ID number/DOI: GIGA-D-25-00316
Reviewer(s): Laura Caquelin, Department of Clinical Neuroscience, Karolinska Institutet, Sweden [Wrote the report and repro

----

1. Summary of the Study

This study evaluates whether Large Language Models (LLMs) can help re-annotate sequencing records. Using GPT models, th
scalability, time, cost, and performance against a benchmark of 1,000 hand-curated examples. They then applied this approa
environmental sequencing records, producing standardized annotations.

----

2. Scope of reproducibility

According to our assessment the primary objective is: to evaluate how closely GPT's annotation performance approached tha
classifying environmental sequencing samples into biomes and sub-biomes.

- Outcome: Accuracy of biome and sub-biome classification compared against a hand-curated benchmark dataset.

- Analysis method outcome: As described to validate biome classifications: "For paired comparisons of repeated sample IDs,
test, which is appropriate for paired binary outcomes (True/False)", while "for comparisons across different sample sets, we e
independent samples. In both scenarios, a Bonferroni correction is applied to adjust for multiple comparisons".

For sub-biomes, comparisons across different sets were performed with independent t-tests, while "for runs involving the san
comparisons are performed using the paired t-test". Section "Validation statistics" page 13-14.

- Main result: "The improvement in accuracy between GPT's initial classification and the human's performance with the impro
statistically significant (adj p-value=0.031), but also between the human's attempt first attempt (with the initial prompt) and
attempt (better prompt) (adj p-value≤0.001). No significantly different performances were detected for the sub-biome classi
GPT and the human, nor between prompt versions (adjp-value=1)." Section "Human versus GPT classification accuracy" page

----
3. Availability of Materials

a. Data

- Data availability: Open
- Data completeness: Complete = all data necessary to reproduce main results are available
- Access Method: Repository
- Repository: https://zenodo.org/records/16100607
- Data quality: Complete but no metadata associated with the file

Yes, that is correct, we did not add the file to the Zenodo repository. Instead we only put it on the Github repo. The file is cal
"MicrobeAtlasProject_files_list_Zenodo.tsv". In the new Zenodo upload (link reported in the revised manuscript), we now incl

b. Code

- Code availability: Open
- Programming Language(s): Python
- Repository link: https://github.com/GaioTransposon/metadata_mining/tree/main
- License: CC0
- Repository status: Public
- Documentation: Readme file clear but require one modification

Yes, that is correct. We updated the README.md file to avoid confusing users into making ~/MicrobeAtlasProject/MicrobeAtla
the "Requirements" section of the README.md file.

----

4. Computational environment of reproduction analysis

- Operating system for reproduction: MacOS 15.6.1
- Programming Language(s): Python
- Code implementation approach: Using shared code
- Version environment for reproduction: Python 3.13.7

----

5. Results

5.1 Original study results

- Results:

"The improvement in accuracy between GPT's initial classification and the human's performance with the improved prompt wa
significant (adj p-value=0.031), but also between the human's attempt first attempt (with the initial prompt) and the human'
(better prompt) (adj p-value≤0.001). No significantly different performances were detected for the sub-biome classification,
and the human, nor between prompt versions (adjp-value=1)."

(The authors identified an error in the manuscript text during the review. Therefore, the following part of the manuscript nee
email exchange with the authors below).

5.2 Steps for reproduction

-> Run the two first scripts of the container 4 in the Github: validate_biomes_subbiomes.py and overall_analysis.py

- Issue 1: The README instructions for setting up the ~/MicrobeAtlasProject directory can lead to a nested folder structure
(~/MicrobeAtlasProject/MicrobeAtlasProject) if followed literally. This causes the Docker container to fail when attempting to a
gpt_file_label_map.tsv, since they are not found at the expected path /MicrobeAtlasProject/.

-- Resolved: The issue was resolved by manually renaming and flattening the directory structure after extraction, ensuring th
MicrobeAtlasProject_Zenodo are directly placed inside ~/MicrobeAtlasProject/. However, the current instructions can mislead
in the README would be helpful.

Yes, that is correct. We updated the README.md file to avoid confusing users into making ~/MicrobeAtlasProject/MicrobeAtla
the "Requirements" section of the README.md file.

- Issue 2: During the execution of the overall_analysis.py script, multiple files with the same label were found, requiring man
to use for the analysis.

-- Resolved: The manuscript does not specify which file should be selected to reproduce the results, leading to potential ambi
chose the most recent file among the options, assuming it reflects the final data version used in the manuscript. It would be l
documentation or manuscript explicitly stated this to ensure exact reproducibility.

This was a very good point. We now edited overall_analysis.py to not prompt the user to choose from any "double labeled" fi
because multiple GPT outputs shared identical parameters but different timestamps. As reviewer 2 suggested this caused unn
The solution we found was to remove the unnecessary GPT output files from the Zenodo repository (now reflected in the curr

-> Compare the results reproduced to the results presented in the manuscript

- Issue 3: The results obtained by running validate_biomes_subbiomes.py are two files: biome_subbiome_results.csv and
biome_subbiome_stats.csv, which contain a large amount of output (1,284 and 48,197 rows respectively). The script overall_
overall performance metrics in the terminal output, but does not produce the adjusted p-values relevant to the scope of this r

-- Unresolved: It was difficult to identify where to find the results presented in the manuscript, so an email was sent to the au

Yes, this was a main issue which has now been resolved. Those fairly large files (1,284 and 48,197 rows) were caused by the an existing file, as mentioned in our correspondence. Now the script validate_biomes_subbiomes.py has been updated to crea biome_subbiome_results*.csv and biome_subbiome_stats*.csv files. There was additionally a problem with the script, that ar editing the code for Docker (after our first submission and under request of the Editor). Without us realizing, the code: 1. sile fields of biome_subbiome_results.csv and biome_subbiome_stats.csv even when embeddings .json files were missing; 2. did properly, so it did a comparison of everything against everything, generating an unnecessarily large file, very hard to read.
The removal of the 20 gpt output files caused some values to slightly drift. Here we list the changes (please note that the pag refer to the previous manuscript version):
- Line 395: "(precision: 81.7%, F1-score: 80.16)." → "(precision: 82.0%, F1-score: 80.34)."
- Line 396: "n=125" → "n=105" (this is the current number of files)
- Line 397: "F1-score: 76.1%" → "F1-score: 76.4%"
- Line 400: "accuracy rates of 93.6% and 94.3%" → "accuracy with accuracy rates of 94.6% and 93.8%"
- Line 401: "In contrast, 'plant', 'water', and 'other' biome samples exhibit lower accuracy rates (67.2%, 79.3%, and 67.5%, contrast, 'plant', 'water', and 'other' biome samples exhibit lower accuracy rates (67.3%, 79.9%, and 67.2%, respectively)"
- Line 405: "precision (81.7%" → "precision (81.9%"
- Line 406: "accuracy: 93.6%" → "accuracy: 93.8%"
- Line 407: "precision: 87.8% vs. 8.24%" → "precision: 87.8% vs. 82.4%" (this was a typo)
- Lines 408-411: "While both methods struggle with plant samples, which they often misclassified as soil (Fig. 2A-B), GPT aga misclassifications on this subset (4.0% vs. 17.8%). In addition, GPT shows improved predictions in the reverse direction by m less frequently as plant biome, compared to the keyword-based classifier (1.3% vs. 11.5%)." → "Both methods struggle with they often misclassified as soil (Fig. 2A-B). Whereas GPT most often misclassified them as 'soil' (27.0%), the keyword-based the misclassified samples into either 'soil' (16.8%) or 'other' (17.8%). " (here the values are updated as well as the phrasing
- Line 413: "as indicated by Cohen's kappa values (GPT: 0.757" → "as indicated by Cohen's kappa values (GPT: 0.760"
- Line 420: "chi-squared" → chi-squared: 7482
- Line 421: "A large fraction of misclassified 'animal' samples (49.9%) are erroneously predicted as 'water'." → "A large fracti 'animal' samples (53.0%) are erroneously predicted as 'water'."
- Lines 420-423: "misclassifications within 'plant' (82.9%) and 'water' samples (59.1%) are 'soil'" → "misclassifications withir 'water' samples (59.0%) are 'soil'"
- Lines 424-425: "skewness: 1.32; kurtosis: 0.23" → "skewness: 1.34; kurtosis: 0.27"
- Lines 426-427: "Notably, 23 samples (5% of the total) fall at or above the 95th percentile, with 109 or more misclassificatio samples (5.3% of the total) fall at or above the 95th percentile, with 90 or more misclassifications"
- Lines 428-430: "Across 117 independent classification runs using different parameter settings, the average number of miscl is 26.0 (SD: 36.0)." → "Across 105 independent classification runs using different parameter settings, the average number of run is 21.7 (SD: 29.9)."
- Lines 430-431: "Half of the samples are misclassified 5 times or fewer, and three-quarters have no more than 41 misclassif samples are misclassified 4 times or fewer, and three-quarters have no more than 34 misclassifications"
- Line 432: "The maximum observed was 117" → "The maximum observed was 97"
- Line 436: "SRS4776621" → "SRS5304049" (this is another rhizosphere sample that popped in the updated results - the san sample: it was misclassified as soil by GPT in all but one case, as mentioned at the end of the paragraph).
- Line 438: "was incorrectly assigned to 'water' by GPT in 109 out of 117 instances" → "was incorrectly assigned to 'water' by instances"
- Line 441: "assigned to 'animal' by GPT in 109 out of 112 instances" → "assigned to 'animal' by GPT in 90 out of 93 instance
- Line 522: "accuracy was mitigated (freqp 0.0: 80.76%; freqp 2.0: 80.96%)" → "accuracy was mitigated (freqp 0.0: 79.16%
- Lines 537-553: we worded this part about the replicates better to clarify that the lower accuracy ranges seen across the syr the async requests are due to the format difference and not the diff between sync and async. Sync versus async are the 6 re results we clarify in the last paragraph.

As we refreshed the gpt files (removed the unnecessary ones) the geographic location analysis values also changed. At that p many more samples available from the production run (unique ones), we updated the section "Geographic location prediction include all samples (990,172 instead of the previous 130,689). NB: these are not over 2M because it's a merge between sam geographically annotated by GPT and samples from which metadata we could retrieve the coordinates, hence 990k only. We section to have shifted: 95.32% vs 95.94% matches, now versus before.
As we did not re-perform the manual-picking inside done in geo_check.py (picking manually 100 samples metadata and decic why - gpt vs coordinates), we could use the same dictionary that was created earlier (it's an argument of geo_check.py). The changed is the sample size of the analysis, and therefore also the map figures (now Figure 7 and Suppl. Figure 8).

Message sent by the authors

Dear reviewer,

By running validate_biomes_subbiomes.py as described (using gpt_file_label_map.tsv as --map_file), the output will be two named biome_subbiome_results.csv and biome_subbiome_stats.csv.

The latter file will contain the stats (hence the adjusted p-values). Were you able to reproduce such files?

We did notice there are a few mistakes.

Mistake 1.

In the manuscript it says:

" A trained molecular biologist, with no prior exposure to the project, was given the same prompt instructions as GPT and wa sample biomes and sub-biomes. While against the benchmark dataset, GPT achieved an accuracy of 79.76% (n=499; SD=40 annotator reached 78.0% (n=250; SD=33.0). "

The second standard deviation should be replaced with SD=42.0.

We re-confirm this change.

Mistake 2.

In the manuscript it says:

" The improvement in accuracy between GPT's initial classification and the human's performance with the improved prompt w significant (adj p-value=0.031), but also between the human's attempt first attempt (with the initial prompt) and the human's prompt) (adj p-value≤0.001). "

The first adjusted p-value should not be 0.031 but 0.134 hence not significant so this sentence should be adjusted to:

" There was an improvement in accuracy between the human's first attempt (with the initial prompt) and the human's second prompt) (adj p-value≤0.001). "

We re-confirm this change.

Mistake 3.

We built on biome_subbiome_results.csv and biome_subbiome_stats.csv further than necessary so the two files on Zenodo sh avoid confusion. This was a problem of the script validate_biomes_subbiomes.py which concatenates on existing files (e.g.: biome_subbiome_results.csv and biome_subbiome_stats.csv) instead of creating new ones. We should probably procee two files with the files without repetitions.

We thank you for your work and please do let us know if everything works out now.

Thank you and kind regards,

We re-confirm this change. Yes, the new Zenodo link has the correct files. The script (as mentioned above) now does not con files anymore, but produces fresh ones when re-run.

####

The authors confirm that the results presented in the manuscript can be found in the biome_subbiome_stats.csv file. This file

According to the authors, the file includes data from both existing files and new data. As a result, it is difficult to determine w
reproduced. Even when using a Ctrl+F search for the reported p-value (e.g, pvalue = 0.134) in the Excel file, this value appe
labeled under different configurations such as (label1/label2):
--- chunk_size3000/sync_chunkN_presp0.0;
--- chunk_size3000/sync_chunkN_temp1.5;
--- chunk_size5000/gpt4-0613;
--- machine/ sync_chunkY_topp0.0, etc…

Yes, as mentioned above we solved this problem by 1. Replacing the correct biome_subbiome_results*.csv and biome_subbio
De-bugging validate_biomes_subbiomes.py. When we edited our scripts to work with Docker, some issues had arisen that we
was the script silently filling out empty fields of biome_subbiome_results.csv and biome_subbiome_stats.csv even when emb
missing. Another issue was not properly grouping by test_type properly, so the output was very hard to read. The strange lab
noticed, no longer happens now. Only files falling under the same column "test_type" (in gpt_file_label_map.tsv file), are con
wouldn't make sense to compare for example "chunk_size3000" with "sync_chunkN_presp0.0". This is also why the biome_su
was so large. We thank Reviewer 2 for spotting this important problem.

5.3 Statistical comparison Original vs Reproduced results

- Results: The biome_subbiome_stats.csv file was reproduced, but it is difficult to distinguish between the newly reproduced
data already present in the file. Additionally, the data presented in the manuscript are also hard to identify due to the size of
was performed.

Biome_subbiome_stats*.csv (the * now stands as we have three files each, after the addition of the new analysis with the op
only produces the correct comparisons, and there is no concatenation to the existing file. With the file now is being concise, w
overcome the problem of poor readability. We stay open to further improvements where found necessary.

- Comments: -
- Errors detected: Authors identified an error in the manuscript text during the review with the first adjusted p-value that is n

We re-confirm this change.

- Statistical Consistency: No comparison was performed.
----

6. Conclusion

- Summary of the computational reproducibility review

The main scripts to reproduced the results were successfully executed and the output files were generated. However, due to t
files and the lack of precise references in the manuscript, it was difficult to identify which parts of the output correspond to th
the paper. Moreover, authors mentionned that the script adds data to existing output files rather than generating new ones,
distinguish between old and new data. This led to confusion when trying to compare the reproduced results with those in the
comparison of statistical values was not possible.

- Recommendations for authors

To improve the reproducibility of the manuscript, we recommend the authors to:

-- Clarify instructions in the README about the MicrobeAtlasProject folder.

Done.

-- Ensure scripts generate new outputs or clarify which data is new vs. existing in the files with for example a column indicati
"new" or "existing").

Done. It now generates a new output at each run. No more concatenation.

-- Link results in the manuscript to specific rows/sections in the output files to easily locate the exact data used. Another solu consider including a smaller, or a filtered version of the output files with only the rows used for key reults, figures or tables, t easier and avoid error.

This is a good suggestion by Reviewer 2, but we were wondering whether the addition in the manuscript of "see line xx of file values or stats are reported, would not make it much heavier to read. We believe now we have significantly reduced the size values are easier to read. Also correcting the issue of random labels being compared to random labels, has solved a major pr values are easier to find. The results file is now 154 lines long (instead of 1,284) and the stats file now has 1,172 lines (inste worth noting that the new files also include the new output files generated for the comparison of OpenAI with open-weight m under request of Reviewer 1).

-- Metadata: For the data used or generated by the scripts, it would be helpful to include accompanying metadata files that e
--- The definition of each variable name.
--- The origin of each dataset (raw, processed, etc).
--- Any preprocessing steps applied before analysis.

Yes, this file is "MicrobeAtlasProject_files_list_Zenodo.tsv", which we previously uploaded to our Github repo, but not on the showed on the front page of the Zenodo repository, but it's not downloadable. In the new Zenodo version we now include the the file downloadable, we expanded the descriptions for each file, as Reviewer 2 suggested.

Reviewer #3: The ability to reuse scientific data for secondary analysis is an extremely important topic. Since the promotion Principles a decade ago, the central importance of standards-adherent metadata has received considerable attention. Althoug doesn't mention the FAIR principles, the work is important in understanding what it takes to make datasets FAIR and "AI rea

We thank Reviewer 3 for this valuable comment and for emphasizing the connection between our work and the FAIR principle study directly contributes to advancing dataset FAIRness and AI readiness through large-scale metadata standardization usin have revised the manuscript to explicitly acknowledge and discuss this connection with FAIR. We did so in the Abstract (line Results (line 386) and Discussion (lines 722, 878).

A core problem with the paper is that it is unsure who its audience is. The paper is motivated by the needs to scientists to se online datasets for secondary analysis, but much of the paper concerns highly technical issues that are related to fine tuning laudable that the manuscript annotates its discussion of the authors' methods with pointers to actual Python scripts that wou replicate the authors' work. The detailed presentation, however, may make it hard for many readers to understand the comp all the scripts are implementing. The organization of the paper weaves from discussions of the ability of LLMs to extract scien "legacy" experimental metadata to details of how to enhance computational efficiency to make the use of LLMs more cost-eff abstract of the paper suggest that the authors are aiming for a more scientific audience, but much of the manuscript focuses implementation details that will be less important to such readers.

We thank Reviewer 3 for the thoughtful and constructive comment, and we appreciate the recognition of our efforts to make transparent and reproducible. Our goal with this manuscript is indeed to reach several audiences:
(1) computational researchers or bioinformaticians who wish to reproduce or extend the pipeline, and
(2) scientists from molecular biology, microbiology, or ecology who regularly encounter poorly structured metadata and wish LLMs have progressed toward assisting human curation, and
(3) scientists from molecular biology, microbiology, or ecology who operate at the intersection of experimental and computat
We acknowledge that bridging these communities is ambitious, but it reflects the evolving nature of biological data science, w increasingly operate at the intersection of experimental and computational fields. At present, there is no broadly adopted solu widespread problem of non-standardized or effectively unusable metadata in public repositories. Our work aims to fill this gap conceptual guidance and a reproducible, step-by-step framework for improving metadata usability through LLM-based re-ann
At the same time, we acknowledge that the manuscript provides full transparency at the cost of lots of detail (for example ex script does).Users that do not want to extend or modify the scripts will perhaps not need that level of verbosity and granulari README file in the GitHub repo walks step-by-step through reproducing the pipeline.
To make the paper easier to follow for non-technical readers, we have now added an overview paragraph at the start of the R summarizing the logical flow of analyses and highlighting how each component connects to the study's central question (lines helps maintain orientation through the more detailed methodological subsections that follow.

Missing from the paper is a detailed discussion of what the metadata in SRA are really like. The reader never sees complete e
metadata that are processed in the authors' work, and thus it is hard to have intuition about the problem that the authors are
particular, the paper doesn't present information about the range of attributes in user-defined metadata fields in SRA. The pa
a discussion of the structure of scientific metadata in general, and of how the authors' work fits into the larger effort in the re
make datasets FAIR. (Full disclosure: My own laboratory is involved in such activity. See https://arxiv.org/abs/2504.05307v2

We thank Reviewer 3 for this valuable comment and for sharing their reference, which we have cited in the revised manuscrip
showing concrete examples of metadata structure helps readers better appreciate the challenges addressed by our work. To t
expanded the Introduction to include a discussion of typical metadata inconsistencies, now citing Ikeda et al. (2025), who doc
BioSample entries, as well as our own representative metadata examples (added as Supplementary Figure 1). These addition
into the heterogeneity and irregularities of BioSample metadata, highlighting why automated interpretation is required.
Regarding the "range of attributes in user-defined metadata fields in SRA," this topic is addressed in the Results section (Info
fields), which details the diversity of fields used to describe sample origin, and is further supported by Supplementary Figure
additions provide both a qualitative and quantitative view of the metadata landscape.
Finally, we note that while a deeper exploration of the metadata problem itself would indeed be valuable, we had to balance s
given that the primary focus of the manuscript is the evaluation of GPT and open-weight LLM performance for metadata re-an
version now clarifies the metadata challenges motivating this task while keeping the main emphasis on methodological evalua
performance.
The abstract of the paper states that the authors "test to what extent LLMs can be used to cost-effectively automate the re-a
records." Alas, the paper really examines re-annotation of only the fields for "biome" and "location." A weakness of the paper
doesn't learn what other fields may be relevant in these metadata records, and why the authors chose to focus on the particu
studied. Overall, much more attention should be placed on discussion of the limitations of the work and how well the results r
general problems in standardization of scientific metadata.

We agree that our analysis focuses on a subset of metadata fields and that this limitation should be explicitly acknowledged.
we have added a paragraph to the Discussion describing this scope, clarifying that our validation was restricted to fields relat
namely biome, sub-biome, and geographic location. These attributes were chosen because they are central to the MicrobeAtla
as the empirical foundation for this study, and because they apply broadly to all sequencing samples regardless of context. T
most inconsistently reported fields in public repositories, making them particularly relevant for testing LLM-based re-annotati
Other metadata fields (e.g., environmental parameters such as pH, altitude, or ocean depth; or methodological fields such as
and library type) are indeed important for downstream analyses but were beyond the scope of this work. Many of these are m
highly structured and can be parsed more reliably using conventional NLP or rule-based methods, whereas the biome and sub
nuanced semantic interpretation—an area where LLMs can add the most value.
The newly added section in the Discussion explicitly acknowledges this as a limitation of the current study and frames it as a
comprehensive LLM-driven metadata standardization. We emphasize that the framework presented here can, in principle, be
metadata fields in future work.

Minor comments:

The term "biome" is never well defined.

We acknowledge a proper biome definition was missing earlier on. In the revised version we added the definition to the introd
methods (lines 174-194); and results section (lines 388-390).

Frequently, parenthetical remarks begin with "e.g." and end with "etc." This style is redundant; you need only one of these a
instance.

Good point. We corrected this.

Page 6, para 1: "last three digits" or "last three characters"?

We relied on the "last three digits" because all sample IDs terminate with digits, whether they are SRS, ERS or DRS. For exan
ERS3774001, DRS005001. Using these digits to define directory names provided a simple and deterministic way to distribute
files across subdirectories. This structure was essential for filesystem performance and scalability: storing millions of files in a
dramatically slow down file access, indexing, and I/O operations. By splitting the files into subdirectories named after the last

ensured that each folder contained a manageable number of files, enabling faster read/write operations and easier parallel pr
benefits both our own workflow as well as any user intending to reproduce the pipeline.

What is the motivation for consolidating reference ontologies into a single dictionary?

This was primarily a practical and logistic choice. By consolidating them into one, users who want to reproduce the pipeline ca
use our pre-made dictionary (downloadable from the zenodo link in the revised manuscript), or even compare the checksum
theirs to know if it differs (simpler and more transparent with one unified file than with many smaller ones). Also, a single co
ensures consistent ontology mapping across the entire pipeline. It eliminates potential discrepancies that could arise if indivi
updated independently or parsed in different orders. In this way, lookup and caching during annotation are faster, since the s
unified key–value structure into memory rather than repeatedly querying multiple smaller files.

Page 13, para 2: The notion of "lenient matches" requires much more discussion. If the goal is to make the legacy metadata
then a "lenient" match would not seem to be valid. The operative question is, "What metadata terms will users invoke to sear
presumably users will be searching for standard terms only.
We observed that, despite strict prompt instructions to output only one of the five allowed biome labels ("animal", "plant", "w
LLMs occasionally appended short clarifying text such as "animal (human)" or "water (marine)". These are still fully valid bior
somewhat more specific—but are not exact string matches to the benchmark. To quantify performance in these cases, we de
an output where one of the five biome labels appears as a complete word (e.g., "animal (human)" counts as a lenient match
not include partial or fuzzy matches (e.g., "plan" ≠ "plant"). This criterion was only applied when assessing biome classificati
highlight the model's tendency to add detail when certain creativity parameters (i.e.: frequency penalty >0.25) were used du
(Figure 6).These are the corresponding files:
gpt_clean_output_nspb100_chunkingyes_chunksize2000_modelgpt-3.5-turbo-1106_temp1.0_maxtokens4096_topp0.75_freqp0.25_presp1.5_rs22_API118_normal_dt202406051326.txt
gpt_clean_output_nspb100_chunkingyes_chunksize2000_modelgpt-3.5-turbo-1106_temp1.0_maxtokens4096_topp0.75_freqp0.0_presp1.5_rs22_API120_normal_dt202406051442.txt
gpt_clean_output_nspb100_chunkingyes_chunksize2000_modelgpt-3.5-turbo-1106_temp1.0_maxtokens4096_topp0.75_freqp1.0_presp1.5_rs22_API132_normal_dt202406051450.txt
gpt_clean_output_nspb100_chunkingyes_chunksize2000_modelgpt-3.5-turbo-1106_temp1.0_maxtokens4096_topp0.75_freqp2.0_presp1.5_rs22_API153_normal_dt202406051500.txt

In the production run (no chunking used), where we processed over 2M samples, we never obtained extra info to the biome c
Please see below:

```
$ awk -F'\t' '{count[$2]++} END {for (b in count) print b, count[b]}' GPT_biomes.txt
plant 185706
other 85324
animal 1241645
air 10
food 616
water 305883
coral 1
soil 237225
```

The GPT_biomes.txt file, as well as other parsed GPT output files are now downloadable on the new Zenodo link (in the revise
In conclusion, the lenient match metric serves purely as an analytic tool to understand how parameter changes affect model
relaxation of standards for metadata annotation. In practical terms, lenient matching ensures that variants like "animal (hum
under the canonical "animal" biome for evaluation, which reflects how such records would ultimately be indexed and retrieve
standard biome terms.
We have now clarified in the Methods (lines 302-309) what we mean by 'lenient match,' emphasizing that it refers only to ap
(e.g., 'animal (human)') and is used solely for benchmarking, not for metadata standardization.

Page 17, para 3: It's unclear what is meant by "most samples have fewer misclassifications." Fewer than what?

That is a good point. It needs clearer wording as it's comparative. We changed it from:

< The distribution of misclassifications per sample is right-skewed (skewness: 1.34; kurtosis: 0.27), indicating that while mo
misclassifications, a small subset exhibit disproportionally high values (Supplementary Figure 4). >
to:
< The distribution of misclassifications per sample is right-skewed (skewness: 1.34; kurtosis: 0.27), indicating that most sam
misclassifications compared to a small subset with disproportionately high values (Supplementary Figure 4). >

Page 24, para 2: It's not clear what you mean when you say, "In half of the cases GPT correctly predicted the location, while
parsed from the metadata were incorrect." Are you saying that GPT gave correct results when the lat/lon data were incorrect

In short, yes. GPT extracts the geo location in text form (e.g.: if the metadata reports "Orosei, Italy" or just "Orosei", it will r
because the prompt instructs the LLM to "infer geographical location where the sample was collected, including the country (
"In half of the cases" refers to half of the pool of 100 misclassified samples. These 100 misclassified samples were picked ran
discrepancy of more than 1000 km between GPT predicted geo location and the metadata derived coordinates).

Half of these (n=50) GPT guessed the location in text form correctly, while the metadata extracted coordinates were incorrec
explaining why the coordinates were erroneous. This is still based on the 100 misclassified samples we manually observed to
underlying causes of these discrepancies might be.
It is worth noting that, in our broader dataset, a surprisingly large fraction of entries contain evidently incorrect latitude/long
swapped coordinates, truncated decimals, misplaced signs, or mismatched hemispheres. These inconsistencies often arise fro
or from metadata formatting changes during submission to public repositories. In this context, the ability of LLMs to cross-va
information based on textual cues offers an additional layer of quality control, flagging cases where numeric coordinates may
declared sampling site.

Figure 1 is very busy and the tiny font is hard to read.

We updated figure 1 and increased the font.

Editorial Manager®

Cookie Preference Center

We use cookies which are necessary to make our site work. We may also use additional cookies to analyze, improve and personalize our content and your digital experience. For more information, see our Cookie Policy.

You may choose not to allow some types of cookies. However, blocking some types may impact your experience of our site and the services we are able to offer. See the different category headings below to find out more or change your settings.

Allow all

Manage Consent Preferences

Strictly Necessary Cookies

Always active

These cookies are necessary for the website to function and cannot be switched off in our systems. They are usually only set in response to actions made by you which amount to a request for services, such as setting your privacy preferences, logging in or filling in forms. You can set your browser to block or alert you about these cookies, but some parts of the site will not then work.

Functional Cookies

These cookies enable the website to provide enhanced functionality and personalisation. They may be set by us or by third party providers whose services we have added to our pages. If you do not allow these cookies then some or all of these services may not function properly.

Performance Cookies

These cookies allow us to count visits and traffic sources so we can measure and improve the performance of our site. They help us to know which pages are the most and least popular and see how visitors move around the site.

Targeting Cookies

These cookies may be set through our site by our advertising partners. They may be used by those companies to build a profile of your interests and show you relevant adverts on other sites. If you do not allow these cookies, you will experience less targeted advertising.

Cookie List

Clear

Apply Cancel

Consent Leg.Interest

Confirm my choices

Powered by onetrust
