# Supplementary material for: Enhanced semantic classification of microbiome sample origins using large language models (LLMs)
Source: Gigascience. 2026 Feb 12;15:giag015. doi: 10.1093/gigascience/giag015 (PMC13042274; doi:10.1093/gigascience/giag015)
Supplement: giag015_Reviewer_1_Report_original_submission — Reviewer 1 -- 9/5/2025 [file giag015_reviewer_1_report_original_submission.pdf]

Reviewer Report

Title: Enhanced semantic classification of microbiome sample origins using Large Language Models (LLMs)

Version: Original Submission **Date:** 9/5/2025

Reviewer name: Reviewer 1

Reviewer Comments to Author:

The manuscript presents a carefully executed study using non-finetuned GPT models to classify microbiome sample metadata. It is very well written, and both the analyses and the interpretations are generally sound. I found the evaluation thorough and the presentation clear.

1. The study provides a detailed evaluation of LLM-based metadata curation, clearly advancing over keyword-based approaches. However, it is surprising that recent related studies using LLMs for metadata curation are not cited. For completeness, I suggest including references such as:
- https://doi.org/10.1093/gigascience/giaf070 (disclaimer: I am an author of the paper. You might like the table 3),
- https://doi.org/10.1093/bib/bbad535,
- https://doi.org/10.3897/phytokeys.261.158396,
- https://pmc.ncbi.nlm.nih.gov/articles/PMC12099408/.

2. The scale of the processed data is impressive. However, there appears to be a discrepancy: the Zenodo repository file metadata.out contains 2,254,619 accession IDs (presumably the input), while the GPT output files (gpt_clean_output) include only around 1,000 samples (presumably the benchmark dataset), whereas the manuscript states that 3.8 million samples were processed. It would be helpful to clarify these numbers and, if applicable, explain why fewer outputs are provided. I also recommend reorganizing the Zenodo repository so that readers can download individual files rather than the entire large archive.
3. The data processing pipeline on GitHub is very useful. The repository currently indicates a CC0 (public domain) license. Since CC0 is typically intended for datasets rather than source code, please clarify whether this was intentional or if a software-specific license (e.g., MIT, Apache 2.0) would be more appropriate.
4. A different typeface appears in some paragraphs (e.g., pp. 19 and 21). Please check whether this was intentional.
5. The finding that grouping 5-17 samples per request does not substantially affect accuracy is interesting. Given that GPT models often fail with counting or item listing, the observed quality decline with larger chunk sizes seems reasonable and aligns with expectations.
6. On p. 26, the observed variability in field usage may be linked to the BioSample package system used for submission (see: https://www.ncbi.nlm.nih.gov/biosample/docs/packages/). Some fields, such as env_biome and env_feature, were once mandatory for environmental samples but are currently optional, I suppose. Such historical changes may partly explain biases in field usage.
7. The manuscript appropriately highlights the presence of ambiguous or unresolvable sample

descriptions. We reached a similar conclusion in our own work with local LLMs: in many cases, even expert curators cannot determine a "correct" label, and the right answer may depend on context or application.

8. The observation that JSON output significantly improves sub-biome classification accuracy is intriguing and consistent with our internal experience with local LLMs. Since output format may also affect processing speed, it would be useful to report whether response times differed between JSON and inline formats.

9. One major limitation of the study is the dependence on proprietary GPT models accessible only via OpenAI's API. This constrains reproducibility and long-term availability. Indeed, the recent release of GPT-5 already renders some of the reported results outdated. While the present study remains highly valuable, it would be worthwhile to also evaluate local or open-source LLMs to ensure future reproducibility.

Methods

Are the methods appropriate to the aims of the study, are they well described, and are necessary controls included? Choose an item.

Conclusions

Are the conclusions adequately supported by the data shown? Choose an item.

Reporting Standards

Does the manuscript adhere to the journal's guidelines on minimum standards of reporting? Choose an item.

Choose an item.

Statistics

Are you able to assess all statistics in the manuscript, including the appropriateness of statistical tests used? Choose an item.

Quality of Written English

Please indicate the quality of language in the manuscript: Choose an item.

Declaration of Competing Interests

Please complete a declaration of competing interests, considering the following questions:

- Have you in the past five years received reimbursements, fees, funding, or salary from an organisation that may in any way gain or lose financially from the publication of this manuscript, either now or in the future?
- Do you hold any stocks or shares in an organisation that may in any way gain or lose financially from the publication of this manuscript, either now or in the future?
- Do you hold or are you currently applying for any patents relating to the content of the manuscript?
- Have you received reimbursements, fees, funding, or salary from an organization that holds or has applied for patents relating to the content of the manuscript?
- Do you have any other financial competing interests?
- Do you have any non-financial competing interests in relation to this paper?

If you can answer no to all of the above, write 'I declare that I have no competing interests' below. If your reply is yes to any, please give details below.

I declare that I have no competing interests

I agree to the open peer review policy of the journal. I understand that my name will be included on my report to the authors and, if the manuscript is accepted for publication, my named report including any attachments I upload will be posted on the website along with the authors' responses. I agree for my report to be made available under an Open Access Creative Commons CC-BY license (http://creativecommons.org/licenses/by/4.0/). I understand that any comments which I do not wish to be included in my named report can be included as confidential comments to the editors, which will not be published.

Choose an item.

To further support our reviewers, we have joined with Publons, where you can gain additional credit to further highlight your hard work (see: https://publons.com/journal/530/gigascience). On publication of this paper, your review will be automatically added to Publons, you can then choose whether or not to claim your Publons credit. I understand this statement.

Yes Choose an item.
