# Supplementary material for: Enhanced semantic classification of microbiome sample origins using large language models (LLMs)
Source: Gigascience. 2026 Feb 12;15:giag015. doi: 10.1093/gigascience/giag015 (PMC13042274; doi:10.1093/gigascience/giag015)
Supplement: giag015_Reviewer_2_Report_original_submission — Reviewer 2 -- 9/10/2025 [file giag015_reviewer_2_report_original_submission.pdf]

Reviewer Report

Title: Enhanced semantic classification of microbiome sample origins using Large Language Models (LLMs)

Version: Original Submission **Date:** 9/10/2025

Reviewer name: Reviewer 2

Reviewer Comments to Author:

Reproducibility report for: Enhanced semantic classification of microbiome sample origins using Large Language Models
Journal: Gigascience
ID number/DOI: GIGA-D-25-00316
â€‹â€‹â€‹Reviewer(s): Laura Caquelin, Department of Clinical Neuroscience, Karolinska Institutet, Sweden [Wrote the report and reproduced the results]
----
1. Summary of the Study
This study evaluates whether Large Language Models (LLMs) can help re-annotate sequencing records. Using GPT models, the authors tested scalability, time, cost, and performance against a benchmark of 1,000 hand-curated examples. They then applied this approach to million environmental sequencing records, producing standardized annotations.
----
2. Scope of reproducibility
According to our assessment the primary objective is: to evaluate how closely GPT's annotation performance approached that a human expert when classifying environmental sequencing samples into biomes and sub-biomes.
- Outcome: Accuracy of biome and sub-biome classification compared against a hand-curated benchmark dataset.
- Analysis method outcome: As described to validate biome classifications: "For paired comparisons of repeated sample IDs, we use the McNemar test, which is appropriate for paired binary outcomes (True/False)", while "for comparisons across different sample sets, we employ the t-test for independent samples. In both scenarios, a Bonferroni correction is applied to adjust for multiple comparisons".
For sub-biomes, comparisons across different sets were performed with independent t-tests, while "for runs involving the same sample IDs, comparisons are performed using the paired t-test". Section "Validation statistics" page 13-14.
- Main result: "The improvement in accuracy between GPT's initial classification and the human's performance with the improved prompt was statistically significant (adj p-value=0.031), but also between the human's attempt first attempt (with the initial prompt) and the human's second attempt (better prompt) (adj p-valueâ‰¤0.001). No significantly different performances were detected for the sub-biome classification, neither between GPT and the human, nor between prompt versions (adjp-

value=1)." Section "Human versus GPT classification accuracy" pages 18-19.
----
3. Availability of Materials
a. Data
- Data availability: Open
- Data completeness: Complete = all data necessary to reproduce main results are available
- Access Method: Repository
- Repository: https://zenodo.org/records/16100607
- Data quality: Complete but no metadata associated with the file
b. Code
- Code availability: Open
- Programming Language(s): Python
- Repository link: https://github.com/GaioTransposon/metadata_mining/tree/main
- License: CC0
- Repository status: Public
- Documentation: Readme file clear but require one modification
----
4. Computational environment of reproduction analysis
- Operating system for reproduction: MacOS 15.6.1
- Programming Language(s): Python
- Code implementation approach: Using shared code
- Version environment for reproduction: Pythonâ€¯3.13.7
----
5. Results
5.1 Original study results
- Results:
"The improvement in accuracy between GPT's initial classification and the human's performance with the improved prompt was statistically significant (adj p-value=0.031), but also between the human's attempt first attempt (with the initial prompt) and the human's second attempt (better prompt) (adj p-valueâ‰¤0.001). No significantly different performances were detected for the sub-biome classification, neither between GPT and the human, nor between prompt versions (adjp-value=1)."
(The authors identified an error in the manuscript text during the review. Therefore, the following part of the manuscript needs to be updated (see email exchange with the authors below).
5.2 Steps for reproduction
-> Run the two first scripts of the container 4 in the Github: validate_biomes_subbiomes.py and overall_analysis.py
- Issue 1: The README instructions for setting up the ~/MicrobeAtlasProject directory can lead to a nested folder structure (~/MicrobeAtlasProject/MicrobeAtlasProject) if followed literally. This causes the Docker container to fail when attempting to access required files like gpt_file_label_map.tsv, since they are not found at the expected path /MicrobeAtlasProject/.
-- Resolved: The issue was resolved by manually renaming and flattening the directory structure after extraction, ensuring that the contents of MicrobeAtlasProject_Zenodo are directly placed inside

~/MicrobeAtlasProject/. However, the current instructions can mislead users, so a clarification in the README would be helpful.
- Issue 2: During the execution of the overall_analysis.py script, multiple files with the same label were found, requiring manual selection of the file to use for the analysis.
-- Resolved: The manuscript does not specify which file should be selected to reproduce the results, leading to potential ambiguity. By default, I chose the most recent file among the options, assuming it reflects the final data version used in the manuscript. It would be helpful if the documentation or manuscript explicitly stated this to ensure exact reproducibility.
-> Compare the results reproduced to the results presented in the manuscript
- Issue 3: The results obtained by running validate_biomes_subbiomes.py are two files: biome_subbiome_results.csv and biome_subbiome_stats.csv, which contain a large amount of output (1,284 and 48,197 rows respectively). The script overall_analysis.py provides overall performance metrics in the terminal output, but does not produce the adjusted p-values relevant to the scope of this review.
-- Unresolved: It was difficult to identify where to find the results presented in the manuscript, so an email was sent to the authors.
Message sent by the authors
Dear reviewer,â€¯
By running validate_biomes_subbiomes.py as described (using gpt_file_label_map.tsv as --map_file), the output will be two .csv files namedâ€¯biome_subbiome_results.csv
andâ€¯biome_subbiome_stats.csv.â€¯
The latter file will contain the stats (hence the adjusted p-values).â€¯Were you able to reproduce such files?â€¯
We did notice there are a few mistakes.â€¯
Mistake 1.â€¯
In the manuscript it says:â€¯
" A trained molecular biologist, with no prior exposure to the project, was given the same prompt instructions as GPT and was asked to classify sample biomes and sub-biomes. While against the benchmark dataset, GPT achieved an accuracy of 79.76% (n=499; SD=40.0), the human annotator reached 78.0% (n=250;â€¯SD=33.0). "
The second standard deviation should be replaced withâ€¯SD=42.0.â€¯
Mistake 2.â€¯
In the manuscript it says:â€¯
" The improvement in accuracy between GPT's initial classification and the human's performance with the improved prompt was statistically significant (adj p-value=0.031),â€¯but also between the human'sâ€¯attemptâ€¯first attempt (with the initial prompt) and the human's second attempt (better prompt) (adj p-valueâ‰¤0.001).â€¯"
The first adjusted p-value should not be 0.031 but 0.134 hence not significant so this sentence should be adjusted to:â€¯
" There was an improvement in accuracy between the human's first attempt (with the initial prompt) and the human's second attempt (better prompt) (adj p-valueâ‰¤0.001).â€¯"
Mistake 3.â€¯

We built onâ€¯biome_subbiome_results.csv andâ€¯biome_subbiome_stats.csv further than necessary so the two files on Zenodo should be "cut" earlier to avoid confusion. This was a problem of the scriptâ€¯validate_biomes_subbiomes.py whichâ€¯concatenates on existing files (e.g.:â€¯biome_subbiome_results.csv andâ€¯biome_subbiome_stats.csv) instead of creating new ones. We should probably proceed by replacing these two files with the files without repetitions.â€¯
We thank you for your work and please do let us know if everything works out now.â€¯
Thank you and kind regards, ####
The authors confirm that the results presented in the manuscript can be found in the biome_subbiome_stats.csv file. This file contains 48197 rows. According to the authors, the file includes data from both existing files and new data. As a result, it is difficult to determine which data have been reproduced. Even when using a Ctrl+F search for the reported p-value (e.g, pvalue = 0.134) in the Excel file, this value appears in several rows labeled under different configurations such as (label1/label2):
--- chunk_size3000/sync_chunkN_presp0.0;
--- chunk_size3000/sync_chunkN_temp1.5;
--- chunk_size5000/gpt4-0613;
--- machine/ sync_chunkY_topp0.0, etc…
5.3 Statistical comparison Original vs Reproduced results
- Results: The biome_subbiome_stats.csv file was reproduced, but it is difficult to distinguish between the newly reproduced data and the existing data already present in the file. Additionally, the data presented in the manuscript are also hard to identify due to the size of the file. No comparison was performed.
- Comments: -
- Errors detected: Authors identified an error in the manuscript text during the review with the first adjusted p-value that is not 0.031 but 0.134.
- Statistical Consistency: No comparison was performed.
----
6. â€‹â€‹Conclusion
- Summary of the computational reproducibility review
The main scripts to reproduced the results were successfully executed and the output files were generated. However, due to the size of the output files and the lack of precise references in the manuscript, it was difficult to identify which parts of the output correspond to the results presented in the paper. Moreover, authors mentionned that the script adds data to existing output files rather than generating new ones, making it hard to distinguish between old and new data. This led to confusion when trying to compare the reproduced results with those in the manuscript. Then a comparison of statistical values was not possible.
- Recommendations for authors
To improve the reproducibility of the manuscript, we recommend the authors to:
-- Clarify instructions in the README about the MicrobeAtlasProject folder.
-- Ensure scripts generate new outputs or clarify which data is new vs. existing in the files with for example a column indicating the origin (e.g., "new" or "existing").
-- Link results in the manuscript to specific rows/sections in the output files to easily locate the exact data used. Another solution could be to consider including a smaller, or a filtered version of the output

files with only the rows used for key reults, figures or tables, to make checking results easier and avoid error.
-- Metadata: For the data used or generated by the scripts, it would be helpful to include accompanying metadata files that explain:
--- The definition of each variable name.
--- The origin of each dataset (raw, processed, etc).
--- Any preprocessing steps applied before analysis.

Methods

Are the methods appropriate to the aims of the study, are they well described, and are necessary controls included? Choose an item.

Conclusions

Are the conclusions adequately supported by the data shown? Choose an item.

Reporting Standards

Does the manuscript adhere to the journal's guidelines on [minimum standards of reporting?]() Choose an item.

Choose an item.

Statistics

Are you able to assess all statistics in the manuscript, including the appropriateness of statistical tests used? Choose an item.

Quality of Written English

Please indicate the quality of language in the manuscript: Choose an item.

Declaration of Competing Interests

Please complete a declaration of competing interests, considering the following questions:

- Have you in the past five years received reimbursements, fees, funding, or salary from an organisation that may in any way gain or lose financially from the publication of this manuscript, either now or in the future?
- Do you hold any stocks or shares in an organisation that may in any way gain or lose financially from the publication of this manuscript, either now or in the future?
- Do you hold or are you currently applying for any patents relating to the content of the manuscript?

- Have you received reimbursements, fees, funding, or salary from an organization that holds or has applied for patents relating to the content of the manuscript?
- Do you have any other financial competing interests?
- Do you have any non-financial competing interests in relation to this paper?

If you can answer no to all of the above, write 'I declare that I have no competing interests' below. If your reply is yes to any, please give details below.

1. No 2. No 3. No 4. No 5. No 6. No

I agree to the open peer review policy of the journal. I understand that my name will be included on my report to the authors and, if the manuscript is accepted for publication, my named report including any attachments I upload will be posted on the website along with the authors' responses. I agree for my report to be made available under an Open Access Creative Commons CC-BY license (http://creativecommons.org/licenses/by/4.0/). I understand that any comments which I do not wish to be included in my named report can be included as confidential comments to the editors, which will not be published.

Choose an item.

To further support our reviewers, we have joined with Publons, where you can gain additional credit to further highlight your hard work (see: https://publons.com/journal/530/gigascience). On publication of this paper, your review will be automatically added to Publons, you can then choose whether or not to claim your Publons credit. I understand this statement.

Yes Choose an item.
