# Supplementary material for: Enhanced semantic classification of microbiome sample origins using large language models (LLMs)
Source: Gigascience. 2026 Feb 12;15:giag015. doi: 10.1093/gigascience/giag015 (PMC13042274; doi:10.1093/gigascience/giag015)
Supplement: giag015_Reviewer_2_Report_revision_1 — Reviewer 2 -- 11/10/2025 [file giag015_reviewer_2_report_revision_1.pdf]

Reviewer Report

Title: Enhanced semantic classification of microbiome sample origins using Large Language Models (LLMs)

Version: Revision 1 **Date:** 11/10/2025

Reviewer name: Reviewer 2

Reviewer Comments to Author:

Reproducibility report for: Enhanced semantic classification of microbiome sample origins using Large Language Models
Journal: Gigascience
ID number/DOI: GIGA-D-25-00316
Reviewer(s): Laura Caquelin, Department of Clinical Neuroscience, Karolinska Institutet, Sweden [Wrote the report and reproduced the results]
Gustav Nilsonne, Department of Clinical Neuroscience, Karolinska Institutet, Sweden [Reviewed the final report]
----

1. Context
This report corresponds to a second assessment of the computational reproducibility of the article GIGA-D-25-00316, following a revision by the authors after the first round of review.
The scope of the computational reproducibility review is to reproduce the results presented in section "Human versus GPT classification accuracy" (pages 18-19), related to the evaluation of GPT's annotation performance compared to a human expert when classifying environmental sequencing samples into biomes and sub-biomes.

----

2. Changes since the first review
The authors made several changes based on comments from the initial computational reproducibility review:
- Updated the README file
- Edited the overall_analysis.py and validate_biomes_subbiomes.py scripts.
- Updated the Zenodo repository.
- Updated several results in the manuscript because the authors removed 20 GPT output files from the analyses.

----

3. Availability of Materials
a. Data
- Data availability: Open
- Data completeness: Complete = all data necessary to reproduce main results are available
- Access Method: Repository
- Repository: https://doi.org/10.5281/zenodo.17437043

b. Code
- Code availability: Open
- Programming Language(s): Python
- Repository link:
https://github.com/GaioTransposon/metadata_mining/tree/main/scripts
- License: MIT License
- Repository status: Public
- Documentation: Clear Readme file
----
4. Computational environment of reproduction analysis
- Operating system for reproduction: MacOS 15.6.1
- Programming Language(s): Python
- Code implementation approach: Using shared code
- Version environment for reproduction: Python 3.13.7
----
5. Results
5.1 Original study results
- Results 1: Authors have updated the text related to the scope of the computational reproducibility review after the first review:
"There was an improvement in accuracy between the human's first attempt (with the initial prompt) and the human's second attempt (better prompt) (adj p-valueâ‰¤0.001). No significantly different performances were detected for the sub-biome classification, neither between GPT and the human, nor between prompt versions (adj p-value=1)." Section "Human versus GPT classification accuracy", lines 457-461.
5.2 Steps for reproduction
-> Set up the environment: All the steps describe in the readme file were followed.
-> Run only the LLM runs evaluation in container 4: validate_biomes_subbiomes.py.
5.3 Statistical comparison Original vs Reproduced results
- Reproduced results:
See screenshot of a part of the file biome_subbiome_results__text-embedding-3-small.csv.
See screenshot of a part of the file biome_subbiome_stats__text-embedding-3-small.csv.
- Comments: -
- Errors detected: No errors were detected in the "Human versus GPT classification accuracy" section
- Statistical Consistency: The adjusted p-value reported for the biome validation (adj p-value â‰¤0.001) and the sub-biome validation (adj p-value=1) are consistent with the reproduced adjusted p-values.
----
6. Conclusion
- Follow-up on previous recommendations
In the first round of review, we noted several recommendations about:
-- Clarify instructions in the README: The authors improved the README file for better clarity.
-- Ensure scripts generate new outputs and link results to the manuscript: The authors specified in the Methods section which scripts correspond to each analysis, and simplified the outputs. It is now easier

to locate in the output files the results reported in the manuscript.
-- Add metadata: The authors added the file Files_list_Zenodo_v2.xlsx, which describes each file in the repository.
- Summary of the second computational reproducibility review
The scope of this review was to reproduce the results presented in the "Human versus GPT classification accuracy" section of the manuscript (pages 18-19). The results of this section were fully reproducible. All main statistical values were consistent between the reproduced and original analyses.

Methods

Are the methods appropriate to the aims of the study, are they well described, and are necessary controls included? Choose an item.

Conclusions

Are the conclusions adequately supported by the data shown? Choose an item.

Reporting Standards

Does the manuscript adhere to the journal's guidelines on minimum standards of reporting? Choose an item.

Choose an item.

Statistics

Are you able to assess all statistics in the manuscript, including the appropriateness of statistical tests used? Choose an item.

Quality of Written English

Please indicate the quality of language in the manuscript: Choose an item.

Declaration of Competing Interests

Please complete a declaration of competing interests, considering the following questions:

- Have you in the past five years received reimbursements, fees, funding, or salary from an organisation that may in any way gain or lose financially from the publication of this manuscript, either now or in the future?
- Do you hold any stocks or shares in an organisation that may in any way gain or lose financially from the publication of this manuscript, either now or in the future?
- Do you hold or are you currently applying for any patents relating to the content of the manuscript?

- Have you received reimbursements, fees, funding, or salary from an organization that holds or has applied for patents relating to the content of the manuscript?
- Do you have any other financial competing interests?
- Do you have any non-financial competing interests in relation to this paper?

If you can answer no to all of the above, write 'I declare that I have no competing interests' below. If your reply is yes to any, please give details below.

1. No 2. No 3. No 4. No 5. No 6. No

I agree to the open peer review policy of the journal. I understand that my name will be included on my report to the authors and, if the manuscript is accepted for publication, my named report including any attachments I upload will be posted on the website along with the authors' responses. I agree for my report to be made available under an Open Access Creative Commons CC-BY license (http://creativecommons.org/licenses/by/4.0/). I understand that any comments which I do not wish to be included in my named report can be included as confidential comments to the editors, which will not be published.

Choose an item.

To further support our reviewers, we have joined with Publons, where you can gain additional credit to further highlight your hard work (see: https://publons.com/journal/530/gigascience). On publication of this paper, your review will be automatically added to Publons, you can then choose whether or not to claim your Publons credit. I understand this statement.

Yes Choose an item.
