# Supplementary material for: Enhanced semantic classification of microbiome sample origins using large language models (LLMs)
Source: Gigascience. 2026 Feb 12;15:giag015. doi: 10.1093/gigascience/giag015 (PMC13042274; doi:10.1093/gigascience/giag015)
Supplement: giag015_Reviewer_3_Report_original_submission — Reviewer 3 -- 9/14/2025 [file giag015_reviewer_3_report_original_submission.pdf]

Reviewer Report

Title: Enhanced semantic classification of microbiome sample origins using Large Language Models (LLMs)

Version: Original Submission **Date:** 9/14/2025

Reviewer name: Reviewer 3

Reviewer Comments to Author:

The ability to reuse scientific data for secondary analysis is an extremely important topic. Since the promotion of the FAIR Guiding Principles a decade ago, the central importance of standards-adherent metadata has received considerable attention. Although this paper surprisingly doesn't mention the FAIR principles, the work is important in understanding what it takes to make datasets FAIR and "AI ready."

A core problem with the paper is that it is unsure who its audience is. The paper is motivated by the needs to scientists to search for and reuse online datasets for secondary analysis, but much of the paper concerns highly technical issues that are related to fine tuning LLM performance. It is laudable that the manuscript annotates its discussion of the authors' methods with pointers to actual Python scripts that would allow third parties to replicate the authors' work. The detailed presentation, however, may make it hard for many readers to understand the computational strategy that all the scripts are implementing. The organization of the paper weaves from discussions of the ability of LLMs to extract scientific standards from "legacy" experimental metadata to details of how to enhance computational efficiency to make the use of LLMs more cost-effective. The title and abstract of the paper suggest that the authors are aiming for a more scientific audience, but much of the manuscript focuses on arcane implementation details that will be less important to such readers.

Missing from the paper is a detailed discussion of what the metadata in SRA are really like. The reader never sees complete examples of the metadata that are processed in the authors' work, and thus it is hard to have intuition about the problem that the authors are trying to solve. In particular, the paper doesn't present information about the range of attributes in user-defined metadata fields in SRA. The paper would benefit from a discussion of the structure of scientific metadata in general, and of how the authors' work fits into the larger effort in the research community to make datasets FAIR. (Full disclosure: My own laboratory is involved in such activity. See https://arxiv.org/abs/2504.05307v2)

The abstract of the paper states that the authors "test to what extent LLMs can be used to cost-effectively automate the re-annotation of sequencing records." Alas, the paper really examines re-annotation of only the fields for "biome" and "location." A weakness of the paper is that the reader doesn't learn what other fields may be relevant in these metadata records, and why the authors chose to focus on the particular fields that they studied. Overall, much more attention should be placed on discussion of the limitations of the work and how well the results might scale to more general problems in standardization of scientific metadata.

Minor comments:

The term "biome" is never well defined.

Frequently, parenthetical remarks begin with "e.g." and end with "etc." This style is redundant; you need only one of these abbreviations in each instance.
Page 6, para 1: "last three digits" or "last three characters"?
What is the motivation for consolidating reference ontologies into a single dictionary?
Page 13, para 2: The notion of "lenient matches" requires much more discussion. If the goal is to make the legacy metadata standards-adherent, then a "lenient" match would not seem to be valid. The operative question is, "What metadata terms will users invoke to search for datasets?", and presumably users will be searching for standard terms only.
Page 17, para 3: It's unclear what is meant by "most samples have fewer misclassifications." Fewer than what?
Page 24, para 2: It's not clear what you mean when you say, "In half of the cases GPT correctly predicted the location, while the lat/lon coordinates parsed from the metadata were incorrect." Are you saying that GPT gave correct results when the lat/lon data were incorrect?
Figure 1 is very busy and the tiny font is hard to read.

Methods

Are the methods appropriate to the aims of the study, are they well described, and are necessary controls included? Choose an item.

Conclusions

Are the conclusions adequately supported by the data shown? Choose an item.

Reporting Standards

Does the manuscript adhere to the journal's guidelines on minimum standards of reporting? Choose an item.

Choose an item.

Statistics

Are you able to assess all statistics in the manuscript, including the appropriateness of statistical tests used? Choose an item.

Quality of Written English

Please indicate the quality of language in the manuscript: Choose an item.

Declaration of Competing Interests

Please complete a declaration of competing interests, considering the following questions:

- Have you in the past five years received reimbursements, fees, funding, or salary from an organisation that may in any way gain or lose financially from the publication of this manuscript, either now or in the future?
- Do you hold any stocks or shares in an organisation that may in any way gain or lose financially from the publication of this manuscript, either now or in the future?
- Do you hold or are you currently applying for any patents relating to the content of the manuscript?
- Have you received reimbursements, fees, funding, or salary from an organization that holds or has applied for patents relating to the content of the manuscript?
- Do you have any other financial competing interests?
- Do you have any non-financial competing interests in relation to this paper?

If you can answer no to all of the above, write 'I declare that I have no competing interests' below. If your reply is yes to any, please give details below.

I declare that I have no competing interests.

I agree to the open peer review policy of the journal. I understand that my name will be included on my report to the authors and, if the manuscript is accepted for publication, my named report including any attachments I upload will be posted on the website along with the authors' responses. I agree for my report to be made available under an Open Access Creative Commons CC-BY license (http://creativecommons.org/licenses/by/4.0/). I understand that any comments which I do not wish to be included in my named report can be included as confidential comments to the editors, which will not be published.

Choose an item.

To further support our reviewers, we have joined with Publons, where you can gain additional credit to further highlight your hard work (see: https://publons.com/journal/530/gigascience). On publication of this paper, your review will be automatically added to Publons, you can then choose whether or not to claim your Publons credit. I understand this statement.

Yes Choose an item.
