# Supplementary material for: Enhanced semantic classification of microbiome sample origins using large language models (LLMs)
Source: Gigascience. 2026 Feb 12;15:giag015. doi: 10.1093/gigascience/giag015 (PMC13042274; doi:10.1093/gigascience/giag015)
Supplement: giag015_Reviewer_3_Report_revision_1 — Reviewer 3 -- 11/28/2025 [file giag015_reviewer_3_report_revision_1.pdf]

Reviewer Report

Title: Enhanced semantic classification of microbiome sample origins using Large Language Models (LLMs)

Version: Revision 1 **Date:** 11/28/2025

Reviewer name: Reviewer 3

Reviewer Comments to Author:

The authors have done an excellent job responding to the previous review. In many ways, this reads like a new paper.

There is a pressing need to convert legacy metadata to a standardized form purposes of searching datasets and for data reuse. This paper presents an important data point among recent efforts to use LLMs to provide that transformation. The manuscript still tends to be inconsistent in stating claims about the authors' contribution. For example, the abstract claims "here, we test to what extent large language models (LLMs) can be used to cost-effectively automate the re-annotation of sequencing records" (Line 14), when, in actuality, the authors address only two metadata attributes of these records. Biome and geolocation may indeed be the most interesting metadata attributes for the authors to study, but the paper still does not help us to understand what the remaining attributes are and what challenges may exist in standardizing those fields.

As the authors note, there have been several recent papers by groups attempting to pick at this important problem, with each paper providing a bit more information. It would be wonderful if the authors' discussion could take a step back and looks at this overall research paradigm, and to comment on what is needed in future work by the community to address the metadata-rectification problem overall in ways that go beyond the current work on microbiome samples.

In sum, the paper is much improved and makes a nice contribution.

Minor comments:

Line 95: Please don't anthropomorphize by suggesting that LLMs "understand" anything.

Line 130: "LLMs" should be "LLM".

Line 131: Change "had" to "has".

Line 159: Move the period to the end of the line. Same for lines 173, 296, 311, and 674.

Line 169: Delete the word "sufficiently," unless you are going to explain in what ways the samples were distinct.

Line 715: It makes no sense to cite "Sundaram et al. (2025)" with an APA-style citation and then to give a numeric citation at the end of the sentence. Delete the year. Ditto for line 722.

Methods

Are the methods appropriate to the aims of the study, are they well described, and are necessary controls included? Choose an item.

Conclusions

Are the conclusions adequately supported by the data shown? Choose an item.

Reporting Standards

Does the manuscript adhere to the journal's guidelines on minimum standards of reporting? Choose an item.

Choose an item.

Statistics

Are you able to assess all statistics in the manuscript, including the appropriateness of statistical tests used? Choose an item.

Quality of Written English

Please indicate the quality of language in the manuscript: Choose an item.

Declaration of Competing Interests

Please complete a declaration of competing interests, considering the following questions:

- Have you in the past five years received reimbursements, fees, funding, or salary from an organisation that may in any way gain or lose financially from the publication of this manuscript, either now or in the future?
- Do you hold any stocks or shares in an organisation that may in any way gain or lose financially from the publication of this manuscript, either now or in the future?
- Do you hold or are you currently applying for any patents relating to the content of the manuscript?
- Have you received reimbursements, fees, funding, or salary from an organization that holds or has applied for patents relating to the content of the manuscript?
- Do you have any other financial competing interests?
- Do you have any non-financial competing interests in relation to this paper?

If you can answer no to all of the above, write 'I declare that I have no competing interests' below. If your reply is yes to any, please give details below.

I declare that I have no competing interests.

I agree to the open peer review policy of the journal. I understand that my name will be included on my report to the authors and, if the manuscript is accepted for publication, my named report including any attachments I upload will be posted on the website along with the authors' responses. I agree for my

report to be made available under an Open Access Creative Commons CC-BY license (http://creativecommons.org/licenses/by/4.0/). I understand that any comments which I do not wish to be included in my named report can be included as confidential comments to the editors, which will not be published.

Choose an item.

To further support our reviewers, we have joined with Publons, where you can gain additional credit to further highlight your hard work (see: https://publons.com/journal/530/gigascience). On publication of this paper, your review will be automatically added to Publons, you can then choose whether or not to claim your Publons credit. I understand this statement.

Yes Choose an item.
